# Heterogeneous Catalysts for Glycerol Biorefineries: Hydrogenolysis to 1,2-Propylene Glycol

**DOI:** 10.3390/ma16093551

**Published:** 2023-05-05

**Authors:** Martín N. Gatti, Federico M. Perez, Gerardo F. Santori, Nora N. Nichio, Francisco Pompeo

**Affiliations:** 1 Centro de Investigación y Desarrollo en Ciencias Aplicadas (CINDECA), Facultad de Ciencias Exactas, Universidad Nacional de La Plata (UNLP)—CONICET, Calle 47, 257, La Plata 1900, Argentina; martin.gatti@ing.unlp.edu.ar (M.N.G.); federico.perez@ing.unlp.edu.ar (F.M.P.); santori@quimica.unlp.edu.ar (G.F.S.); nnichio@quimica.unlp.edu.ar (N.N.N.); 2 Facultad de Ingeniería, Universidad Nacional de La Plata (UNLP), Calle 1 esq. 47, La Plata 1900, Argentina

**Keywords:** heterogeneous catalysts, glycerol, biodiesel, hydrogenolysis, 1,2-propylene glycol

## Abstract

Research on the use of biomass resources for the generation of energy and chemical compounds is of great interest worldwide. The development and growth of the biodiesel industry has led to a parallel market for the supply of glycerol, its main by-product. Its wide availability and relatively low cost as a raw material make glycerol a basic component for obtaining various chemical products and allows for the development of a biorefinery around biodiesel plants, through the technological integration of different production processes. This work proposes a review of one of the reactions of interest in the biorefinery environment: the hydrogenolysis of glycerol to 1,2-propylene glycol. The article reviews more than 300 references, covering literature from about 20 years, focusing on the heterogeneous catalysts used for the production of glycol. In this sense, from about 175 catalysts, between bulk and supported ones, were revised and discussed critically, based on noble metals, such as Ru, Pt, Pd, and non-noble metals as Cu, Ni, Co, both in liquid (2–10 MPa, 120–260 °C) and vapor phase (0.1 MPa, 200–300 °C). Then, the effect of the main operational and decision variables, such as temperature, pressure, catalyst/glycerol mass ratio, space velocity, and H_2_ flow, are discussed, depending on the reactors employed. Finally, the formulation of several kinetic models and stability studies are presented, discussing the main deactivation mechanisms of the catalytic systems such as coking, leaching, and sintering, and the presence of impurities in the glycerol feed. It is expected that this work will serve as a tool for the development of more efficient catalytic materials and processes towards the future projection of glycerol biorefineries.

## 1. Introduction

**Figure 1:**
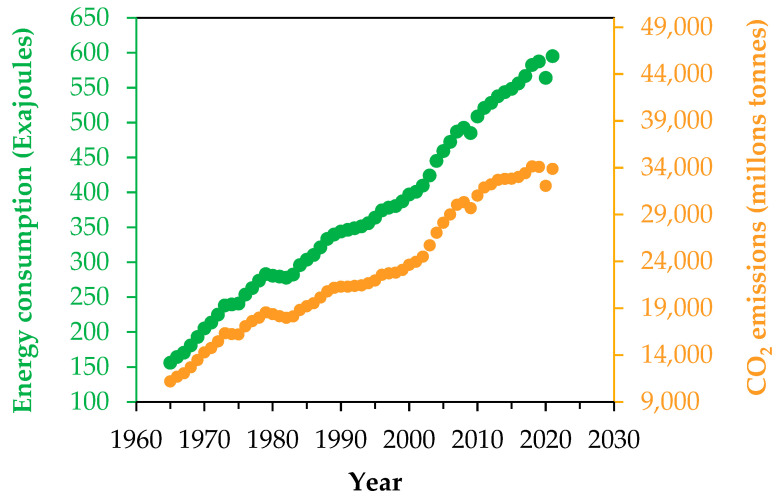
Historical evolution of world energy consumption and CO_2_ emissions.

It is well known that energy is essential for the economic and social development of any country, and its demand has now been steadily increasing for several decades. Since the main sources of energy used are coal, oil, and natural gas, the accumulation of carbon dioxide (CO_2_) and other gases in the atmosphere has reached unsustainable levels, following the same growth trend as the energy demands of the world’s population (Figure 1) [1].

Carbon dioxide accumulation in the atmosphere adds to the concern about the unequal distribution of fossil fuels, which has led researchers around the world to join efforts to study and develop new ways of obtaining energy and essential products for daily life, in order to facilitate their access and ensure their sustainable exploitation.

In this context, the concept of renewable energies arises, such as hydroelectric, geothermal, solar (thermal and photovoltaic), wind, marine, and biomass [2]. Within this group, biomass sources come from livestock, agriculture, forests, and urban wastewater and include all organic matter from plants, as well as organic waste from humans, animals, aquatic life, and plants.

Biomass sources allow for obtaining not only energy, such as heat and electricity through thermal conversion processes, but also gaseous fuels, such as methane and hydrogen, and liquid fuels such as ethanol and biodiesel. In particular, the production of liquid biofuels has been one of the most developed ways of valorizing biomass at an international level, being exploited mainly by those countries with a large availability of biomass.

**Scheme 1:**
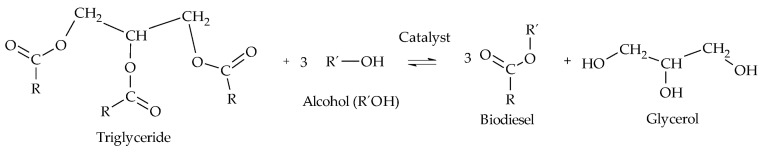
Biodiesel production from transesterification of triglycerides with alcohols.

Biodiesel has been one of the most developed biofuels, composed of a mixture of fatty acid esters produced through the transesterification of triglycerides in the presence of catalysts [3]. In the transesterification reaction, triglycerides react with a short-chain alcohol (R’OH) (R’ = CH_3_-/CH_3_CH_2_-/CH_3_CH_2_CH_2_-), in the presence of a catalyst, producing three molecules of fatty acid esters and one molecule of glycerol (Scheme 1). Since the reaction is thermodynamically limited, an excess of alcohol is used to shift the equilibrium towards product formation [4].

**Figure 2:**
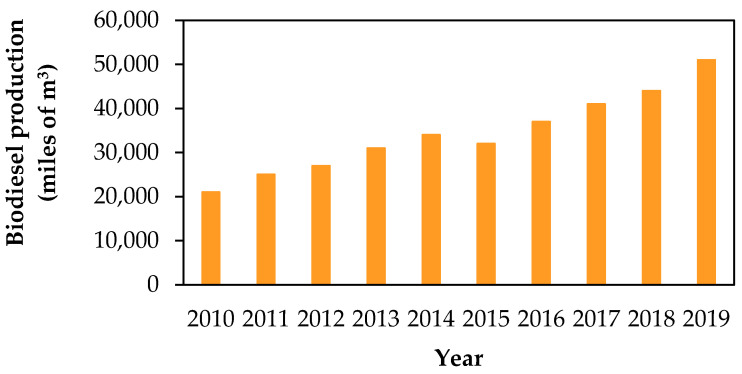
Annual evolution of world biodiesel production.

In numerical terms, global biodiesel production has experienced an increase of 928% between 2000 and 2019 (Figure 2) [5]. This behavior is due to the policies implemented by governments to encourage the use of biofuels, such as the establishment of a mandatory percentage in diesel, preferential taxes, and some subsidies, added to the increased demand for fuels.

**Figure 3:**
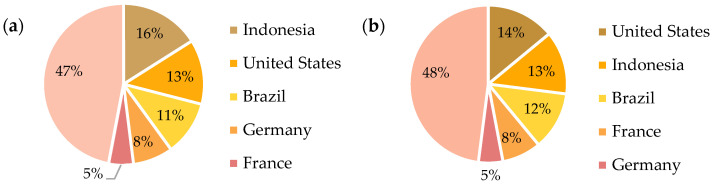
(**a**) World distribution of biodiesel-producing countries. (**b**) World distribution of biodiesel-consuming countries.

The five main biodiesel producers in the world are Indonesia (16%), the United States (13%), Brazil (11%), Germany (8%), and France (5%). The remaining 47% of production is distributed in other countries of the world, with the significant participation of Argentina, Spain, the Netherlands, Thailand, and Malaysia (Figure 3a) [5].

Furthermore, biodiesel consumption is relatively decentralized: the United States, Indonesia, Brazil, France, and Germany account for 14%, 13%, 12%, 12%, 8%, and 5% of the world total, respectively. The remaining 48% is distributed among the other countries of the world, with significant shares from Spain, Sweden, Thailand, the United Kingdom, Italy, and Argentina (Figure 3b) [5]. The demand for biodiesel is expected to continue to increase, especially in developing countries, given policies aimed at increasing the mandatory percentage in traditional fuels.

From a stoichiometric point of view, 100 kg of vegetable oil and 10 kg of alcohol are required to produce 100 kg of biodiesel. This process generates 10 kg of glycerol as a by-product. This glycerol, which accompanies the biodiesel produced, has a purity that varies in the range of 55–90 wt.% and is called crude glycerol. Among the impurities found are traces of unreacted triglycerides and monoalcohols, biodiesel, soaps, and other minority contaminants [6].

Due to its low level of purity, crude glycerol is not suitable for application in fine chemicals, pharmaceuticals, and the agri-food market, and requires treatment and purification [7]. Currently, crude glycerol is subjected to fractional distillation to obtain technical grade glycerol. However, neither the pharmaceutical nor the agri-food market can absorb all the glycerol produced. This surplus has oversaturated the market, devaluing the price of glycerol. Because of this, the search for new alternatives for glycerol is very attractive [8].

**Figure 4:**
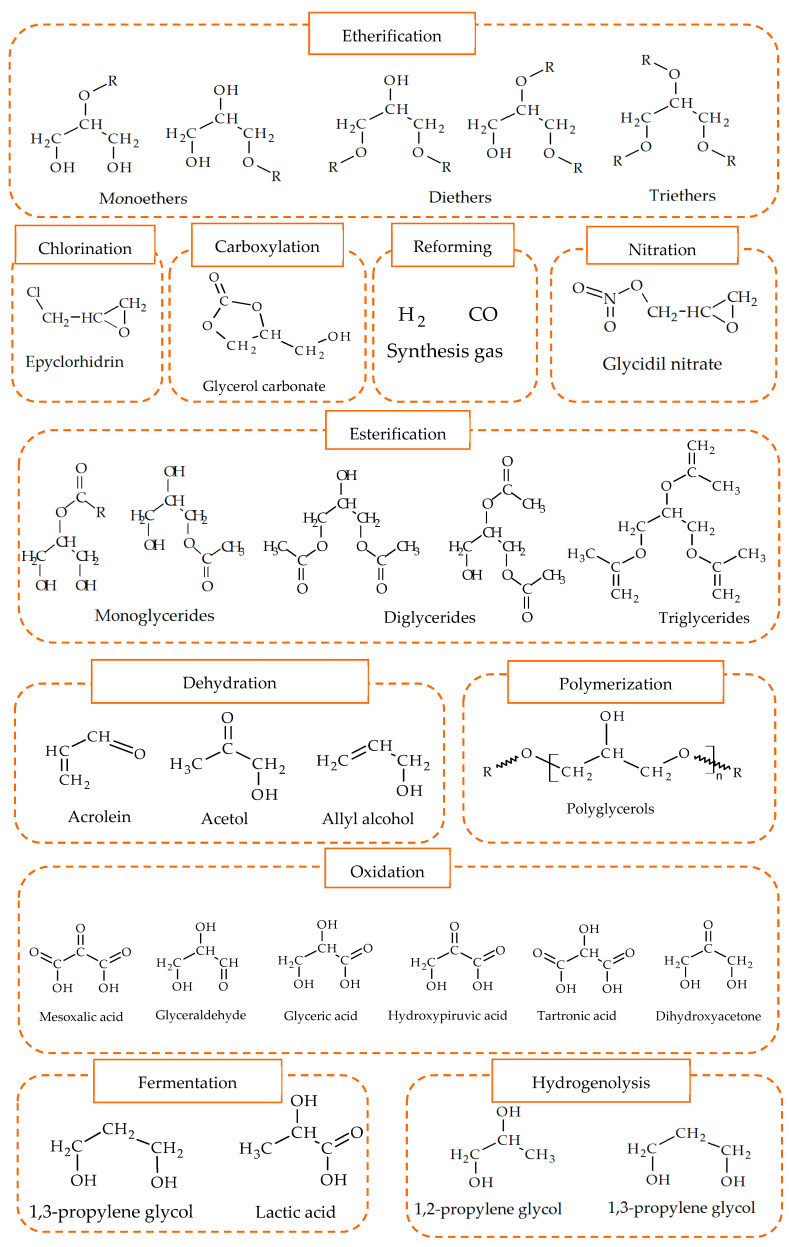
Glycerol conversion reactions for valorization.

Due to its reactivity, the glycerol molecule can be used as a precursor molecule for many other chemical compounds of industrial interest, through different reactions (Figure 4), such as oxidation [9], nitration [10], polymerization [11], esterification [12], etherification [13], reforming [14], chlorination [15], carbonation [16], dehydration [17], and hydrogenolysis [18].

**Figure 5:**
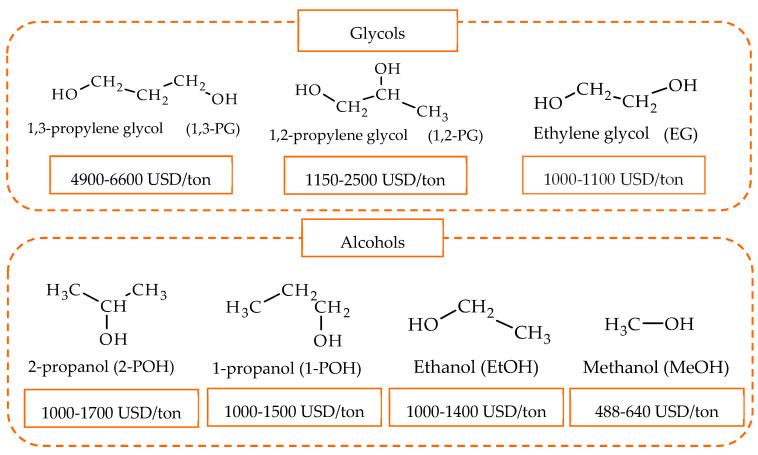
Main products of glycerol hydrogenolysis and their market prices.

Of all the mentioned processes, catalytic hydrogenolysis is an interesting alternative because it leads to the formation of 1,2-propylene glycol (1,2-PG) and other glycols such as 1,3-propylene glycol (1,3-PG) and ethylene glycol (EG), widely used in the chemical industry. In addition, short-chain alcohols, such as 1-propanol (1-POH), 2-propanol (2-POH), ethanol (EtOH), and methanol (MeOH), can be generated. Compared to the price of crude glycerol on the market (500–800 USD/ton), any of the hydrogenolysis products mentioned above is of greater added value than glycerol; however, the most valuable ones are 1,3-PG, 1,2-PG, 2-POH, and 1-POH (Figure 5) [19].

**Figure 6:**
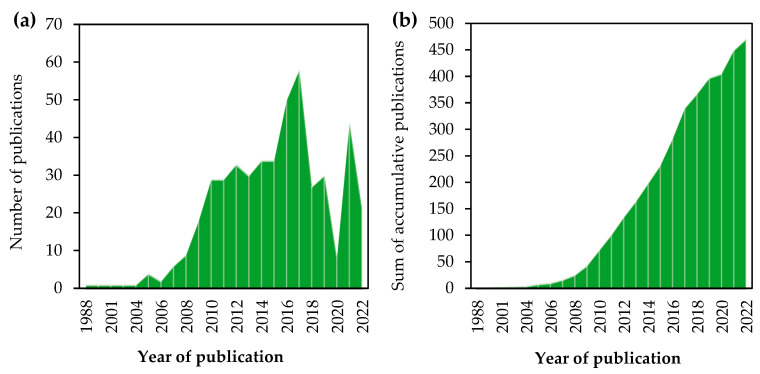
(**a**) Annual number of articles published on hydrogenolysis of glycerol obtained from the Scopus Database (“hydrogenolysis of glycerol” as key words). (**b**) Cumulative number of articles published until 2022. Statistical analysis obtained from the articles in Scopus Database.

From its inception to the present, glycerol hydrogenolysis using catalysts has been reported in more than 450 research articles. Although the number of articles published annually has fluctuated over time (Figure 6a), the number of accumulated publications on the subject has grown exponentially (Figure 6b), accompanying the growing interest in the search for glycerol conversion routes to high value-added products.

**Figure 7:**
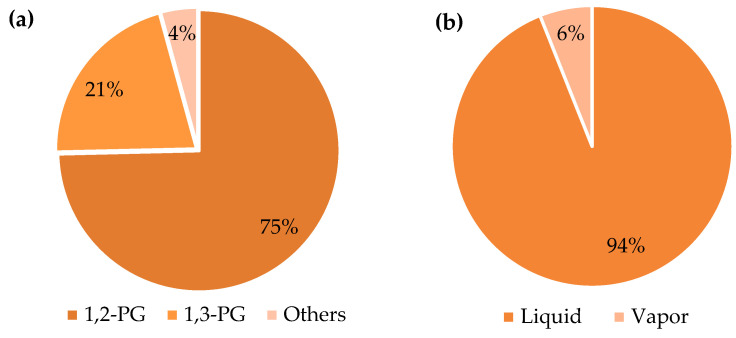
(**a**) Distribution of articles by type of product. (**b**) Distribution of articles focused on the production of 1,2-PG according to the operation phase. Statistical analysis obtained from the articles in the Scopus Database.

A statistical analysis of the total number of articles published to date indicates that 75% focus on the production of 1,2-PG, while 21% investigate the production of 1,3-PG, and 4% the production of EG and alcohols such as 1-POH and 2-POH (Figure 7a). Furthermore, within the set of works investigating the production of 1,2-PG, 94% employ reactors operating in the liquid phase, while only 6% study its production in the vapor phase (Figure 7b).

**Figure 8:**
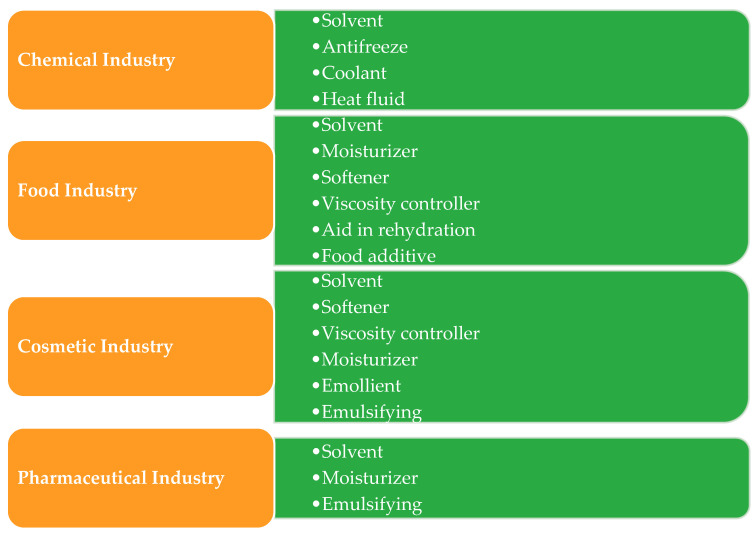
Main applications of 1,2-PG.

1,2-PG is a viscous, colorless, odorless liquid at ambient conditions and is completely soluble in water and polar compounds. It is commercially available in industrial and high purity grades, called USP/EP grade. Due to its practically zero toxicity, it can be used in the chemical, food, cosmetics, and pharmaceutical industries (Figure 8) [20].

**Figure 9:**
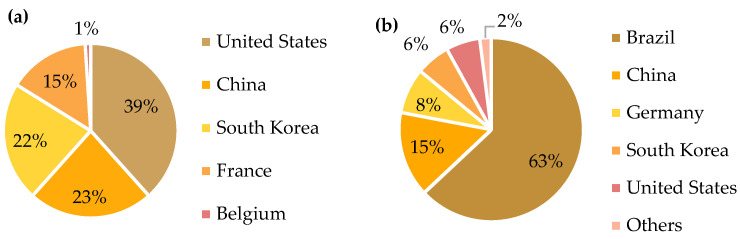
(**a**) Global distribution of 1,2-PG producing countries. (**b**) Global distribution of 1,2-PG importing countries.

The world supply of 1,2-PG is led by Asia-Pacific, with China (23%) and South Korea (22%) being the main producing countries, followed by North America, where the producer is the United States (38%), and finally the European region, led by France (15%) and Belgium (2%) (Figure 9a). Regarding consumption, Brazil is the largest importer of 1,2-PG (63%), followed by China (15%) and Germany (8%), while South Korea (6%) and the United States (6%) are the countries that import the least 1,2-PG due to their high local production (Figure 9b) [21,22].

**Scheme 2:**
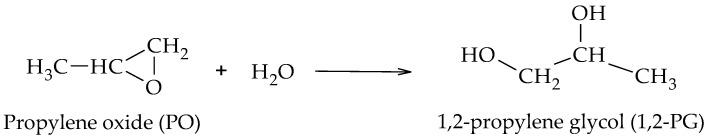
Formation reaction of 1,2-PG from PO.

At the industrial level, 1,2-PG is produced at 125 °C and 2 MPa from the hydration of propylene oxide (PO), which is obtained via petrochemicals (Scheme 2).

**Scheme 3:**
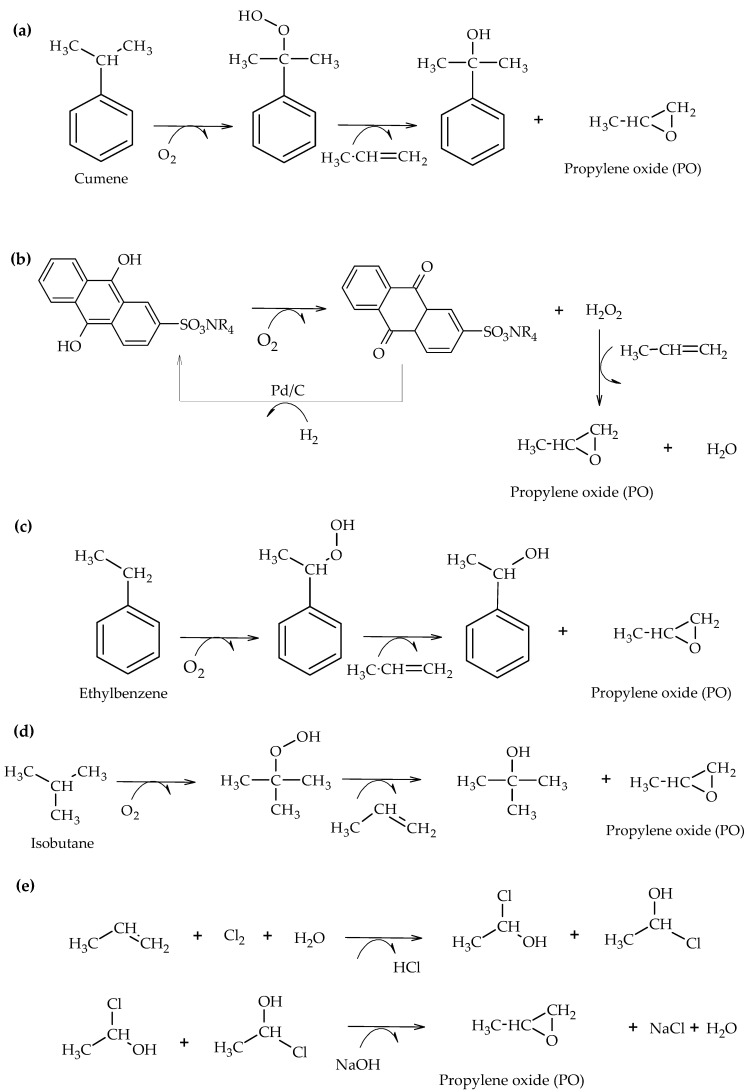
Technologies for PO production from (**a**) cumene hydroperoxide, (**b**) hydrogen peroxide, (**c**) propylene oxide/styrene monomer, (**d**) propylene oxide/t-butyl alcohol, (**e**) chlorohydrin.

There are currently five technologies for PO production [23]: (a) the cumene hydroperoxide process (Sumitomo Chemical Company, Tokyo, Japan); (b) epoxidation with hydrogen peroxide (Dow, Midland, USA-BASF, Ludwigshafen, Germany); (c) the propylene oxide/styrene monomer process (Lyondell Basel, Rotterdam, Netherlands-Shell Company, London, United Kingdom); (d) the propylene oxide/t-butyl alcohol process (Lyondell Basel, Rotterdam, Netherlands-Huntsman Corporation, Texas, USA); and (e) the chlorohydrin process (Dow, Midland, USA). The reactions involved in the above processes are presented in Scheme 3.

Of the five technologies mentioned, the most employed ones are (d) and (e), although the latter has as its main disadvantage the use of Cl_2_ gas, which, besides being toxic and corrosive, is expensive [20]. Direct epoxidation of propene with gaseous oxygen in the presence of Ag and Au catalysts has also been studied, but this alternative is not yet viable because it requires improvements in the selectivity and stability of the catalysts [23].

Of all the world production, it is estimated that only 10% of 1,2-PG is produced from glycerol. The remaining 90% is still produced from the hydration of propylene oxide, i.e., petrochemically [20]. The production of 1,2-PG from the hydrogenolysis of glycerol would offer an alternative route to the current processes based on the principles of green chemistry, since it would be possible to carry it out using renewable resources with the design of suitable catalysts.

Given the interest in the field of hydrogenolysis research, several review articles have been published, some general [24,25,26,27] and others aimed at the specific production of 1,2-PG [28,29], 1,3-PG [18,30,31,32,33,34], EG [35], and other compounds of interest such as 1-POH, propylene, and allyl alcohol [36,37] whose production involves catalytic systems with more sophisticated structural properties.

In the early days, Nakagawa et al. provided a review work on heterogeneous catalysts for glycerol hydrogenolysis in both the liquid and vapor phase, distinguishing between those based on noble and non-noble metals and their employment in the hydrogenolysis reaction [24].

A second review published by Martin et al. incorporated the concept of H_2_ generated in situ in the reaction medium through the combination with the liquid phase glycerol-reforming reaction and the participation of H_2_ donor molecules [25], with an introduction to the possible mechanisms of the hydrogenolysis reaction. Feng et al. then completed the review of the hydrogenolysis reaction mechanisms by linking each of them to the catalysts used and the operating conditions under which the reaction is carried out [26].

Sometime later, Vasiliadou et al. published a review on engineering aspects linked to the hydrogenolysis reaction, taking into account not only the reaction mechanisms, but also the kinetic models proposed in the literature up to that time and the effect of some operating variables in the process, such as temperature, pressure, and glycerol concentration [27].

At this point, the review articles begin to be selectively oriented towards the specific production of different compounds obtained from the hydrogenolysis of glycerol. The first to publish these reports were Sun et al., who differentiated the catalysts used for the selective production of 1,2-PG, 1,3-PG, propanols, propylene, and allyl alcohol [36]. Years later, Nakagawa et al. published a similar review [37]. Subsequently, the first review papers on the production of 1,3-PG began to appear, discussing the results of some catalytic tests [30,31]. Then, a series of reviews focused their attention on the catalysts that showed promise for obtaining 1,3-PG based on Pt-W [32], the role of W species in these systems [33], and the electronic and surface acid–metal effects [38]. Some review articles on advances in this subject have recently been published [18,34].

With respect to the production of 1,2-PG, there are fewer review articles and they do not review all aspects of the subject. Nanda et al. reported a review emphasizing the effect of catalyst preparation and their activation protocols for catalysts based on noble metals, non-noble metals, and their appropriate combination. Some aspects linked to the deactivation of these catalytic systems and the use of crude glycerol as feedstock are discussed in this article [28]. Zhao et al. in turn provided a mini-review on bulk and supported Cu catalysts on ZnO, SiO_2_, MgO, and Al_2_O_3_ for the production of 1,2-PG in both the liquid and vapor phase [29]. Recently, Jimenez et al. published a technical and economic evaluation of a process for the production of 1,2-PG from glycerol, exploring it as an alternative to the use of H_2_ that was obtained locally from glycerol reforming. Their results showed that the process is economically feasible using commercially available H_2_ [39].

The present review paper proposes to critically discuss the articles reported in the literature on the catalytic hydrogenolysis of glycerol for the production of 1,2-PG. For this purpose, more than 300 articles were analyzed and reviewed, covering the literature from about 20 years, from 2003 up to 2023. The article focuses on the heterogeneous catalysts based on noble (Ru, Pt, Pd) and non-noble (Cu, Ni, Co) active phases, supported and not-supported ones, used both in the liquid and vapor phase. Aspects related to the reaction mechanisms and the effect of the operating variables on the batch and flow continuous reactors are extensively discussed. Then, the main deactivation mechanisms associated with catalytic systems, such as coking, sintering, leaching, and the presence of impurities in the glycerol feed are discussed. Finally, the kinetic models used for the design of hydrogenolysis reactors are presented. It is intended that this review will serve as a key to open up new active, selective, stable materials for the production of 1,2-PG from glycerol.

## 2. Reaction Mechanisms

**Scheme 4:**
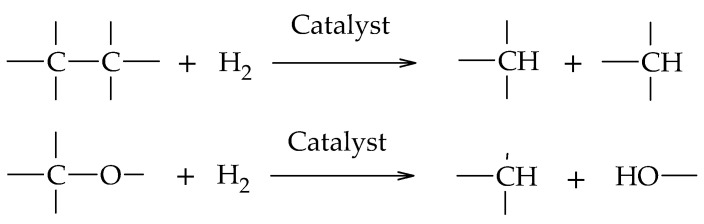
C-C and C-O bond cleavage in the hydrogenolysis reaction.

Glycerol hydrogenolysis is an organic reduction reaction where C-O and C-C bond cleavage occurs by H_2_ in the presence of catalysts (Scheme 4). The cleavage of C-O bonds leads to a decrease in the oxygen content of the reactive molecule, maintaining its total number of carbon atoms, while the C-C cleavage decreases the original length of the reactive molecule, fragmenting it into smaller molecules.

**Scheme 5:**
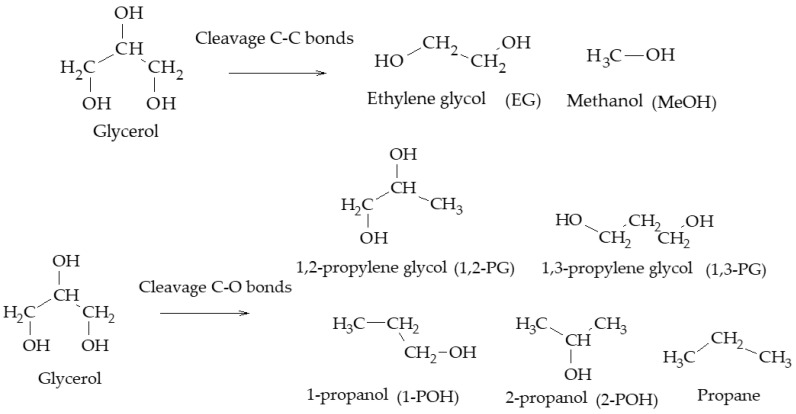
Main products of glycerol hydrogenolysis from C-O and C-C cleavage.

In particular, the cleavage of C-O bonds in the hydrogenolysis of glycerol can lead to the formation of 1,2-PG, 1,3-PG, 1-POH, 2-POH, and propane, while the cleavage of C-C bonds leads to the formation of EG and MeOH (Scheme 5) [40].

The generation of one product or another depends on the operating conditions and the catalysts used in the hydrogenolysis reaction. The hydrogenolysis mechanisms focused on the production of 1,2-PG are presented below, in order of chronological appearance.

### 2.1. Dehydrogenation-Dehydration-Hydrogenation Mechanism

**Scheme 6:**
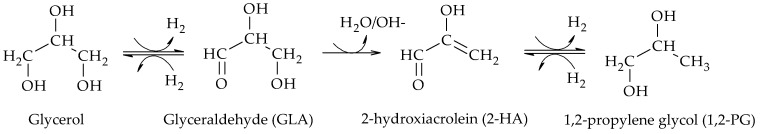
Dehydrogenation–dehydration–hydrogenation mechanism to form 1,2-PG.

The first mechanistic reports were provided by Montassier et al., who studied the hydrogenolysis of glycerol under very dilute conditions (1–4 wt.%), using Ru/C as catalyst, at moderate temperatures (180–260 °C) and high pressures (3–4 MPa) [41]. The presence of glyceraldehyde (GLA), 2-hydroxyacrolein (2-HA) and 1,2-PG among the reaction products suggested that the reaction proceeded via dehydrogenation of glycerol over the metal sites of the catalyst to produce GLA, followed by dehydration in basic medium of GLA to produce 2-HA and finally hydrogenation of 2-HA to produce 1,2-PG over the metal sites of the catalyst (Scheme 6).

Due to the presence of H_2_, the dehydrogenation step of glycerol to GLA is thermodynamically limited, especially at high operating pressures. In these cases, GLA can be rapidly hydrogenated back to glycerol. Thus, in order to favor the formation of 1,2-PG, the dehydration step should be as fast as possible, a situation that is achieved in the presence of either a basic medium or catalysts with suitable acid-base properties.

**Scheme 7:**
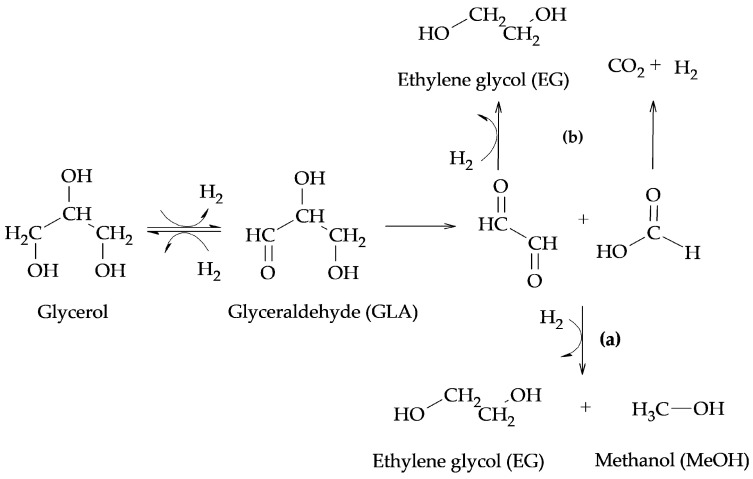
Formation of EG, MeOH, and CO_2_: (**a**) retro-aldol reaction (**b**) retro-Claisen reaction.

The above-mentioned authors proposed two different routes for the formation of products from C-C bond cleavage reactions such as EG, MeOH, and CO_2_. In the presence of Ru catalysts, they proposed the formation of EG and MeOH from the retro-aldol reaction indicated in route (a) of Scheme 7, while, in the presence of Cu catalysts, they proposed that EG can be formed from a retro-Claisen reaction, as indicated in route (b) of Scheme 7.

The results obtained by Montassier et al. demonstrated that the selective hydrogenolysis of glycerol to 1,2-PG should occur in the presence of bifunctional catalysts with the ability to both dehydrate and hydrogenate, but also revealed that they could be influenced by the presence of bases such as hydroxyls. In this regard, Lahr et al. reported the effect of the pH of the reaction medium on the hydrogenolysis of glycerol employing a Ru/C catalyst. The authors showed that the addition of bases increases the reaction rate for the formation of 1,2-PG and EG in different proportions depending on the pH value of the medium. At pH = 8, the production of EG is increased, whereas at pH = 11 the formation of EG is disfavored [42].

**Scheme 8:**
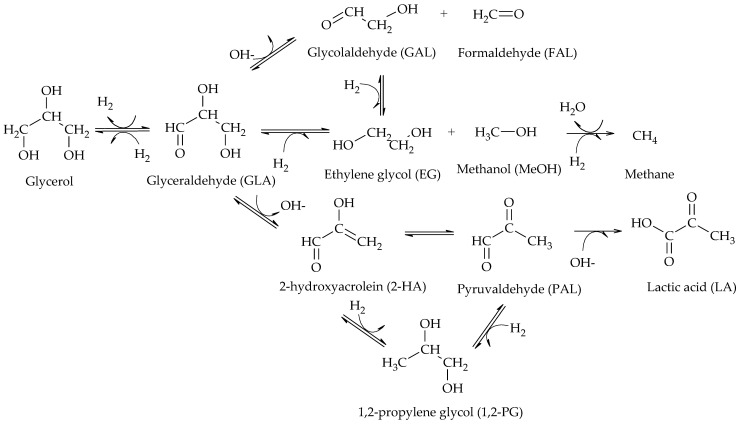
Reaction mechanism proposed by Maris et al. [43].

Maris et al. studied the effect of the addition of bases, NaOH and CaO, using 1 wt.% aqueous solutions of glycerol, at 200 °C and 40 bar of H_2_, with commercial Ru/C and Pt/C catalysts [43]. At neutral pH, Ru/C was the most active and selective towards glycol formation; however, it favored EG production over 1,2-PG and also catalyzed methane formation. Although less active, Pt/C allowed 1,2-PG to be obtained with high selectivity. The presence of both NaOH and CaO enhanced the reactivity of Pt to a greater extent than that of Ru, but lactic acid formation was significant at high pH. Based on their results, these authors proposed a reaction mechanism shown in Scheme 8. There, the first step is the dehydrogenation of glycerol to GLA catalyzed by metal sites and can be enhanced by the presence of a base. Then, GLA dehydrogenates to 2-hydroxyacrolein (2-HA), which is hydrogenated to form 1,2-PG. Two different routes for the formation of EG from GLA are also proposed. In one of them, GLA dehydrates to glycolaldehyde (GAL) which eventually hydrogenates to form EG. In the other, GLA hydrogenates directly and by C-C cleavage forms EG and MeOH. The authors also observed the presence of lactic acid (LA) at high pH values, which would be produced from pyruvaldehyde (PAL) by dehydration of GLA.

**Scheme 9:**
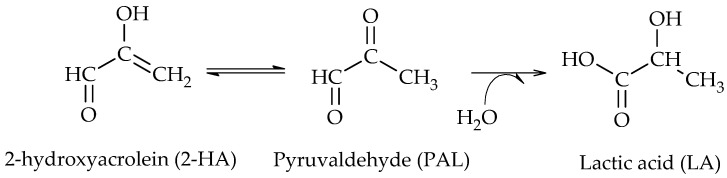
Formation of lactic acid (LA) from pyruvaldehyde (PAL), according to Feng et al. [44].

Following this study, Feng et al. studied the effect of adding different inorganic bases such as LiOH, NaOH, KOH, Na_2_CO_3_, K_2_CO_3_, and Li_2_CO_3_ to the reaction medium in the presence of a Ru/TiO_2_ catalyst. The authors demonstrated that the addition of bases favors the dehydrogenation of glycerol to GLA and promotes the dehydrogenation of GLA to 2-hydroxyacrolein (2-HA), which is in agreement with the proposal of Maris et al. They also indicated that an oxidation product such as lactic acid (LA) can be formed even under a reducing atmosphere. The results obtained by DFT show that LA can be easily obtained by a Cannizzaro reaction from pyruvaldehyde (PAL), where the latter comes from the keto-enolic equilibrium with 2-hydroxyacrolein (2-HA) (Scheme 9) [44].

### 2.2. Dehydration–Hydrogenation Mechanism

**Scheme 10:**
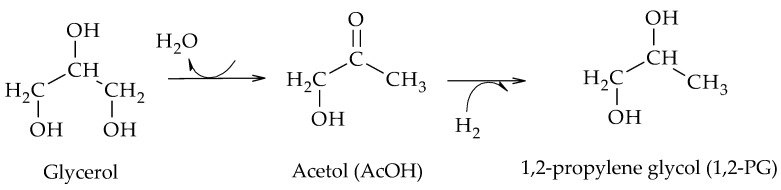
Dehydration–hydrogenation mechanism for the formation of 1,2-PG, according to Dasari et al. [45].

Then, 17 years after the first results obtained by Montassier, Dasari et al. used a Cu/Cr2O4 catalyst to carry out the hydrogenolysis of glycerol in a liquid phase batch reactor at 200 °C and 1.4 MPa of H_2_, using 80 wt.% analytical glycerol solutions and a reaction time of 24 h. Their results evidenced the formation of 1,2-PG from acetol (AcOH) as an intermediate [45]. According to the mechanism proposed by the authors, glycerol dehydrates to AcOH which then leads to the formation of 1,2-PG by hydrogenation (Scheme 10).

The results obtained by Dasari et al. allowed the study of other aspects of the reaction, such as the addition of bases and acids and their effects on the reaction medium, although they did not include the formation of other hydrogenolysis compounds such as 1,3-PG, 1-POH, and 2-POH [44].

It has been reported that the formation of 1,2-PG from AcOH is a reversible process, with the direct reaction (AcOH → 1,2-PG) being much faster than the reverse reaction (1,2-PG → AcOH), which favors the formation of 1,2-PG from AcOH in the presence of H_2_ [46]. In this regard, using in situ IR spectroscopy, Yfanti et al. were able to prove that the formation of 1,2-PG from glycerol occurs via AcOH, and is favored by the availability of H_2_ in the reaction medium [47].

**Scheme 11:**
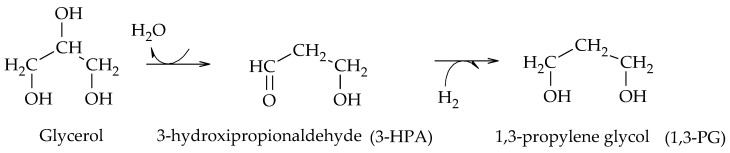
Dehydration–hydrogenation mechanism for the formation of 1,3-PG, according to Miyazawa et al. [48].

Miyazawa et al. found that the use of acidic materials, such as H_2_SO_4_, HCl and commercial resins, promotes the dehydration of glycerol to AcOH, and that the metal sites of supported catalysts (Ru, Pt, Rh, Pd) lead to the formation of 1,2-PG via the hydrogenation of AcOH. Moreover, in their results, they proposed the first route to obtain 1,3-PG by analogy to that of 1,2-PG. According to the authors, in the first instance glycerol is dehydrated to 3-hydroxypropionaldehyde (3-HPA), which is then hydrogenated to generate 1,3-PG (Scheme 11) [48].

**Scheme 12:**
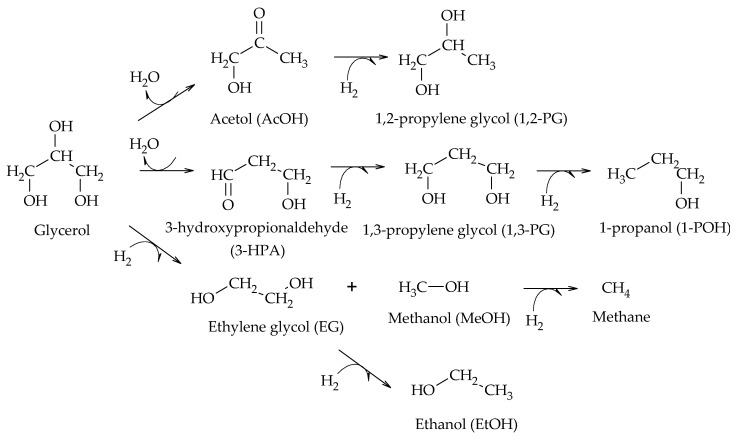
Dehydration–hydrogenation mechanism for the formation of 1-POH, EG, MeOH, and EtOH, according to Miyazawa et al. [48].

Miyazawa et al. also proposed the formation of 1-POH by the consecutive hydrogenolysis of 1,3-PG, and the formation of EG and MeOH from glycerol, with the subsequent hydrogenation of EG to produce EtOH (Scheme 12) [48].

For several of the reactions involved in this mechanism, Maglinao et al. determined vapor phase equilibrium constants (Kp), reporting that both glycerol dehydration to AcOH (Kp (190 °C) ≅ 7.3 × 10^9^) and glycerol dehydration to 3-HPA (Kp (190 °C) ≅ 8.2 × 10^7^) are thermodynamically favored [46]. The values of both constants would confirm that AcOH formation is more favorable than 3-HPA formation, which is consistent with the fact that 1,2-PG formation predominates over 1,3-PG formation [49,50]. With respect to the hydrogenation reactions, however, they found that both the hydrogenation of AcOH at 1,2-PG (Kp (190 °C) ≅ 10^−1^) and the hydrogenation of 3-HPA at 1,3-PG (Kp (190 °C) ≅ 2.6 × 10^−2^) are limited by thermodynamic equilibrium [46].

**Scheme 13:**
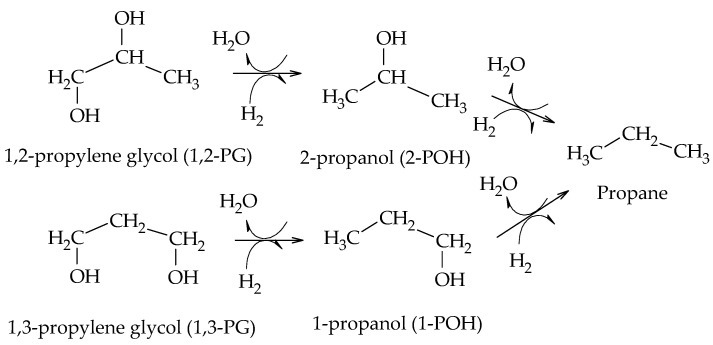
Formation of 1-POH, 2-POH, and propane from glycols, according to Perosa et al. [51].

Perosa et al. reported the formation of 1-POH and 2-POH from 1,2-PG and 1,3-PG using a Ni-Raney catalyst. They also detected the presence of propane from the hydrogenation of 1-POH and 2-POH (Scheme 13) [51].

**Scheme 14:**
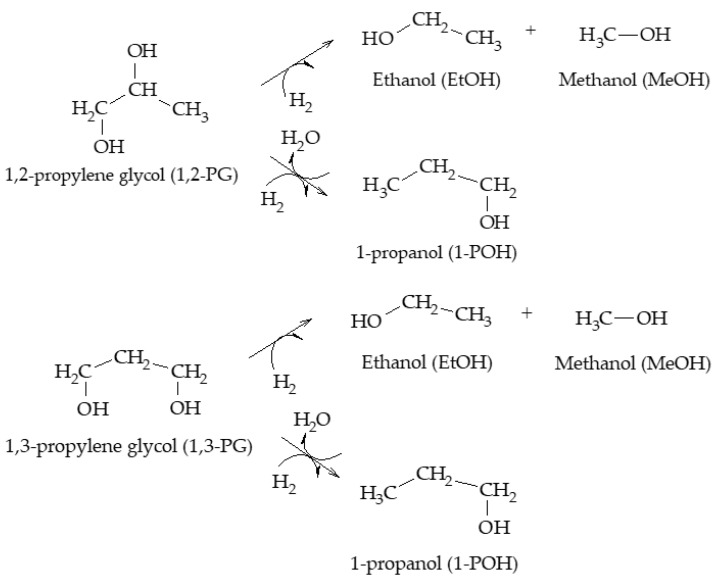
Formation of 1-POH, EtOH, and MeOH from glycols, according to Kunosoki et al. [53].

Regarding the formation of propanols, Furikado et al. demonstrated that by employing Rh/C catalysts, 2-POH is formed from 1,2-PG, whereas by employing Ru/C catalysts only 1-POH is present, which would be formed at the expense of 1,2-PG [52]. Kunosoki et al. established that 1-POH could be formed from both 1,2-PG and 1,3-PG following a hydrogenation step and suggested that both glycols could lead to the formation of MeOH and EtOH (Scheme 14) [53].

Regarding the formation of other minority compounds present in this mechanism, Magliano et al. indicated that the hydrogenolysis of either 1,2-PG or AcOH can lead to the formation of EtOH; however, formation via 1,2-PG thermodynamically predominates over formation via AcOH [46].

### 2.3. Combined Mechanisms

Hybrid mechanisms have been also reported, which combine some reactions of the dehydration–hydrogenation mechanism with part of the reactions of the dehydrogenation–dehydration–hydrogenation mechanism, existing as combined mechanisms.

**Scheme 15:**
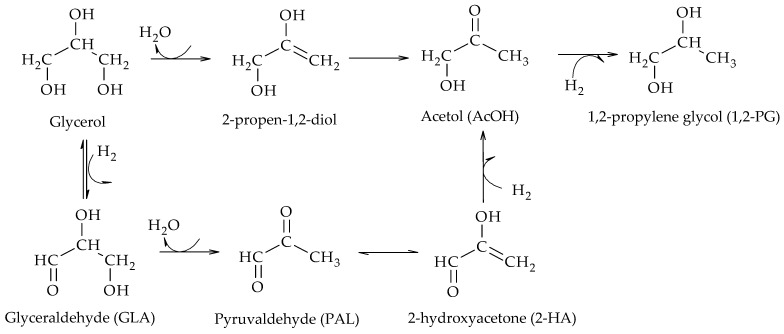
Combined reaction mechanism proposed by Wu et al. [54].

Wu et al. proposed two possible pathways for the formation of AcOH: the traditional one from the dehydration of glycerol over the support sites and another one from the dehydrogenation of glycerol to glyceraldehyde (GLA) over metal sites, followed by the dehydration of GLA to pyruvaldehyde (PAL), and finally the hydrogenation of PAL to form AcOH (Scheme 15) [54]. The authors also proposed the formation of 2-propen-1,2-diol, prior to the formation of AcOH, using a Cu/Bohemite catalyst.

In this regard, other articles [49,54,55,56,57] have also reported this intermediary in the formation of AcOH. Kongpatpanich et al. performed DFT calculations for an H-ZSM-5 type zeolite and found that AcOH formation proceeds via two steps, first the dehydration of glycerol to 2-propen-1,2-diol and then a tautomerization of the enolic form to form AcOH [55]. This species was also identified by Auneau et al. with a Rh/C catalyst [56]. Guan et al. concluded that AcOH formation proceeds via 2-propen-1,2-diol with Cu/MgO and Cu/ZrO_2_ catalysts [49]. Vila et al. investigated hydrogenolysis using glycerol and deuterated water in the presence of Cu/Al_2_O_3_ catalysts. By DRIFT, they identified the formation of isomers, 1,2-enediol, 2,3-enediol, and 2-hydroxypropanal, which are in tautomeric equilibrium with AcOH. The identified species were followed during the reduction process employing the nuclear magnetic resonance technique (1H-NMR) and the results showed that the formation of 1,2-PG proceeds mainly via 2,3-enediol (50%), followed by 2-hydroxypropanal (40%), and finally by AcOH (10%) [57].

### 2.4. Chelation-Hydrogenation Mechanism

**Scheme 16:**
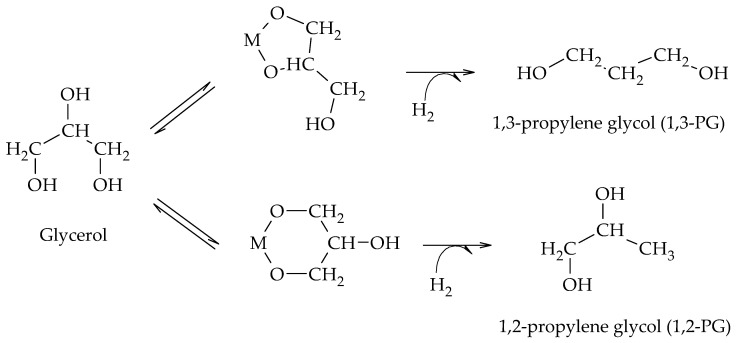
Chelation–hydrogenation mechanism for the formation of 1,2-PG and 1,3-PG.

Chaminand et al. proposed a mechanism consisting of a chelation step of glycerol on the metal sites and subsequent hydrogenation in the presence of H_2_ to form alternatively 1,2-PG or 1,3-PG (Scheme 16) [58]. The hydrogenolysis was carried out at 180 °C and 8 MPa of H_2_ using Rh, Pd, and Cu catalysts supported on ZnO, C, and Al_2_O_3_. Of all the catalysts studied, Rh/C + H_2_WO_4_ allowed the glycols to be obtained. Replacing part of the water by other solvents, such as sulfolane and dioxane, made the reaction selective to each of the glycols and increased the level of activity.

The chelation of glycerol on the metal sites of the catalyst (M) and its subsequent hydrogenation suggest that the formation of 1,3-PG is favored over the formation of 1,2-PG due to the stability of the intermediate formed. However, this was the only work that proposed chelation as an intermediate process in the formation of 1,2-PG and 1,3-PG.

### 2.5. Direct Mechanism of Hydrogenolysis

**Scheme 17:**
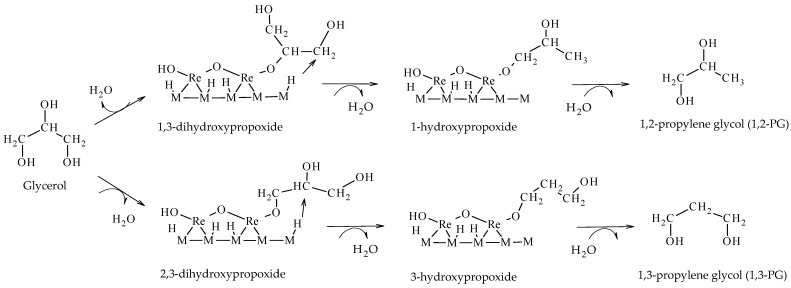
Direct mechanism of hydrogenolysis over M-ReOx (M = Ir, Rh) catalysts.

Tomishige et al. reported the hydrogenolysis of glycerol solutions (20–100 wt.%) in a liquid phase at 100–180 °C and 2–8 MPa of H_2_, employing Ir-MOx/SiO_2_ and Rh-MOx/SiO_2_ (M = Re, Mo, V) catalysts. Taking Ir-ReOx/SiO_2_ and Rh-ReOx/SiO_2_ as the reference, they formulated a direct mechanism of hydrogenolysis based on the formation of hydroxypropoxides leading to the formation of glycols (1,2-PG or 1,3-PG), depending on the intermediate formed (Scheme 17). In this mechanism, glycerol is adsorbed on ReOx species forming 1,3-dihydroxypropoxide or 2,3-dihydroxypropoxide. Then, H_2_ adsorbs dissociatively on the metal sites (Ir or Rh) and attacks the tertiary or secondary carbon of the intermediate species forming 1-hydroxypropoxide or 3-hydroxypropoxide, respectively. Finally, these species are hydrolyzed to form 1,2-PG or 1,3-PG [59,60,61].

Guan et al. employed DFT calculations and concluded that the formation of 1,3-PG occurs via a direct reaction mechanism via alkoxide species forming on ReOx particles on Ir-ReOx/ZrO catalysts. Their results showed that the formation of terminal alkoxides (2,3-dihydroxypropoxide) prevails over the formation of secondary alkoxides (1,3-dihydroxypropoxide), indicating that the formation of 1,3-PG is favored over the formation of 1,2-PG in these catalytic systems [62]. These results are in agreement with the results reported by Tomishige’s group employing Ir-Re/SiO2 catalysts [59,60,61,62].

**Scheme 18:**
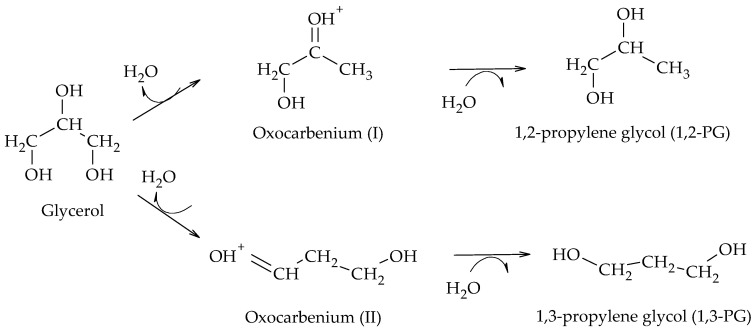
Direct mechanism of hydrogenolysis over Pt/WOx/S catalysts.

Another direct mechanism of hydrogenolysis was proposed for Pt/WOx/S-type catalysts, S being ZrO_2_ [63], WOx [64], AlOOH [65], Al_2_O_3_ [66,67], and SBA-15 [68] supports. Employing these catalysts, it has been proposed that glycol formation occurs by a mechanism involving proton and hydride transfer at the catalytic surface [63]. Although the mechanism presents variations depending on the support employed, the formation of 1,2-PG or 1,3-PG starts with the adsorption of glycerol on the support and the dissociative adsorption of H_2_ through a heterolytic cleavage into H^+^ and H^−^ on the metal sites. Then, the adsorbed glycerol is dehydrated to form a primary (I) or secondary (II) oxocarbenium type intermediate, depending on the attack of the H^+^ on the primary or secondary -OH group of the glycerol. Finally, the H- ions are transferred to the oxocarbenium (I) or (II) ions to form 1,2-PG or 1,3-PG, respectively (Scheme 18). It has been shown by theoretical DFT calculations that the oxocarbenium intermediates are relatively stable and that the formation of other enol-type intermediates from them is unlikely [69].

### 2.6. Etherification-Hydrogenation Mechanism

**Scheme 19:**
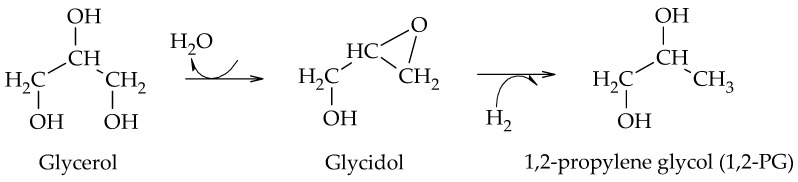
Etherification–hydrogenation mechanism to obtain 1,2-PG.

Wang et al. proposed the formation of 1,2-PG from glycidol using Cu-ZnO catalysts, prepared by the coprecipitation method, at 180–240 °C and 4.2 MPa of H_2_. Their results showed that hydrogenolysis proceeds via the dehydration of glycerol to glycidol on the surface of ZnO particles and then hydrogenates on Cu particles. Although no other work had reported the existence of glycidol as an intermediate compound, the authors proposed a new pathway for the formation of 1,2-PG from glycerol (Scheme 19) [70].

**Scheme 20:**
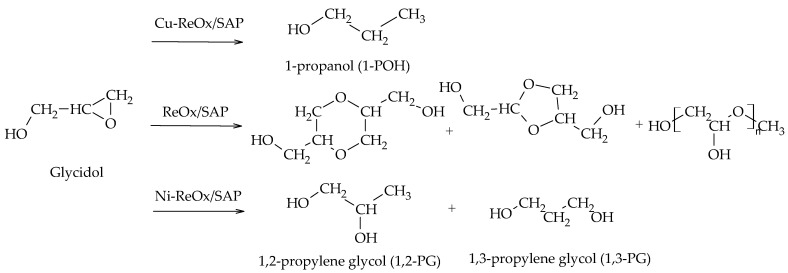
Etherification–hydrogenation mechanism via glycidol.

Recently, Gebretsadik et al. completed the etherification–hydrogenation mechanism by incorporating the formation of 1,3-PG. The authors employed Ni, Cu, and Ni-Cu catalysts supported on a saponite and modified with transition metal oxides (V, Mo, W, Re). Their results showed that Ni-ReOx mainly favors the formation of 1,3-PG, while Cu-ReOx allows for obtaining 1-POH via deoxygenation–hydrogenation reactions. Moreover, the ReOx-modified saponite support favors the formation of condensation products (Scheme 20) [71].

The analysis of the reaction mechanisms indicate that the formation of 1,2-PG depends on the acidity or basicity of the reaction medium and the catalyst employed. Acidic media favor the formation of 1,2-PG by the dehydration–hydrogenation mechanism, with AcOH as an intermediate product and the possible formation of EG, MeOH, 1-POH, 2-POH, EtOH, and gases. Basic media, on the other hand, promote the formation of 1,2-PG by the dehydrogenation–dehydration–hydrogenation mechanism, with the presence of GLA, 2-HA, and PAL as intermediates and AL as a by-product, among others. In addition, the employment of a certain group of catalysts, based on Ir, Rh, and Pt, in the presence of MOx (M = Re, V, Mo, W), leads to the 1,2-PG formation and its isomer through a direct hydrogenolysis mechanism, including the formation of intermediates on the catalytic surface.

## 3. Catalysts for 1,2-PG Production

The production of 1,2-PG has been extensively studied in the literature. Bulk and supported catalysts based on different metal phases have been used, with the particle size and the surface acidity being the relevant catalytic properties in the production of this compound.

Regarding the bulk catalysts, Cu, Ni, and Co have been used due to their low cost compared to noble metals. Of all of them, Cu-based bulk catalysts were the first to be studied due to the ability of Cu to cleave C-O bonds. Within the set of supported catalysts, Ru, Pt, and Pd metal phases have been used, in addition to the previously mentioned non-noble metals.

Traditional supports based on metallic and non-metallic oxides as well as carbonaceous supports have been studied under the reaction conditions. Regardless of the metal phase, the acid-base properties of the supports are key to obtain high selectivity values at 1,2-PG.

The most relevant aspects in the study of catalysts used in the hydrogenolysis reaction of glycerol to 1,2-PG are described below, differentiating according to the metal phase and the type of reactor used, both in liquid and vapor phase.

### 3.1. Ru Catalysts

Of the set of supported metal catalysts for the production of 1,2-PG, those based on Ru as the active phase have been the most studied ones due to the high intrinsic activity of the metal. Table 1 summarizes the operating conditions and best activity results of supported Ru catalysts in batch reactors. In all cases, the maximum performance achieved at 1,2-PG is reported.

Dasari et al. were the first authors to evaluate commercial Ru/C and Ru/Al_2_O_3_ catalysts using 80% aqueous glycerol solutions. The results showed that both catalysts were active, but not as selective to 1,2-PG as the Cu catalysts (~40% for Ru/C and ~60% for Ru/Al_2_O_3_). The authors assigned these results to the lower ability of Ru to hydrogenate C-O bonds compared to the Cu metal phase [45]. Following these results, Maris et al. studied commercial Ru/C catalysts and concluded that Ru promotes C-C bond cleavage reactions, favoring the formation of side products such as EG and EtOH [43].

With respect to other noble metals (Pt, Pd, Rh), Ru has been shown to have a higher intrinsic activity in the hydrogenolysis of glycerol. Maris et al. compared the activity of Ru/C with the activity of Pt/C and concluded that the activity of the former is higher than that of the latter under the same operating conditions [43]. Alhanash et al. evaluated Ru and Rh catalysts supported on a heteropolyacid salt (CsPW). Ru/CsPW proved to be more active than Rh/CsPW, allowing to obtain high selectivity to 1,2-PG (96%) with glycerol conversions of 20%, employing low hydrogen pressure (0.5 MPa) [79]. Wang et al. demonstrated that the Ru/ZrO_2_ catalyst was the most active one compared to Rh, Pt, and Pd catalysts. The order of activity, for particle sizes of the order of 2 nm, was as follows: Ru > Rh > Pt > Pd [87].

Some articles reported that the activity of Ru-supported catalysts is affected by the metal particle size and the acidity of the catalyst. With respect to particle size, it has been reported that small particle sizes result in high dispersions that increase conversion levels. In addition, it has been reported that the particle size depends on the type of the Ru precursor, the preparation method, and the support. On the other hand, the surface properties of the support have the greatest impact on the acidity of the catalyst.

The most commonly employed Ru precursors have been RuCl_3_ [48,53,72,73,74,75,76,77,79,80,81,85,86,87,88,89,90,91,92], Ru(NO)(NO_3_)_3_ [48,77,84,86,91], Ru (AcAc)_3_ [48,82], and Ru_3_(CO)_12_ [82].

Several studies have reported that of all the precursors, Ru(NO)(NO_3_)_3_ allows for obtaining the highest glycerol conversion and selectivity to 1,2-PG [48,77,86,91,92,93] because its decomposition generates small Ru particles with sizes smaller than 2 nm [48]. A similar effect was reported employing RuCl_3_, which has been one of the most widely used precursors [93]. However, some articles have highlighted that the presence of Cl^−^ ions generate acidic Bronsted sites on the surface of the supports [72] that would be responsible for promoting the formation of 1-POH, 2-POH [77], EG, and MeOH [86]. Other precursors such as Ru(AcAc)_3_ and Ru_3_(CO)_12_ have been less employed and in comparison to the other precursors have shown lower levels of activity [48,82]. Based on these results, the following order of activity per precursor type could be established:

Ru(NO)(NO_3_)_3_ > RuCl_3_ > Ru_3_(CO)_12_ > Ru(AcAc)_3_

With respect to the preparation methods, the most commonly employed have been the wetness incipient impregnation [43,48,72,73,75,77,79,80,81,82,84,85,86,87,88,90,93] and deposition–precipitation [76,90]. Balaraju et al. prepared Ru/TiO_2_ catalysts employing both methods. Their results showed that the deposition–precipitation method allowed higher metal dispersion (29–53%) to be achieved, due to the reduction in Ru(OH)_3_ species generated during the catalyst preparation process, which led to obtaining small size metal particles (dva ~ 1–3 nm). With the use of the incipient moisture impregnation method, the formation of larger Ru particles (dva ~ 10 nm) led to lower activity levels [90].

Catalyst activation has also been indicated as a variable to consider in metal particle size. For Ru/C [48] and Ru/TiO_2_ [81] catalysts, it has been reported that high activation temperatures lead to the agglomeration of Ru particles, and this causes a drop in conversion levels. For Ru/AC catalysts, the reduction in the H_2_ atmosphere was found to be less effective than the reduction in the presence of NaBH_4_ in the liquid phase due to the larger Ru particle sizes obtained (3–5 nm) [86].

The support also impacts on the activity levels, not only because of its effect on Ru particle size but also because of its acid-base properties. Reports have indicated the use of carbonaceous supports in the form of carbon graphite (C), activated carbons (AC), carbon nanotubes (CNT), and high specific surface-area graphite (HSAG), in addition to oxides such as Al_2_O_3_, SiO_2_, ZrO_2_, and TiO_2_, and zeolites of the Hβ and HZSM-5 type. Within this set, carbonaceous supports have been the most studied ones, because they provide the necessary specific surface area for the formation of Ru particles with high dispersion [72,78,92] and can be functionalized to increase their acidity [91].

With respect to the effect of the particle size, activated carbons (AC) have proved to be better supports than graphitic carbons (C) with a different degree of crystallinity, because they led to a higher dispersion of Ru. Mane et al. indicated that Ru/AC was the most active and selective catalyst to 1,2-PG due to the formation of Ru particles with sizes between 1.1 and 1.7 nm. The use of Ru/C catalysts with Ru particles of larger size, between 4 and 5 nm, and with the presence of Ru oxides, presented lower levels of activity [92]. On the other hand, Ru catalysts supported on CNT and HSAG proved to be more active than Ru/AC catalysts under the same reaction conditions, due to the formation of a higher amount of electron-rich Ru^δ−^ species that favored the cleavage of the terminal C-O bond of glycerol to form 1,2-PG [72].

With respect to acidity, the carbonaceous supports would have the necessary acidic properties in the hydrogenolysis reaction. However, a specific type of acid site is required, and the number of sites must be appropriate to avoid losses in selectivity to 1,2-PG. Gallegos-Suarez et al. studied Ru/AC catalysts modified by functionalization with nitric acid. The results showed that -COOH groups, generated during carbon oxidation, favor the cleavage of C-O bonds. In addition, the study showed that, although the activity was promoted, the selectivity to 1,2-PG decreased compared to catalysts supported on unfunctionalized AC. The results were attributed to the high level of acidity provided by the functionalization process which resulted in the formation of side products such as 1-POH, 2-POH, MeOH and other gases such as methane, ethane, and propane [91].

Some traditional oxides such as Al_2_O_3_, SiO_2_, ZrO_2_, TiO_2_, and Hβ-type and HZSM-5 zeolites were also employed as supports, but the activity of Ru on these supports was lower than the results obtained employing carbonaceous ones. Ma et al. reported the use of Al_2_O_3_ and ZrO_2_ as supports [88], as well as SiO_2_, TiO_2_, and those of the Hβ and HZSM-5 type [74]. The order of activity (based on the yield to 1,2-PG) was as follows:

Ru/C > Ru/Hβ > Ru/H-ZSM5 > Ru/ZrO_2_ > > Ru/SiO_2_ ~ Ru/Al_2_O_3_ > Ru/TiO_2_

The authors found that the Ru particle size is smaller for the Ru/C catalyst, followed by the Ru/ZrO_2_ and Ru/SiO_2_ catalysts, suggesting that the activity found is due to the higher dispersion obtained on the supports [88]. However, the activity for the zeolite-based catalysts also showed good activity, suggesting that the role of acidity is not less important in such materials.

Other less-studied supports were hydrotalcites. Lee et al. prepared Ru catalysts supported on a CaMgZn-Al type hydrotalcite. The results showed high levels of conversion and selectivity to 1,2-PG due to the higher acidity of the hydrotalcite with respect to other supports, such as γ-Al_2_O_3_, and the smaller metal particle size (d_va_ ~ 13.7 nm) [84].

With the aim of improving the surface textural and acidic properties, the modification of some of the traditional oxides used as supports has been studied.

For the ZrO_2_ support, the addition of WO_3_ led to a decrease in Ru/ZrO_2_ catalyst activity, but an improvement in selectivity to 1,2-PG, due to the suppression of C-C bond cleavage reactions [73]. The addition of La to the same support, on the other hand, caused a decrease in selectivity to 1,2-PG due to the promotion of C-C bond cleavage reactions, that increased the selectivity to EG [76].

For the TiO_2_ support, it has been demonstrated that it is possible to obtain good metallic dispersions by modification with clays. Hamzah et al. employed a TiO_2_: bentonite support (with a 1:2 mass ratio) and obtained Ru particles of the order of 1.5 nm that allowed the achievement of a high glycerol conversion (~70%) and selectivity to 1,2-PG (~80%) [85].

With respect to Ru/Al_2_O_3_ catalysts, the modification of the support with AlF_3_ for contents between 17 and 58 wt.% allowed the maximum glycerol conversions to be reached, due to a higher dispersion of Ru particles; however, it also promoted the formation of EG and gases such as methane [75], probably due to the surface acidity generated by the presence of the modifier.

Other more complex catalytic systems, such as Ru/LaCO_3_OH, were found to be more active, selective, and stable than the Ru/SiO_2_ and Ru/ZrO_2_ catalysts. Ru/LaCO_3_OH was prepared from the precursor RuCl_3_, which reacts during impregnation with the support to form a complex of LaRu(CO_3_)_2_Cl_2_ and LaOCl. During hydrothermal reduction, these intermediates hydrolyze to form LaCO_3_OH and Ru(OH)_3_, which subsequently transform into Ru0 nanoparticles. The improvement in activity levels is due to the presence of Ru0 encapsulated by a protective layer of LaCO_3_OH [80].

With the aim of increasing the glycerol conversion and improving the selectivity to 1,2-PG, bimetallic Ru catalysts have been studied employing several metals, such as Pt [94,95], Au [94], Re [74,88,96,97,98], Cu [99,100], Co [100,101,102], Ni [100] and Fe [103]. Table 2 summarizes the operating conditions and activity results of Ru bimetallic catalysts in batch reactors.

Some articles have reported that Ru–Pt and Ru–Au combinations did not produce improvements in the activity of monometallic Ru catalysts. The Ru–Pt/C combination, for example, did not generate an increase in the levels of conversion and selectivity to 1,2-PG with respect to a Ru/C catalyst, while the Ru–Au combination produced a higher selectivity to EG and lactate, decreasing the selectivity values to 1,2-PG. Moreover, the incorporation of Au favored the deactivation of the catalyst due to the sintering of the Ru particles [94].

On the other hand, the addition of Re and the formulation of Ru-Re bimetallic catalysts was favorable in all cases improving both conversion and selectivity to 1,2-PG. Early studies reported that the addition of a Re_2_(CO)_10_ precursor to the reaction medium markedly improved the activity and selectivity at 1,2-PG of the Ru/Al_2_O_3_ catalyst [88]. Further studies showed that the formulation of Ru-Re catalysts supported on SiO_2_, Al_2_O_3_, ZrO_2_, TiO_2_, and HZSM-5 and H-β type zeolites resulted in an improvement in activity levels, which was assigned to the dispersion of Ru particles by the addition of Re, suggesting a synergistic effect between the two species. The increase in selectivity to 1,2-PG was attributed to an inhibitory effect of Re on the formation of secondary products, such as 1-POH and 2-POH [74]. The existence of ReOx particles interacting with Ru^0^ particles has been found to be responsible for the enhancement of metal dispersion [96]. On the other hand, such species impart higher acidity to the catalyst [97]. Some reports have indicated that the enhancement in activity by Re also depends on the preparation method. Li et al. reported the preparation of Ru-Re bimetallic catalysts employing the deposition of a Ru-polyvinylpyrrolidone colloid followed by impregnation with the Re precursor. The results showed that, compared to the conventional impregnation method, this catalyst presented a better performance due to the smaller particle size and the high metal content that is possible to achieve with this preparation method [98].

The addition of other metals, such as Co and Ni, had a positive impact on Ru activity levels. Feng et al. studied the addition of these metals to Ru/TiO_2_ catalysts, showing that, although the conversion decreases, there is an increase in selectivity to 1,2-PG. The formulation of a Ru-Co catalyst proved to be the best, even using other supports such as SiO_2_, ZrO_2_, and Al_2_O_3_ [100,101,102]. Li et al. added Fe to a Ru/CNT catalyst, promoting an improvement in the activity and stability of the catalyst due to the formation of iron oxides at the periphery of the Ru particles, in the form of FeO and FeO_1+x_ (0 < x < 0.5). These species interact synergistically with the Ru particles up to a Ru:Fe molar ratio = 2. The authors showed that the addition of higher amounts of Fe causes a drop in activity levels due to the surface blocking of Ru [103].

The preparation of Ru-Cu bimetallic catalysts has also been reported in the literature. Liu et al. prepared catalysts supported on SiO_2_, TiO_2_, ZrO_2_, Al_2_O_3_, and HY and NaY zeolites. Of all the prepared catalysts, Ru-Cu/ZrO_2_ with a Cu:Ru molar ratio = 1:10 showed the best performance, due not only to the acidity of the support but also to the synergistic effect between Ru and Cu by electron transfer from Ru to Cu [106]. Salazar et al. studied Ru-Cu/TiO_2_ catalysts and found the maximum selectivity to 1.2-PG for a Cu:Ru mass ratio = 1:1, which was attributed to the interaction between Cu and Ru. The results indicated that the presence of Cu favors the formation of 1,2-PG by suppressing the formation of EG [105].

With respect to obtaining 1,2-PG in vapor phase, the results indicate that Ru catalysts do not selectively promote the formation of 1,2-PG because thermal levels increase the ability for C-C bond cleavage. Ru/SBA-15 catalysts showed yields for 1,2-PG of 15%, even though the glycerol conversion was 75% at 260 °C, 0.1 MPa of H_2_ and 2.21 h^−1^ (WHSV) [107]. Ru/γ-Al_2_O_3_ systems showed similar yields (17%) at 230 °C, 0.1 MPa of H_2_ and 2.09 h^−1^ (WHSV) [108].

From the results above, it can be concluded that Ru-based catalysts are active in glycerol hydrogenolysis to 1,2-PG, with the conversion depending on the metal particle size and the acidity of the support. Favoring metal dispersion and increasing support acidity, high conversions can be obtained, although those properties should be optimized so as not to generate side products. As monometallic Ru-catalysts are not so selective to 1,2-PG, bimetallic formulations are required, with Ru-Co, Ru-Fe, and Ru-Cu being the most effective ones. From all the formulations, Ru-Cu/Bentonite resulted in being the most active (yield to 1,2-PG = 86.4%).

### 3.2. Pt Catalysts

With respect to Pt catalysts, Table 3 summarizes the operating conditions and liquid-phase activity results employing batch reactors.

From the point of view of intrinsic metal activity, Pt catalysts have been demonstrated to be less active than Ru catalysts but have shown higher selectivity at 1,2-PG. Dasari et al. carried out the first studies on a screening of catalysts and determined that, comparatively, Pt/C is less active than Ru/C, but shows a higher selectivity to 1,2-PG [45]. In this regard, Maris et al. determined that the higher selectivity at 1,2-PG is attributed to the lower C-C bond cleavage ability of Pt compared to Ru [94].

With respect to other metal phases, such as Pd, Cu and Ni, Pt was found to be intrinsically more active. Von Held Soares et al. prepared Pt, Pd, and Ni catalysts supported on Fe_3_O_4_. The order of activity found was as follows: Pt > Pd > Ni. The results showed that, in the case of Pt, a spillover effect also contributes to improving the catalytic performance [117]. Recently, Wei et al. prepared catalysts of Pt, Cu, Ru, and other bimetallic combinations supported on tungsten carbides (WC_x_). The results showed that Pt/WC_x_ led to the highest yields for 1,2-PG [115].

Of the Pt catalyst set, early studies employed commercial Pt catalysts [45,94,112,113], but the activity of catalysts prepared from H_2_PtCl_6–_6H_2_O [94,110,111,114,117,119,120,121], Pt(NH_3_)_4_Cl_2_H_2_O [109,118], and (NH_4_)_2_PtCl_6_ [115] have also been evaluated. However, unlike Ru catalysts, papers have not reported studies on the particle size effect by the precursor type or preparation methods employed. Most of the studies have focused on the role of the support and its modification to achieve good metal dispersions and/or suitable surface acidic properties.

In this sense, the use of supports based on carbon ©, metal oxides such as MgO, Al_2_O_3_, SiO_2_-Al_2_O_3_, TiO_2_, ZnO, CeO_2_, and Fe_3_O_4_, zeolites of the H-ZSM5 and Hβ type and hydrotalcites (HTL) has been reported.

Within the group of carbonaceous supports, different types with varying specific surface areas (~250–1400 m^2^ g^−1^) have been employed. The Pt/C catalysts, prepared by vapor phase metal deposition on the supports, presented high metal dispersion with particle sizes between 1.3 nm and 2.5 nm, depending on the specific surface area of the support. These catalysts led to high levels of activity and stability [116].

With respect to supports based on metal oxides, zeolites, and hydrotalcites, the results are diverse and varied. Yuan et al. studied Pt catalysts prepared on acidic and alkaline supports. Their results showed that the more alkaline supports allow high conversions and selectivity to 1,2-PG to be obtained. The order of activity found was as follows: HTL > MgO > Al_2_O_3_ > Hβ > H-ZSM5 [109]. Other authors have found that acidic supports led to high levels of activity. Checa et al. studied Pt catalysts supported on Al_2_O_3_, CeO_2_, La_2_O_3_, and ZnO. The order of activity according to the support used was as follows: La_2_O_3_ > CeO_2_ > ZnO > Al_2_O_3_. The increase in activity was attributed to the increasing order of surface acidity of the supports [111]. Gandarias et al. reported that an amorphous SiO_2_-Al_2_O_3_ support allows the dehydration of glycerol to AcOH, due to its acidic properties [112]. It has also been reported that the acidic sites present in TiO_2_ supports are responsible for favoring dehydration reactions [110].

These results indicate that both alkaline and acidic supports allow the preparation of selective Pt catalysts towards the formation of 1,2-PG. According to the dehydrogenation–dehydration–hydrogenation mechanism, the alkaline supports would favor the dehydration of glyceraldehyde (GLA) to 2-hydroxyacrolein (2-HA) while the acidic supports, on the other hand, would favor the dehydration of glycerol to acetol (AcOH) in the dehydrogenation–hydrogenation mechanism.

In order to increase the selectivity towards 1,2-PG production, some authors have reported the modification of supports with acidic promoters. Rodrigues et al. studied Pt catalysts supported on Al_2_O_3_ modified with Nb_2_O_5_. The presence of Nb oxides, which endows the support with Bronsted-type acidity, favors the reduction in the Pt particles and enhances the cleavage of the C-O bond of the glycerol molecule [118].

Regarding the presence of acid sites in Pt catalysts, it has been reported that they should be as close as possible to the Pt atoms. In this regard, Du et al. modified a Pt/Al_2_O_3_ catalyst by the layered atomic deposition technique. Although there was no change in the total acidity of the catalyst, their results indicated that the acidic metal-site proximity enhances the bifunctional dehydration–hydrogenation mechanism [114].

To decrease the ability for C-C bond cleavage and increase the selectivity to 1,2-PG, bimetallic Pt catalysts were studied employing Re [122,123], Ni [120,121], Au [124], Sn [125], Fe [104], and Ir [126]. Table 4 summarizes the operating conditions and activity results of liquid phase Pt bimetallic catalysts employing batch reactors.

The addition of Re to Pt/C and Pt/CNT catalysts was effective for the production of 1,2-PG due to the formation of a Pt-Re alloy with sizes smaller than 2 nm [122,123]. The addition of Fe to Pt/γ-Al_2_O_3_ catalysts also promoted higher activity towards the formation of 1,2-PG due to the generation of new active sites involving both Pt and Fe atoms [104]. The Sn modification of Pt/SiO_2_ resulted in the formation of Sn^+n^ species with Lewis-type acidity, upon which the cleavage of the C-O bond of the glycerol molecule is facilitated, allowing high selectivity to 1,2-PG [125]. Recently, Liu et al. reported the modification of Pt/SiO_2_ with Ir-ReO_x_. The presence of ReO_x_ species favored C-O bond cleavage reactions, while Pt-Ir particles promoted H_2_ formation for hydrogenolysis but, at the same time, were active for C-C bond cleavage reactions [126].

The preparation of Pt-Ni catalysts on α-Al_2_O_3-_CeO_2_-ZrO_2_ [120] γ-Al_2_O_3_ [121] also allowed the performance of Pt catalysts to be improved due to the formation of a Pt-Ni alloy. For the case of Pt-Ni/α-Al_2_O_3_-CeO_2_-ZrO_2_, the species responsible for the increased activity was PtNi_3_ [120].

In contrast to Ru catalysts, the addition of Au to Pt catalysts improved the performance of the mono-metal catalysts. In this sense, bimetallic Pt-Au catalysts were prepared on MgO, SiO_2_, sulphated ZrO_2_, H-Mordenite, MCM-41, and TiO_2_. Of all the catalysts studied, Pt-Au/TiO_2_ was the most active one due to the higher dispersion of the Pt-Au particles (3.7 nm) [124].

From the foregoing, Pt-based catalysts are more active intrinsically than Ru-based catalysts, but show higher selectivity to 1,2-PG. The election of the support plays a fundamental role, as its acidity or basicity favors the dispersion of the metal particles and enhances the catalytic activity. Alkaline supports have been demonstrated to be the most suitable for glycerol hydrogenolysis, being the most active Pt/HTL among all the catalytic formulations (yield to 1,2-PG = 85.6%).

### 3.3. Pd Catalysts

Pd-supported catalysts have been less explored in the literature than Ru and Pt, and their use has been limited to the production of 1,2-PG in the liquid phase. Table 5 summarizes the operating conditions and activity results of liquid-phase Pd catalysts employing batch reactors.

Unlike Ru and Pt catalysts, Pd catalysts have been demonstrated to be more selective towards 1,2-PG formation due to their ability to cleave the C-O bond versus the C-C bond, although some were not very active. In this respect, Pd/SiO_2_ [127], Pd/C [45], Pd/ZrO_2_ [87], and Pd/bentonite [128] showed good selectivity to 1,2-PG (57–90%), but with low overall yields due to the low conversions obtained.

Most of the research work has focused on the appropriate preparation method to achieve high levels of conversion. Pd(NO_3_)_2_ [127], PdCl_2_ [87,119,130,131,132], Pd(AcO)_2_, and Na_2_PdCl_4_ [129] have been used as precursors for the preparation of Pd catalysts, although the use of commercial catalysts has also been reported [45,127].

With respect to the precursor used, the following order of activity has recently been reported: PdCl_2_ > Pd (NO_3_)_2_ ~ Pd(AcO)_2_ for Pd/SBA-15 catalysts [133]. It has been suggested that the presence of Cl^−^ from PdCl_2_ improves the reducibility of the catalyst, generating a higher metal dispersion, and therefore improving the activity levels [134].

Regarding the preparation, the co-precipitation method has been more efficient than the impregnation method, because it generates higher dispersions of the metal phase and therefore higher levels of activity [130,131]. In this sense, Pd/Fe_2_O_3_ [131], PdO/CoO [131], Pd/Co_3_O_4_ [130], and Pd/Fe_3_O_4_ [130] catalysts prepared by coprecipitation were more active than those prepared by impregnation.

The activation process has also been reported as a variable affecting activity. PdO/Fe_2_O_3_ [131] and PdO/CoO [131] catalysts showed 94% and 60% yields, respectively, under very similar reaction conditions, without being reduced in the H_2_ flow. In the case of PdO/Fe_2_O_3_ [132], its activity was higher than the activity of the reduced catalyst, Pd/Fe_2_O_3_ [131]. It even showed higher activity than the Pd/Fe_2_O_3_ catalyst prepared by deposition–precipitation, whose support was obtained by hydrothermal treatment and tested under more severe reaction conditions [129].

In order to improve activity levels, bimetallic Pd catalysts based on Pd-Re [133,135], Pd-Cu [136,137], Pd-Zn [138,139], and Pd-Ni [140] have been used. Table 6 summarizes the operating conditions and activity results of the bimetallic Pd catalysts in batch reactors.

Li et al. studied Pd-Re/SBA-15 catalysts and reported that the formation of ReOx particles increases the acidity of the catalyst and improves its reducibility, leading to improved activity levels [133]. In another article, the authors reported that the addition of Re decreases the average Pd size, and the oxidation state of the ReOx particles is much higher in the Pd-Re/SBA-15 catalyst than in the Re/SBA-15 catalyst [135].

Pd-Zn/ZrO_2_ systems have also been demonstrated to be active and selective due to the oxophilicity of Zn which promotes the cleavage of the α-C-H bond in the 2,3-hydroxypropanoxide intermediate on the surface of the Pd-Zn alloy. The results showed that this is the determining step in the reaction rate and therefore the performance of the Pd-Zn catalyst is much better than Pd [138]. The effect of Zn was also studied by Li et al., who found an improvement in activity by the formation of Pd-Zn nanoparticles [139].

Pdx-Cu_0_._4_/Mg_5_._6-x_Al_2_O_8_._6-x_ catalysts prepared by the co-precipitation method were found to be active and selective for the formation of 1,2-PG due to an enhanced ability to hydrogenate by a H_2_ spillover process from Pd to Cu [136]. The same effect was reported for Pd-CuCr_2_O_4_ catalysts. The presence of Pd allowed the reaction rate to increase about 1.7 times more than that obtained with CuCr_2_O_4_ catalysts. The H atoms stored in Pd diffused towards CuCr_2_O_4_ improving its reducibility and therefore increasing the number of surface-active sites formed from Cu^0^ [137].

According to the results, Pd-based catalysts are less active than Ru- and Pt-based catalysts, although they exhibit the highest selectivity to 1,2-PG due to the poor ability to cleave C-C bonds among the three noble metal phases. The preparation methods affect the catalytic performance of Pd-based catalysts due to the impact on metal dispersion. Although bimetallic formulations were implemented, the best results were obtained using PdO/Fe_2_O_3_ (yield to 1,2-PG = 94.0%).

### 3.4. Cu Catalysts

Within the group of catalysts based on non-noble metals (Cu, Ni, Co), the Cu catalysts, both supported and mass catalysts, have been the ones most widely used for the production of 1,2-PG, both in liquid and vapor phase.

Table 7 summarizes the operating conditions and activity results of the Cu bulk catalysts used in liquid phase in batch reactors.

Among the bulk catalysts, one of the first to be studied was Cu-Raney [41,127], obtained by leaching a Cu-Al alloy treated with NaOH. The reason for the high selectivity to 1,2-PG obtained with this catalyst (86%) is due to the intrinsic ability of Cu to cleave C-O bonds and the low ability to cleave C-C bonds, which decreases the selectivity to side products such as EG and gases [41]. The modification of Cu-Raney with metal oxides such as MgO allows for the improved performance at 1,2-PG due to the formation of a dispersed Cu-MgO phase in the Cu-Raney pores [149,164].

CuCr_2_O_4_ catalysts and their variants were also active in the hydrogenolysis reaction. In the case of CuCr_2_O_4_, it has been reported that the yield to 1,2-PG is a function of the degree of catalyst reduction, necessary to achieve the formation of active Cu^0^ and Cu^+^ species in the Cr_2_O_4_ matrix [45,154]. The existence of a CuCr_2_O_4_ spinel favored the hydrogenation step, due to the ability to accumulate both H_2_ and H bulk [150]. Recently, a theoretical study with DFT-based calculations revealed that the formation of AcOH is both thermodynamically and kinetically favored on the surface of the Cu and CuCr_2_O_4_ spinel. The spinel structure provides suitable sites for the adsorption of glycerol, its dehydration to AcOH, and subsequent hydrogenation to form 1,2-PG [165]. Similarly, it has been reported that another CuFe_2_O_4_-type spinel, present in Cu-Fe catalysts, was also active for the formation of 1,2-PG [145].

Other variants of the CuCr_2_O_4_ catalyst have been evaluated, which improved the yield to 1,2-PG under certain reaction conditions. The incorporation of Ba, for example, allowed stabilization and avoided the sintering of the Cu^0^ particles through the formation of BaCrO_4_ [142]. The preparation of a Cu-Cr system, through the sol–gel technique in the presence of propylene oxide, allowed an active catalyst to be obtained due to the presence of CuCr_2_O_4_, CuO, and Cr_2_O_3_ phases whose percentage content in the catalyst depends on the Cu:Cr molar ratio [146,166].

On the other hand, Cu-ZrO_2_ systems prepared by coprecipitation showed low yields for 1,2-PG (9%) [141]. Their modification with MgO and the optimization of the Cu:ZrO_2_ ratio allowed the yield to be improved to 1,2-PG (59%), due to the basic properties of MgO [109]. In fact, for Cu/MgO catalysts the activity was found to be a function of the basicity of the support, in addition to the dispersion of Cu and MgO particles formed during the catalyst preparation [153,167]. Recently, it has been reported that a Cu-MgO catalyst, with a Cu:MgO molar ratio = 0.5, led to 1,2-PG yields of 37% at 200 °C in the absence of H_2_ pressure, due to in situ H_2_ generation by glycerol reforming [168].

The replacement of MgO by SiO_2_ and ZnO were also tested alternatives in the preparation of Cu catalysts. CuO/SiO_2_ catalysts prepared by co-precipitation with colloidal SiO_2_ showed acceptable yields at 1,2-PG (69%) due to the presence of highly dispersed Cu^0^ particles, and Cu^+^ particles that enhanced the metal–support interaction, avoiding sintering [151,169].

With respect to the Cu-ZnO catalysts, the activity was found to be dependent on the Cu:Zn molar ratio, the preparation method, and the Cu^0^ particle size. The Cu:Zn molar ratio determines the formation of Cu and ZnO phases. In this regard, it has been reported that the formation of Cu and Zn hydrocarbonates with different crystalline phases, such as single-phase aurichalcite, favors the formation of highly interacting Cu with ZnO [152]. The coprecipitation method has been employed in the presence of urea [48,62] and oxalic acid [144] as precipitation agents, the latter being the best alternative due to the formation of CuO with higher surface area. The reduction in CuO generates small Cu^0^ particles that improve the activity and selectivity to 1,2-PG in both the urea [70] and oxalic acid [144] coprecipitation methods. Gao et al. used the previously reduced Cu-ZnO catalyst in a continuous flow reactor at 250 °C, 2 MPa of H_2_, and 7.6 h^−1^ (WHSV). The catalyst was stable for 200 h of reaction, with conversions of 93–96% and selectivity to 1,2-PG of 79–83% using crude glycerol and analytical grade glycerol, respectively. For a Cu:Zn molar ratio = 1.86, the maximum yield to 1,2-PG is achieved due to the maximum exposed Cu surface area [170].

More complex catalytic systems based on Cu, Zn and Al [127,156,160,171,172,173,174], Cu, Zn and Ga [159], Cu, Zn, Mg and Al [77,81,87,88] and Cu, Zn, Cr and Zr [162] showed higher yields for 1,2-PG than Cu/ZnO.

In Cu, Zn, and Al catalysts, the presence of Al_2_O_3_ provides a higher number of acid sites that favor the formation of 1,2-PG and modify the textural properties. In this sense, an improvement in the selectivity to 1,2-PG has been observed due to a higher macropore fraction (from 1 to 10 μm) by the presence of Al_2_O_3_ [156]. On the other hand, the preparation method has a direct impact on activity due to the type of species formed from both Cu [101] and metal oxides [173]. Comparing the traditional coprecipitation method in the presence of KOH and K_2_CO_3_ with coprecipitation via oxalate gel formation and mechanical mixing of oxides, the former allows higher yields to be achieved at 1,2-PG [174].

In catalysts based on Cu, Zn, Mg, and Al, on the other hand, the activity is a function of the basic properties of the support and the dispersion of the Cu metal particles [104,105]. It has been shown that the presence of ZnO generates a strong interaction with Cu, allowing the formation of small Cu metal particles with a high dispersion and good reducibility, which allows high yields to be reached for 1,2-PG [161]. In a liquid-phase continuous flow reactor, Zhou et al. employed a Cu-ZnO-MgO-Al_2_O_3_ catalyst with a Cu:Zn:Al molar ratio of 1:1:0.5, obtaining a glycerol conversion of 81% with 93% of selectivity to 1,2-PG at 200 °C, 4 MPa of H_2_ and 4.6 h^−1^ (LHSV) [175].

Other catalysts, based on Cu and Al, were also studied in the hydrogenolysis reaction. Indeed, it has been demonstrated that these catalysts showed a better performance than CuCr_2_O_4_ catalysts [154]. In these systems, the activity is a function of the Cu:Al molar ratio [147] and the preparation method. In all cases, the co-precipitation method was used, varying the precipitating agent. The results showed that the highest yields are obtained using NaOH as precipitating agent [176]. On the other hand, it has been reported that the presence of Cu^0^ and Cu^+^ species in the form of Cu_2_O improves the performance of the catalysts [176,177], in addition to others that improve the activity levels such as CuAl_2_O_4_ [177], CuAlO_2_, and CuAl_4_O_7_ [154]. In particular, the CuAl_2_O_4_ phase improves the reducibility and the ability of Cu to readily adsorb and desorb H_2_ [158].

Modification of Cu and Al catalysts with Ca led to an improvement in the acid-base properties of the catalyst by increasing the number of basic sites and allowing better dispersion of the Cu metal particles, leading to higher activity levels [143,155]. Mizugaki et al. prepared catalysts based on Cu nanoparticles in an amorphous aluminum oxide matrix (Cu-Al_2_Ox) from the reduction in Cu hydrotalcites. Their results showed very high yields for 1,2-PG: they obtained a glycerol conversion of 100% with a selectivity to 1,2-PG of 98% using very dilute solutions of glycerol in 1,4-dioxane [163].

Table 8 and Table 9 summarize the operating conditions and activity results of liquid-phase Cu-supported catalysts employing batch reactors and continuous flow reactors, respectively.

As with other metal phases, it has been reported that both the acidity of the support and the particle size are two important properties to take into account in catalyst design.

Most of the Cu-based supported catalysts have been prepared by the incipient wet impregnation technique, using Cu(NO_3_)_2_ as a precursor [127,151,152,181,189,191,193,194,195,196,197,198,199,200,201]. However, other methods such as co-precipitation [151,182,183,184], deposition–precipitation [185,202,203,204], and ion exchange [190,197] have been reported.

In the study of Cu catalysts, different supports have been reported, such as γ-Al_2_O_3_ [185,187,189,191,195,199,205], SiO_2_ [151,186,192,202,204], ZnO [182], TiO_2_ [178,199], MgO [179,185] and Cr_2_O_3_ [127], dealuminated ultra-stable Y-type zeolites (DUSY) [183] and HZSM-5 [188], mesoporous silica (HSM) [180], SBA-15 [198], and hydrotalcites [184,188].

For acidic supports, such as ZnO [182], Cr_2_O_3_ [127], γ-Al_2_O_3_ [185,187,189,191,195,199,205], and DUSY zeolite [183], it has been reported that the acid sites of the support favor the dehydration of the glycerol to AcOH which then hydrogenates over the Cu metal sites; however, the Cu active phase also generates the C-O bond cleavage.

On the other hand, when the acidity of the support is high, as in the case of zeolite HZSM-5 [188], the selectivity to 1,2-PG decreases due to the dehydration of glycerol to acrolein. Other supports such as TiO_2_ have acidic sites that favor the formation of side products such as 1-POH [178,199].

One of the most widely studied catalysts has been Cu/γ-Al_2_O_3_ [178,185,187,195,199]. In these systems, the selectivity to 1,2-PG has always been high (~90%) regardless of the Cu content of the catalyst [185]. It has been reported that the method of preparation of the Cu/γ-Al_2_O_3_ catalyst affects the catalytic performance. Vila et al. studied the preparation of these catalysts and their results indicated that the catalytic performance strongly depends on the calcination, reduction, and oxidation processes. In these processes, the presence of Cu^0^, Cu^+^, and Cu^+2^ species was evidenced. The results showed that the activity and selectivity to 1,2-PG increases in the presence of Cu^0^ and Cu^+^ species, while Cu^+2^ species are the least active in the hydrogenolysis reaction [195].

Regarding the γ-Al_2_O_3_ sites, a DFT study revealed that the hydrogenolysis of glycerol is facilitated by the presence of the acidic sites found on the alumina surface. The hydroxylation of these sites in the presence of water generates similar activity in terms of glycerol adsorption and AcOH formation as the Cu metal sites. However, the activation energy is lower in the case of the alumina surface sites, allowing high conversions to be achieved using this support [205].

The best results with Cu/γ-Al_2_O_3_ were obtained in continuous flow reactors with 1,2-PG yields of 70% at 250 °C, 4 MPa of H_2_, and 2 h^−1^ (WHSV) [187].

The modification of γ-Al_2_O_3_ with promoters has also been a variable studied in the literature. Heteropoly acids of the H_2_SiW_12_O_40_ type have allowed the reducibility of Cu particles to be improved on the support and to increase the acidity of the catalyst without promoting side reactions [189]. The modification with H_3_BO_3_ was also beneficial due to an effect on the dispersion of the Cu particles and the improvement of their hydrogenating capacity [191].

For neutral supports, such as SiO_2_, SBA-15, and other mesoporous silica (HSM), the published articles focused on the metal particle size. With respect to Cu/SiO_2_ catalysts, the preparation of the catalysts by the impregnation and co-precipitation methods led to better activity results with respect to the bulk Cu/SiO_2_ catalysts. Of the two methods, the co-precipitation method led to a greater dispersion of the Cu particles and better metal–support interaction [151,202,204]. Other more sophisticated preparation methods have shown even better performances for the Cu/SiO_2_ catalyst. For example, preparation by the hydrothermal ammonia evaporation method led to Cu^0^ species in the form of nanoparticles dispersed on the support surface. In these catalysts, the cooperative effect of Cu^0^ and Cu^+^ species are indicated to be responsible for the catalytic performance [192]. Another preparation, by coating SiO_2_ with Cu from a Cu-polyvinylpyrrolidone colloid, led to high yields for 1,2-PG [186].

The use of mesoporous silicas, such as SBA-15 and hexagonal mesoporous silica (HSM), allowed more active catalysts to be obtained than Cu/SiO_2_ [180], due to their textural properties of mesoporosity and specific surface area, which allowed high dispersions to be achieved in the active phase [190,198].

For basic supports, such as MgO [179,185] and hydrotalcites [184,188], the literature reports have indicated that higher basicity increases the levels of glycerol conversion and selectivity to 1,2-PG. It has also been reported that both MgO and hydrotalcites help to disperse Cu particles, which contributes to an improvement of the catalyst performance [179,184].

Since Cu is an active phase with good C-O bond cleavage ability, the incorporation of another metal in the catalyst design aims to increase the conversion of glycerol with respect to monometallic catalysts. In this regard, the study of Cu-Ru [206], Cu-Ag [196,207], Cu-Ni [208,209], and Cu-Zn [203,210] catalysts has been reported. Table 10 summarizes the operating conditions and activity results of bimetallic Cu catalysts in batch reactors.

In Cu-Ag/γ-Al_2_O_3_ catalysts, it has been reported that the addition of Ag generates a greater dispersion of Cu particles and facilitates their reduction, generating Cu^+^ species that are active in the hydrogenolysis of glycerol [196,203].

The study of Cu-Ni catalysts was reported on γ-Al_2_O_3_. Employing Cu-Ni/γ-Al_2_O_3_ with a Cu:Ni mass ratio = 3, Pudi et al. reported a 1,2-PG yield of 26% at 250 °C, 1 MPa of H_2_, and 5 h of reaction, assigning the result to the formation of a Cu_0_._75_Ni_0_._25_Al_2_O_4_-type mixed oxide [208]. Similar results with yields for 1,2-PG of 24% were obtained with a Cu-Ni/γ-Al_2_O_3_ catalyst in a continuous flow reactor at 250 °C, 4 MPa of N_2_, and 2 h^−1^ [187]. In both cases, the catalyst was prepared by wetness incipient impregnation. Yun et al. prepared the Cu-Ni/γ-Al_2_O_3_ catalyst by coprecipitation using mesoporous alumina, which leads to a better dispersion of the metal phase. For a Cu:Ni molar ratio = 9:1, the catalyst showed the best activity, and it became evident that the increase in the surface ratios of Ni^0^/(Ni^0^ + Cu^0^) and Cu^0^/(Cu^0^ + Cu^+2^) was responsible for the increase in yield to 1,2-PG [209]. The highest yield obtained with Cu-Ni/γ-Al_2_O_3_ systems was 57.8% at 220 °C, 4 MPa of H_2_, and 24 h [209].

The incorporation of Ru to Cu catalysts supported on carbon nanotubes (CNT) resulted in an increase in activity towards the formation of 1,2-PG, because it favored the dispersion of Cu particles and promoted their hydrogenation capacity by a reactive H spillover process from Ru to Cu [206]. The same spillover effect was found in Cu-Zn catalysts supported on MgO [210]. In these systems, the presence of Zn enhances the reducibility of CuO species and favors the dispersion of Cu^0^ particles, while providing suitable acid sites to improve the glycerol dehydration step [203].

Table 11 summarizes the main catalysts evaluated in vapor-phase continuous flow reactors.

From the set of Cu bulk catalysts studied, Cu/ZnO/ZrO_2_ and Cu/ZnO/TiO_2_ showed the lowest yields for 1,2-PG (12 and 15%, respectively). Although the catalysts showed high selectivity at AcOH (60–76%), due to the presence of TiO_2_ and ZrO_2_, the low selectivity to 1,2-PG could be due to the low H_2_ pressure (0.1 MPa) employed in the vapor phase condition [211].

Cu/Cr_2_O_3_ [213] and Cu/ZnO/Al_2_O_3_ [127] bulk catalysts, however, allowed yields to be obtained for 1,2-PG of 77% and 88%, respectively, even under less severe reaction conditions. In these catalysts, the acidic properties of the supports with the ability to dehydrate the glycerol to AcOH are combined with the presence of metallic Cu with the ability to hydrogenate the AcOH produced during the reaction.

The most studied Cu-supported catalysts have been the Cu/γ-Al_2_O_3_ ones [193,201,217,219]. In these systems, the activity levels depend on both the presence of Cu metal particles [217] and their exposed metal surface [201]. Sato et al. evaluated Cu/γ-Al_2_O_3_ catalysts in vapor-phase discriminating between dehydration and hydrogenation reactions. The authors indicated that the presence of Cu not only allows the hydrogenation of AcOH to 1,2-PG in H_2_ flow, but also intervenes in the dehydration of glycerol to AcOH when the reaction is carried out in N_2_ flow. At 250 °C, 0.1 MPa of N_2_ and 7.6 h^−1^ (WHSV), the authors reported a yield to 1,2-PG of 50% [193]. Optimizing the operating conditions and Cu content (15 wt.%), Dieuzeide et al. reported yields of 60% at 200 °C, 0.1 MPa of H_2_ and 30.6 h^−1^ (WHSV) [219]. Of all the studies reviewed to date, the maximum yield to 1,2-PG obtained with Cu/γ-Al_2_O_3_ was 96.8% at 230 °C, 1.4 MPa of H_2_ and 2.9 h^−1^ (WHSV) [217].

The modification of Cu/γ-Al_2_O_3_ catalysts with Ni and Ag allowed for an increase in the activity levels. In Cu-Ni/γ-Al_2_O_3_ catalysts, the presence of a Cu-Ni bimetallic site with a high exposed surface area and dispersion was responsible for the high yield obtained for 1,2-PG [214,220], while the presence of Ag in Cu-Ag/γ-Al_2_O_3_ catalysts allowed inhibiting undesired reactions that lead to the formation of side products, such as EG, improving the selectivity for 1,2-PG [218].

For other supported catalysts, dispersion and surface area effects, as well as acidic properties, have also led to high yields for 1,2-PG. Bienholz et al. used Cu/SiO_2_ catalysts and determined that the rate of the dehydration and hydrogenation reactions depends linearly on the exposed Cu metal surface [197]. In other catalytic systems, based on basic supports such as MgO [216] or neutral ones such as SBA-15 [200], it has been reported that Cu dispersion is fundamental to reach high levels of activity. In the case of acidic supports, such as zeolites, both the high dispersions of the Cu metal phase and the acidic properties of the support favor the formation of 1,2-PG [212].

Recently, Pandey et al. tested Cu-Zn/MgO catalysts in the vapor phase, finding that the presence of Cu^+2^ and Cu^+^ species facilitate the dehydration of glycerol to AcOH, while Cu metal particles hydrogenate AcOH to form 1,2-PG [214].

From the results obtained, it can be concluded that Cu-based catalysts exhibit high selectivity to 1,2-PG due to their ability to cleave C-O bonds. In the presence of neutral supports, high metal dispersion led to an increase in catalytic activity, with the species Cu^0^ and Cu^+^ being the most active. On acidic supports, Cu activity is enhanced, but other secondary products can be formed. The presence of other metals forming bimetallic phases enhance glycerol conversion at the expense of a decrease in 1,2-PG selectivity. The best results were obtained with Cu-Al_2_O_x_ (yield to 1,2-PG = 99.0%)

### 3.5. Ni Catalysts

In contrast to Cu catalysts, Ni catalysts (both bulk and supported) have not been so widely studied in the literature and have been used to obtain 1,2-PG in the liquid phase, employing batch reactors. The catalytic results of the Ni bulk catalysts are presented in Table 12.

Ni/Mg/Al catalysts, prepared by the coprecipitation method, showed low yields for 1,2-PG (2–3%), even when modified with Co as promoter [172]. Similar yields (5%) were obtained with a Ni_3_P bulk catalyst synthesized by a hydrothermal method at 150 °C in the presence of an ammonia solution of NiH_2_PO_2_ and NH_4_H_2_PO_2_ [221]. In the latter case, although the selectivity to 1,2-PG was 86%, the low yield was due to the low conversion achieved (5%), which could be associated with the reaction temperature (190 °C) and the high m_gly_/m_c_ ratio (100).

Higher yields for 1,2-PG (18%) were obtained with a commercial Ni-Raney catalyst at 230 °C, 4 MPa of H_2_, and 9 h reaction time [222]. At higher reaction times it was possible to obtain yields of 26% (24 h) [45] and 69% (44 h) [51]. For this catalyst, higher reaction temperatures promoted C-C bond cleavage reactions leading to the formation of side products [222]. The addition of Ag to Ni-Raney led to the formation of new Ni-Ag metal sites that suppressed C-C bond cleavage reactions [223]. A similar effect was found in ZnNiAl-type hydrotalcites, which were also found to be active and selective to 1,2-PG formation. In these systems, the formation of a Ni-Zn alloy inhibits C-C bond cleavage reactions and enhances the adsorption of the terminal hydroxyl group of glycerol by promoting C-O bond cleavage [224].

Table 13 summarizes the operating conditions and activity results of Ni-supported catalysts in batch reactors.

Supported catalysts based on Ni as the active phase showed, in general, high selectivity to 1,2-PG, comparable to those obtained with supported catalysts based on Ru and other noble metals. Although their ability to hydrogenate was lower than that of the noble metal-based catalysts, this was compensated for by the use of higher reaction temperatures and pressures, which led, on the other hand, to a higher deactivation by sintering of the active phase [232].

With respect to other metal phases based on non-noble metals, commercial Ni/C catalysts were demonstrated to be as active and equally selective to 1,2-PG as Cu catalysts [45,227]. The same trend was observed for commercial Ni/SiO_2_-Al_2_O_3_ catalysts compared to CuCr_2_O_4_ catalysts; however, the selectivity towards C-C bond cleavage was higher for the Ni catalyst [45].

Some authors have reported the study of commercial catalysts such as Ni/SiO_2_-Al_2_O_3_, where good particle dispersion was observed, so the hydrogenolysis reaction was carried out in a N_2_ atmosphere and the H_2_ was generated via the aqueous phase reforming of glycerol (APR) [233,234]. Maximum yields for 1,2-PG of ~29% were obtained at 240 °C, 3.3 MPa of N_2_, and 4 h [233]. The same result was obtained at 200 °C, 2.5 Mpa of H_2_, and 8 h of reaction [227].

The articles that have prepared and studied Ni-supported catalysts have mainly used Ni(NO_3_)_2_ [127,181,225,226,229,231,233,234,235,236,237,238] as the precursor, the impregnation method being the most widely used technique, although the co-precipitation [235] and deposition–precipitation methods have also been reported [226,237]. The most commonly used supports are based on activated carbon (AC) [229,239], SiO_2_ [191,225,236], γ-Al_2_O_3_ [127,181,225,230,240], CeO_2_ [226,237] and WO_3_ [231], and zeolites of the NaX, NaA, NaZSM-5, and NaMOR types [225].

Ni is a metal capable of participating in both C-O and C-C bond cleavage reactions due to its high electron density. In the presence of inert supports, the formation of products depends exclusively on the structural characteristics of the Ni metal phase. An example of catalytic systems where this phenomenon occurs is Ni/SiO_2_ [127,225,236]. Ni/SiO_2_ catalysts showed low activity (8–57%) with a low selectivity to 1,2-PG (44–60%) under moderate reaction conditions at 200 °C, 5–6 Mpa of H_2_, and 10 h [127,225]. The results obtained could be assigned to the ability of Ni to participate in C-C bond cleavage reactions in the presence of a neutral support [236].

In the presence of acid supports, different results are observed. For some catalysts based on acidic supports, such as Ni/γ-Al_2_O_3_ and Ni/CeO_2_, a certain tendency towards the formation of side products, such as EtOH, EG, 1-POH, and MeOH, was observed [127,225,226,230].

The modification of acidic supports with different basic promoters allows for a reduction in the formation of side products. When Ni/CeO_2_ was modified with MgO, a basic species, the selectivity to 1,2-PG was improved, probably due to a balancing effect of the acid-base properties by addition of MgO [237]. A similar effect was found for Ni/NaX catalysts, where the Na^+^ content allows the acidity of the catalyst to be moderated [225].

In other acid-supported Ni catalysts, however, the formation of side products was inhibited by the intrinsic characteristics of the acidic species. Ni catalysts supported on a modified sepiolite (MSEP) with WO_3_, for example, showed yields for 1,2-PG of 81% due to the high glycerol conversions achieved and a high level of selectivity for 1,2-PG. The results were attributed to the presence of WO_3_, with acidic characteristics, which allows the proper dispersion of Ni particles and inhibits C-C bond cleavage reactions [231]. In another work, the acidic properties of Ni catalysts supported on a silica–carbon composite (Ni/SiO_2_-C) were investigated, and it was determined that the presence of the carboxylic groups of the support promotes the dehydration of the glycerol producing AcOH, which then leads to the formation of 1,2-PG [228].

In order to improve the activity of Ni catalysts, the preparation of bimetallic catalysts has also been studied in the literature by several authors. The results of liquid phase activity employing batch reactors are shown in Table 14.

The use of bimetallic Ni catalysts with Ce [239,243], Ir [241], Cu [181,235,240], Zn [242], and P [244] has been reported.

Ni-Ce/SBA-15 catalysts showed better performances than Ni/SBA-15 catalysts due to a better dispersion of Ni on the support and a higher acidity provided by Ce. However, the maximum yields for 1,2-PG were 15% due to the low selectivity levels obtained [239]. In Ni-Ce/AC systems, the addition of Ce led to higher levels of selectivity to 1,2-PG, with yields of 59%. In this case, the same effect on Ni dispersion was reported due to the presence of Ce in the form of CeO_2_ [243].

In order to improve the selectivity of Ni catalysts, Ir was used as a promoter for the formulation of Ni-Ir/γ-Al_2_O_3_ catalysts. Although the presence of Ir^+δ^ species (0 < δ < 4) allowed selectivity values to be obtained to 1,2-PG of 83%; the yields achieved were low (20%) due to the low conversions obtained [241].

Ni-Cu/γ-Al_2_O_3_ catalysts prepared using the incipient wetting impregnation method [181] and the sol-gel method [235,240] showed maximum yields for 1,2-PG of 67% and 74%, respectively. In the first case, an adjustment of the Cu:Ni mass ratio (1:1) allowed the maximum level of activity to be obtained without losing selectivity to 1,2-PG [181]. In the second case, using a Cu:Ni ratio of 0.72:1, the maximum yield for 1,2-PG was obtained in the presence of formic acid as the H_2_ donor substance [235]. In both cases, the results were attributed to the formation of a highly dispersed Ni-Cu alloy [181] that promoted C-O bond cleavage and enhanced the activity with respect to Ni/γ-Al_2_O_3_ and Cu/γ-Al_2_O_3_ catalysts [235]. However, it was observed that the deactivation of the bimetallic catalyst was more pronounced due to higher coke generation and coke deposition on the active sites [240].

For other catalytic systems such as Ni-Zn/SiO_2_-C, the incorporation of Zn by means of the organometallic technique of surfaces on metals in the range 1.1–1.8 wt.% was responsible for the increase in activity and selectivity to 1,2-PG, due to the formation of an α-NiZn alloy that promotes the cleavage of the C-O bond in the glycerol molecule [242].

In Ni-P/γ-Al_2_O_3_ catalysts, on the other hand, the presence of P decreases the degree of Ni reducibility, generating NiO species that increase the selectivity to 1,2-PG, but decrease the glycerol conversion. The overall yield for 1,2-PG increases due to the presence of -POx groups generated by the presence of P [244].

With respect to obtaining 1,2-PG in the vapor phase, not many studies have employed Ni catalysts, due to the ability of this metal phase to form side products such as EG, EtOH, and gases. Ni/ZnO catalysts prepared by the quenching method on carbon spheres showed 1,2-PG yields of 45% at 235 °C, 3.1 MPa of Ar and 0.8 h^−1^ (WHSV). The results were attributed to the high dispersion of Ni particles on the support and space velocity optimization [245]. On the other hand, Ni-Ag/γ-Al_2_O_3_ systems showed similar yields for 1,2-PG at 200 °C, 0.1 Mpa, and 2.01 h^−1^ (WHSV), which were attributed to the higher dispersion of Ni due to the presence of Ag [238].

From the results above, it can be concluded that Ni-based catalysts are less active than Cu-based catalysts, showing lower selectivity values to 1,2-PG due to a higher ability to cleave C-C bonds. Better catalytic results were obtained in the liquid phase due to the generation of side products in the vapor phase. In order to increase catalytic activity, bimetallic formulations have been performed, although the best results were obtained modifying the acidity of the support, Ni/WO_3_-MSEP (yield to 1,2-PG = 85.4%).

### 3.6. Co Catalysts

Co catalysts have been less frequently employed than Cu and Ni catalysts, due to their lower intrinsic activity. These catalysts were employed for the production of 1,2-PG in the liquid phase, operating in batch reactors and continuous flow reactors. Table 15 and Table 16 present the main results in both types of reactors considering Co bulk catalysts.

Co bulk catalysts have been synthesized by co-precipitation [251,253], hydrothermal [248,250], microwave [249] and reduction in the presence of polyol [246,247] methods.

Cao et al. and Liu et al. employed the reduction method in the presence of polyol for the synthesis of Co nanocomposites [246] and Co-Cu nanostructures [247], respectively. Even though the Co nanocomposites were employed under more favorable reaction conditions, the Co-Cu catalyst led to a higher yield to 1,2-PG, due to the synergy of the Co-Cu bonding on the catalytic surface [247].

Other Co [248] and Co-Ni [250] nanostructures were synthesized by hydrothermal treatment. Again, the presence of a Co-Ni bimetallic bond led to a higher yield to 1,2-PG. Similar results were obtained when the Co-Ni catalyst was prepared by the microwave method [249].

Furthermore, Co-ZnO [251] and Co-Al [253] catalysts were prepared by the co-precipitation method, and both were highly selective to 1,2-PG. For Co-ZnO it was possible to establish a linear correlation between glycerol conversion and the exposed metal surface of Co [120], while for Co-Al, the authors studied the metal dispersion. The modification of this catalyst with a heteropolyacid of the H_4_[Si(W_3_O_10_)_4_] type allowed the level of conversion to be increased, due to a higher dispersion of the Co particles [253]. A similar Co-Al catalyst, but commercial, allowed a complete conversion of glycerol with a yield for 1,2-PG of 70%, the maximum obtained for Co bulk catalysts. The results were attributed to the ability of Al to dehydrate [252].

The Co-supported catalysts, on the other hand, were employed in the liquid phase using batch reactors (Table 17).

The most commonly used precursors for the preparation of Co catalysts have been Co(NO_3_)_2_ [127,255] and Co(CH_3_COO)_2_ [254], while impregnation [127], co-precipitation [255], and deposition–precipitation [254] have been used as preparation methods.

Reports have indicated that Co-supported catalysts are less active than Cu- and Ni-based catalysts under the same reaction conditions [127].

Co/SiO_2_ catalysts were selective to 1,2-PG, but not very active, leading to low yields (~5%) [127]. Co/MgO systems prepared by the deposition–precipitation method led to higher yields for 1,2-PG (~18%) due to the formation of an MgCo_2_O_4_-type spinel and a Mg-Co-O solid solution. The strong interaction between Co and Mg was responsible for the increased activity and stability of the Co catalyst, although the formation of Mg(OH)_2_ was evident after 9 h of reaction with a considerable increase in particle size [254].

Co/ZnAl catalysts prepared by the co-precipitation method were even more active (~40%) due to the formation of 16 nm Co nanoparticles produced during the activation process [255].

The previous results indicate that Co catalysts are less active than those that employ Cu or Ni. However, the selectivity to 1,2-PG is still high for these catalytic systems. The best results were obtained using the bulk catalyst Co-Al (yield to 1,2-PG = 70.1%).

## 4. Reaction Conditions and Effect of Operating Variables

Glycerol hydrogenolysis can be carried out in reactors operating in both liquid and vapor phases.

The liquid phase condition is established at moderate temperatures (120–260 °C) and high pressures (2–10 MPa) of H_2_, although in some cases N_2_ atmospheres or hydrogen donor substances are used for the hydrogenation stage [62,168,240]. This operating condition has the advantage of not vaporizing the reactive mixture, although it involves higher fixed costs associated with reactor design, due to the high operating pressures [45].

Batch and continuous flow reactors are those most commonly used to carry out the hydrogenolysis reaction in the liquid phase. While the former allow for the formulation of kinetic models and obtaining experimental data easily, the latter allow for the establishment of low contact times between the glycerol and the catalyst, which leads to greater catalytic stability [253,256,257,258,259]. Unlike continuous flow reactors, in batch reactors there is a loss in selectivity to 1,2-PG due to the generation of secondary products as the reaction proceeds [256].

The vapor phase condition, on the other hand, is established at high temperatures (200–300 °C) and low H_2_ pressures (0.1 MPa), so it is required to vaporize the fed glycerol solution to produce the reaction. As the concentration of crude glycerol in water is high (50–80 wt.%), high temperatures would be required to produce vaporization, which could lead to glycerol decomposition. For this reason, dilute solutions of glycerol in water are used when operating in the vapor phase. In addition, due to the low H_2_ pressures, dehydration and polymerization side reactions may be favored, leading to a loss in selectivity to 1,2-PG [260].

Fixed-bed reactors are the ones most commonly used to carry out hydrogenolysis in the vapor phase condition. In these reactors, the effect of temperature, pressure, space velocity, and gas flow are the main operating variables to take into account.

The effects of the main operating variables in batch and continuous flow reactors to carry out glycerol hydrogenolysis using different catalysts are presented below.

### 4.1. Effect of Initial Glycerol Concentration

The crude glycerol obtained as a by-product in biodiesel synthesis is found as an aqueous solution with a concentration in the range 50–80 wt.%. Research work has reported the effect of glycerol concentration in both batch reactors operating in the liquid phase (Table 18) and continuous flow reactors operating in the vapor phase (Table 19). In these tables, the glycerol concentration that maximizes the yield to 1,2-PG is informed.

The results indicate that the increase in glycerol concentration produces a decrease in conversion, regardless of the catalyst used and the operating conditions, whether liquid phase [52,148,167,185,243,251,261] or vapor phase [107,200,238]. These results would be explained by the increase in the number of glycerol molecules for a fixed set of active sites (since the catalyst mass remains invariant), leading to a lower availability of these sites [167,185,261]. The selectivity to 1,2-PG, on the other hand, decreases with the increasing glycerol concentration in the presence of Ru [52,262], Rh [52], and Ni [238] catalysts, whereas for Cu [167,185] and Co [251] catalysts it remains practically unchanged due to the intrinsic ability of these metals to promote C-O bond cleavage reactions.

### 4.2. Effect of Water Initial Concentration

Crude glycerol has a water concentration in the range of 20–60 wt.%. When the water content is very high, the hydrogenolysis of glycerol can lead to the formation of C-C bond cleavage products, such as EtOH [155]. However, a minimum water content in the initial reaction mixture is necessary, since its presence inhibits side reactions of glycerol dehydration to undesired [155], as well as condensation reactions between glycerol and side products, such as EG, EtOH, and MeOH [262].

Since water is generated during hydrogenolysis, it is better to work with a low water content in the starting solution in order to shift the equilibrium towards the formation of 1,2-PG. Dasari et al. reported an improvement in conversion levels when the initial water concentration was progressively reduced using a CuCr_2_O_4_ bulk catalyst [45]. The same effect was found for Ru/C catalysts that were tested in the presence of an Amberlyst 15 resin [262]. Xia et al. reported a drop in conversion values from 91% to 69% when the water content increased from 0% to 40% in an ethanol medium used as solvent in the presence of hydrotalcite type catalysts, Rh_0_._02_Cu_0_._4_/Mg_5_._6_Al_1_._98_O_8_._57_ at 180 °C, and 2 MPa of H_2_ [263]. Menchavez et al. also reported a drop in glycerol conversion from 80% to 43% with the maintenance of selectivity at 1,2-PG in the range 56–58%, when the water content increased from 0% to 40% in the presence of Ni/Ce-Mg catalysts [237].

Another aspect to take into account is that the presence of water affects the stability of the catalysts in the liquid phase. Hou et al. studied the effect of the initial water concentration in the reaction medium in the presence of Cu-ZnO catalysts and linked this effect to the textural and physicochemical properties of the catalyst. The presence of a higher water concentration generates an increase in the Cu and ZnO particle size, leading to a smaller metal area and lower acidity in the catalyst. In addition, the presence of water transforms the Cu^0^ particles to Cu^+2^, promoting the formation of EG and decreasing the selectivity to 1,2-PG. These processes result in a drop in the yield obtained at 1,2-PG [264]. The same effect on particle sintering and the subsequent loss of the metal phase by leaching was observed for Ni/Cu/TiO_2_ catalysts [265].

In continuous flow reactors in the vapor phase, it is convenient to minimize the water content to produce energy savings in vaporization [266]; however, at the same time water allows the vaporization of glycerol solutions at lower temperatures. Recently, a technical–economic study has been reported where the purification of 1,2-PG was carried out by distillation in stages. The first stage consists in separating the water from the liquid products, then separating the unreacted glycerol and finally the 1,2-PG from the rest of the products, especially EG. The economic study determined that the separation of water in the first distillation stage is the costliest step of the entire purification [267].

### 4.3. Effect of the Addition of Solvent

Since the water content in the starting glycerol solution affects the levels of conversion and selectivity to 1,2-PG, its replacement by other solvents has an impact on the development of the reaction. Both aprotic and aprotic solvents have been used among the solvents employed.

With respect to aprotic solvents, sulfolane and dioxane were used. In the presence of Rh/C catalysts, the use of sulfolane increased the glycerol conversion values, but decreased the selectivity to 1,2-PG, while the use of dioxane decreased the activity levels accompanied by a drop in the selectivity to 1,2-PG due to a promotion in the cleavage of C-C bonds; therefore, this indicates that neither are suitable for use in the hydrogenolysis reaction [58]. Co-Al catalysts showed similar results in the presence of sulfolane, with the activity towards 1,2-PG formation being the lowest achieved using this solvent [253].

The use of protic solvents has the advantage that many of them are hydrogen-donating substances that allow H_2_ to be generated in situ, thus improving the performance of the catalysts. MeOH, EtOH, 2-POH, formic acid [47,62,240,265,267], and EG [207] have been used as protic solvents for the production of 1,2-PG.

Gandarias et al. studied formic acid, 2-POH, and MeOH as H2 donor substances in the presence of Ni-Cu/γ-Al_2_O_3_ catalysts [240]. The maximum glycerol conversion and selectivity to 1,2-PG was obtained in the presence of formic acid with the improvement in hydrogenolysis activity corresponding to the following order: formic acid > 2-POH > MeOH.

On the other hand, Zhou et al. [168] reported that, in the presence of Cu/MgO catalysts prepared by the coprecipitation method, the improvement in activity and selectivity to 1,2-PG occurred following the order: 2-POH > EtOH > MeOH > formic acid.

Given these differences, the results suggest that the improvement in activity depends on the ability of the catalyst to produce H_2_ from the solvent used. In this regard, Yuan et al. employed a Cu/ZrO2 catalyst in the presence of formic acid and their results showed that the amphoteric character of the ZrO_2_ support is essential in producing the decomposition of formic acid and to generate in situ the H_2_ necessary for the formation of 1,2-PG [62].

Recently, a reaction mechanism study with in situ IR spectroscopy revealed that the decomposition of MeOH over a Cu-Zn-Al catalyst, for example, occurs via a first dehydrogenation step to form CO which is then converted to CO_2_ via the water–gas shift reaction [47].

Nevertheless, there are some drawbacks in the use of hydrogen-donating protic solvents. Since glycerol and hydrogen donor substances compete for the same active sites in the hydrogenolysis process, there should be a balance between the amount of glycerol and the amount of hydrogen donor substance to achieve high conversion and selectivity to 1,2-PG [240]. It has been reported that in the presence of 2-POH, a deactivation occurs in the liquid phase which is faster than that occurring when using water as the solvent due to the occupation of the active sites by the solvent and the deposition of carbonaceous products on the catalyst surface [240,265]. Furthermore, an economic feasibility study has recently reported that the use of formic acid compared to H_2_ is economically viable if its market price is less than USD 0.14 kg [267].

On the other hand, protic solvents have been reported as enhancing the solubility of H_2_. It has been reported that in the presence of alcohols, such as MeOH and EtOH, the solubility of H_2_ is much higher than in water [268]. For example, in the presence of Cu-Ag/γ-Al_2_O_3_ catalysts, the effect of the solvent in the reaction medium was studied using EtOH, MeOH, 1-POH, and EG. The results showed that EtOH led to the maximum conversion and selectivity to 1,2-PG, attributed to the higher solubility of H_2_ in EtOH [207]. In the presence of Cu-MgO catalysts, on the other hand, the use of 2-POH allowed a remarkable improvement in the catalytic performance due to its effect as the hydrogen donor molecule, while EtOH and MeOH allowed not only the better dissolution of H_2_ but also the reforming action of the catalyst on these alcohols and, therefore, the generation of H_2_ in situ [168].

As a disadvantage to the use of alcohols such as EtOH and MeOH, the formation of condensation products, such as glyceryl ethers, has been reported in some cases, leading to low selectivity to 1,2-PG [253].

The use of solvents with the aim of decreasing catalyst deactivation phenomena has also been reported. Cu/SiO_2_ catalysts prepared by the hydrothermal ammonia evaporation method were found to be highly stable when tested in a continuous flow reactor in liquid phase for 300 h of reaction at 200 °C and 5 MPa of H_2_, using MeOH as solvent [192]. Bienholz et al. reported an increase in glycerol conversion from 5% to 55% when water was replaced by 1,2-butanediol as reaction solvent at 200 °C and 5 MPa of H_2_ using 50 wt.% glycerol solutions in the presence of a CuO/ZnO bulk catalyst. The results showed that the use of 1,2-butanediol avoids the deactivation phenomenon because it does not favor an increase in the liquid-phase crystal size, as occurs with water [144]. It was also found that the high polarity of the solvent positively influences the formation of 1,2-PG, due to its easy removal on the catalytic surface with respect to aprotic solvents [269].

### 4.4. Effect of pH

The aqueous solutions used for the hydrogenolysis of glycerol in the liquid phase have a slightly acidic pH (pH ~ 5–6), either in very dilute glycerol solutions (10–40 wt.%) or in more concentrated ones (50–80 wt.%).

The initial pH of these solutions can vary with the addition of acids or bases, which has a particular impact on the catalytic activity, depending on the chemical species incorporated into the reaction medium. In addition, the mechanism by which the hydrogenolysis reaction occurs can be influenced by the pH.

Lahr et al. [42] studied the effect of the pH by modifying the reaction medium with CaO and CaCO_3_. For commercial Ru/C catalysts, they found that the more basic the medium, the higher the reaction rates of glycerol, 1,2-PG, and EG.

Furthermore, they proved that the selectivity to 1,2-PG does not vary with the pH of the medium, whereas the selectivity to EG does [42]. Following this idea, Feng et al. [44] tested the addition of different bases to the reaction medium in the presence of a Ru/TiO_2_ catalyst. Their results indicated that LiOH, NaOH, Na_2_CO_3_, and Li_2_CO_3_ increase the conversion of glycerol while keeping the selectivity to 1,2-PG unchanged. In all cases, a more pronounced increase in conversion was observed following the order Li^+^ > Na^+^ > K^+^, which was attributed to the size of the metal ions that are part of each of the tested bases.

This behavior could be due to the presence of OH- ions, which catalyze the dehydration of GLA to 2-HA, an intermediate species in the formation of 1,2-PG. The same results were obtained by Marinoiu et al. using CuCr_2_O_4_ [256] and by Yuan et al. using Cu hydrotalcites [184]. Maris et al. also proved that the addition of a base increases the Pt/C activity, promoting the maximum dehydration when 0.8 M solutions of NaOH and CaOH are added [43].

Dam et al. studied the presence of NaOH in the reaction medium, and their results showed that for an increase in pH from 5.6 to 12 the conversion increased from 25% to 45%, accompanied by an increase in selectivity to lactic acid (LA). This caused a drop in the selectivity values to 1,2-PG, in contrast to the results obtained by other authors [113]. Ahmed et al. also detected side products due to the reaction between EG and GLA under basic conditions, employing a Ru/AlF_3_-Al_2_O_3_ catalyst [75].

Chaminand et al. studied the addition of H_2_WO_4_ to aqueous solutions of 18wt.% glycerol, employing CuO/ZnO, Pd/C, and Rh/C catalysts, finding that glycerol conversion increases with slight changes in selectivity to 1,2-PG [58].

In addition, a summary of these effects is given by the work of Zhu et al. who evaluated a Cu-Ag/γ-Al_2_O_3_ catalyst for pH values of 4, 7, and 10, and concluded that the highest conversion and selectivity to 1,2-PG is obtained under neutral conditions, because acidity and basicity modify the textural properties of the catalyst. Acidic conditions promote the increase in specific surface area and dispersion of the active phase, leading to ester formation. Basic conditions lead to lower surface area values and to an increase in Cu particle size, decreasing the overall activity [203].

### 4.5. Effect of Catalyst Concentration

Several authors have studied the effect of catalyst concentration in the liquid phase using batch reactors, and in the vapor phase [215,220]. Most of the articles reported the effect in the presence of Cu [45,148,154,167,183,204,214,216] and Ni [155,231] catalysts, and have agreed that the increase in catalyst concentration increases the conversion levels, independently of the catalytic system under study.

With respect to selectivity, it has been reported that an increase in catalyst concentration results in a decrease in selectivity to 1,2-PG, due to the formation of side products, such as EG, MeOH, EtOH, and gases [45,148,155,183]. In some cases, very low catalyst concentrations have resulted in an accumulation of AcOH in the reaction medium due to the lack of active sites necessary for hydrogenation [155], whereas, in others, due to the selective nature of the catalyst, the selectivity to 1,2-PG remained constant against changes in catalyst concentration, even when this change was significant [216,231].

### 4.6. Effect of the Addition of Co-Catalysts

It is possible to add solids to the reaction medium that help to improve the catalytic activity of the catalysts employed. One of the most commonly used materials as co-catalysts has been ion exchange resins [53,262,270,271].

In the presence of Ru/C catalysts, the use of ion exchange resins of the Amberlyst 15 type allows for an improvement in the selectivity to 1,2-PG and an increase in the conversion due to the fact that the resin catalyzes the first stage of glycerol dehydration to AcOH [53,262,270]. In combination with these systems, the resin is highly selective to 1,2-PG without the presence of side products such as EG, 1-POH, and 2-POH.

The use of other ion exchange resins can significantly modify the selectivity to reaction products. It has been proven that the Amberlyst 70 exchange resin, while improving conversion (from 43% to 56%), produces a higher selectivity to 1-POH, EtOH, and EG. Other resins, such as Amberlyst DT, do not increase glycerol conversion and generate condensation products [262].

The disadvantage of exchange resins lies in their low thermal stability. The Amberlyst 15 resin, one of the most commonly used resins, starts to decompose at 120 °C. The Amberlyst 70 resin has a higher thermal stability, but decomposes above 180–190 °C [270,271].

Other alkyne sulfonic type resins have been employed given their higher thermal stability for use at temperatures above 180 °C. Prepared from the co-polymerization of 2-acrylamido-2-methyl-propanesulfonic acid with N,N-dimethylacrylamide and ethylene dimethylacrylate, these resins were found to be more selective towards 1,2-PG formation than the Amberlyst 70 resin. The results, employing a Ru/C catalyst in the presence of the resins, showed conversions of 23% with selectivity to 1,2-PG of 33% after 4 h of reaction [271].

Other substances, such as salts [51,272], ionic liquids [273], and acidic solids [258,261,262,269,274] have also been employed as co-catalysts in the hydrogenolysis reaction.

With respect to salts, it has been reported that the addition of phosphonium chloride to the reaction medium in the presence of Ni-Raney improves the selectivity to 1,2-PG, but subsequently hinders the separation of the catalyst from the reaction medium [51]. On the other hand, the use of Na_2_S in combination with Ru/C catalysts allows the suppression of C-C bond cleavage reactions leading to the formation of side products such as EG, while selectivity to 1,2-PG is favored given the increased ability of the catalytic system to dehydrate glycerol to AcOH [272].

The addition of ionic liquids, such as choline chloride with acid salts of ZnCl_2_ and FeCl_2_, has been shown to be efficient with Ru/C and Ru/TiO_2_ catalysts; however, the intrinsic effect of the ionic liquid with respect to the acid salts has not been discussed in depth [273].

With respect to other acid solids, there have been several reports. Balaraju et al. tested Ru/C catalysts with the addition of different acid solids. Of the set of acid solids studied, Nb_2_O_5_ and ZrO_2_ showed higher activity, with a linear correlation observed between glycerol conversion and acid site density [261]. SiO_2_-H_3_PO_4_ and SiO_2_-Al_2_O_3_ co-catalysts have been evaluated obtaining improvements in activity levels and selectivity at 1,2-PG following the acidity order SiO_2_-H_3_PO_4_ > SiO_2_-Al_2_O_3_ [262]. Other solids such as SiO_2_, MgO, Al_2_O_3_, H-ZSM5, TiO_2_, ZrO_2_, CeO_2_, and ZnO were employed as co-catalysts in the presence of Ni-Mo catalysts. Of these, ZnO proved to be the optimal co-catalyst, reaching the maximum yield to 1,2-PG. The results were attributed to the higher Lewis-type surface acidity present in the ZnO solid [258]. In another work, the authors proved that there is a linear correlation between the production rate of 1,2-PG and the specific surface area of ZnO crystals [275].

Zeolites of the H-ZSM5 type allowed increasing glycerol conversion in the presence of Ru/SiO_2_ and Ru/Al_2_O_3_ catalysts, but with a loss in selectivity to 1,2-PG due to the formation of side products, such as methane by C-C bond cleavage reactions [274].

### 4.7. Effect of Temperature

Temperature is a crucial variable in the operating condition of the reactors because, on the one hand, it allows the operating phase (liquid/steam) to be maintained and, on the other hand, it leads to obtaining the hydrogenolysis products of interest.

Dasari et al. were the first authors to present a section devoted to the study of the thermal effect [45]. Employing a CuCr_2_O_4_ catalyst in the range 150–260 °C and 1.4 MPa of H_2_, they observed that an increase in temperature causes an increase in glycerol conversion with a decrease in selectivity to 1,2-PG due to the formation of side products, such as EG, MeOH, and EtOH.

This thermal effect was subsequently studied by other authors using different catalytic systems with the objective of finding the temperature that maximizes the yield of 1,2-PG. Table 20 and Table 21 show the study of the thermal effect for different catalysts. For each of them, these tables show the thermal range studied, the operating conditions under which the study was carried out, and the optimum temperature that maximizes the yield at 1,2-PG.

The results of the thermal effect study allowed the global and apparent activation energies (E_a_) to be estimated for the hydrogenolysis reaction, using the equation based on the Arrhenius law. Table 22 shows the catalysts, the activation energies found, and the operating conditions under which the tests were carried out.

### 4.8. Effect of Pressure

In the same was as the temperature, the pressure allows the operating condition in the reactors to be maintained, and has an impact over the selectivity to the hydrogenolysis products.

In the liquid phase using batch reactors, it has been reported that an increase in H_2_ pressure generates an increase in glycerol conversion, as well as an increase in selectivity to 1,2-PG, using catalysts based on noble metals such as Ru [90,261] and non-noble metals such as Cu [45,154,160,185,204]. With some catalysts, however, no change in selectivity to 1,2-PG was observed, suggesting that the nature of the catalyst is highly selective to this product [101,121,185]. This effect was also evaluated in the liquid phase using continuous flow reactors of the trickle bed-type. The results obtained were similar to those reported in batch reactors [256]. There are very few studies where the effect of pressure has been studied in continuous flow reactors in the vapor phase, but the results indicated the same effect on the conversion and selectivity to 1,2-PG [214,216,220,266]. Generally speaking, the results obtained have been attributed to a higher solubility of H_2_ in the liquid phase, which allows the hydrogenation of AcOH to take place and shifts the equilibrium towards the formation of 1,2-PG [90,102,160,185,254,256,261].

Operating at higher pressures generates two benefits. On the one hand, it allows the liquid phase condition to be maintained, and on the other hand, it increases the solubility of H_2_ improving the selectivity to 1,2-PG. However, reactors operating at higher pressures generate higher fixed costs associated with the equipment, and also a higher operating cost [45].

It has also been reported that an excessive increase in pressure can lead to a drop in conversion and even a loss in selectivity to glycols. On the one hand, the drop in conversion values has been attributed in some catalytic systems to the competition between the adsorption of H atoms on the catalyst with the adsorption of reaction intermediates [90]. At high pressures, H_2_ adsorbs on the active sites blocking part of them for glycerol adsorption [117]. In cases where the dehydrogenation–dehydration–hydrogenation mechanism prevails, the drop in conversion can be attributed to the fact that the dehydrogenation step is thermodynamically disadvantaged in the presence of high H_2_ pressures [148].

On the other hand, several reports have agreed that an excessive H_2_ pressure promotes the formation of 1-POH or 2-POH, or even the cleavage of C-C bonds. With respect to noble metal-based catalysts, such as Ru [261] the selectivity to 1,2-PG decreases at the expense of EG formation. Cu-based systems have shown similar results with a drop in selectivity at 1,2-PG due to the formation of side products [95,216]. Other Ni-based catalysts have shown the formation of EG [225,231,284] and propanols [237] due to the same reason.

The use of low H_2_ pressures under conditions of high glycerol concentrations, however, may lead to the generation of condensation products, due to the reaction between glycerol and AcOH, which usually occurs because of the lower hydrogenating activity due to the lack of H_2_ [160,285].

Thus, the pressure must be properly selected to maintain high conversion levels without losing selectivity to 1,2-PG. Table 23 and Table 24 show the catalytic systems used to study the effect of pressure, their reaction conditions, and the optimum pressure which maximizes the yield to 1,2-PG.

### 4.9. Effect of H_2_ Flow

In cases where hydrogenolysis is carried out in flow reactors, both in the liquid phase (trickle-bed reactors) and the vapor phase (fixed bed reactors), it is important to study the effect of H_2_ flow. Table 25 shows the results of the H_2_ flow study using different catalysts.

The results indicate that an increase in H_2_ flow causes an increase in glycerol conversion due to the hydrogen availability on the catalytic surface. It has been observed that, in some cases, the selectivity to 1,2-PG increases with the increasing H_2_ flow up to a certain point, after which it decreases due to the generation of side products [107]. In other cases, the selectivity to 1,2-PG remains constant, regardless of the H_2_ flow employed [200].

The results obtained in this section indicate that operating variables in batch and flow continuous reactors must be optimized to obtain high yields of 1,2-PG, and the optimization depends on the catalytic system.

## 5. Stability and Deactivation of Catalysts

From previous sections, it is concluded that catalysts based on noble metals (Ru, Pt, Pd) and those based on non-noble metals (Cu, Ni, Co) are active and selective towards the formation of 1,2-PG.

A relevant aspect in the study of these catalysts is the possible ways of deactivation in successive reaction cycles or in continuous operations. In particular, the deactivation of the catalysts during the hydrogenolysis reaction of glycerol, both in liquid and vapor phase, can be due to several factors, among which the following stand out: structural changes of the supports, oxidation and loss of the active phase by leaching, adsorption of carbonaceous species and formation of carbon deposits, sintering of the active phase and deactivation due to the presence of impurities when using crude glycerol.

This section discusses the stability and the main deactivation mechanisms that affect the performance of the catalysts, as well as their reuse in successive reaction cycles or in a continuous operation.

### 5.1. Structural Changes of the Supports

When glycerol hydrogenolysis is carried out in the liquid phase, the structure of the catalytic systems can be irreversibly modified due to the hydrothermal condition of the reaction [136]. The liquid-phase operating condition is detrimental to most of the traditional catalytic systems. The stability of the materials is affected due to the combination of three factors: temperature, pressure, and the presence of hot water [180]. It has been reported, for example, that the high surface tension of water generates the agglomeration of Cu nanoparticles on the ZnO surface generating the decrease in the amount of active sites [269]. In other cases, water has been replaced by solvents to avoid deactivation (see Section 4.3.).

In the case of supported metal catalysts, the support structure may undergo pore collapse, resulting in a drop in the specific surface area and total pore volume, and eventually even a loss of the active phase by leaching. Modifications in the structure of bulk catalysts with similar effects on textural properties have also been reported. Meher et al. employed a Cu/Zn/Mg/Al bulk catalyst in two consecutive reaction cycles and observed a significant drop in the conversion value. This deactivation was assigned to the pore blockage and loss of specific surface area [172].

Among the traditional supports, γ-Al_2_O_3_ undergoes hydration in an aqueous medium and transforms to boehmite between 200 and 250 °C, which generates changes in surface acidic properties [286,287]. When δ-Al_2_O_3_ and θ-Al_2_O_3_ are employed as supports, similar results have been obtained, and the sintering of the metal phase due to changes in the structure of the supports has also been observed [288,289].

The MgO support, on the other hand, has been widely used in the preparation of Cu catalysts, but it was found to be unstable in liquid phase due to its transformation to Mg(OH)_2_ by interaction with H_2_O molecules and subsequent leaching [149].

The presence of hot water can also cause the dissociation of Si-O-Si bonds on the SiO_2_ support [290]. Other ordered silicas of the mesoporous type have also shown changes in their structure in the liquid phase condition, e.g., SBA-15 evidenced a drop of 97% in specific surface area after hydrothermal treatment in hot water at 200 °C for 12 h due to pore collapse [291,292].

More complex SiO_2_-Al_2_O_3_ supports (SIRALOX 30, Sasol) have also shown a loss in specific surface area due to pore collapse in hot water at 225 °C and 2.5 MPa [293].

On the other hand, carbon-based supports such as activated carbons, graphitic carbon of different nature, and traditional carbon-modified supports (TiO_2_-C) have shown better liquid-phase behavior due to the hydrophobic characteristics of carbon that protect the active sites from hydrolytic attack by water and maintain the catalytic activity [294].

Other supports, such as Fe_3_O_4_, are not structurally modified and can be employed as support materials [117].

In the vapor phase, on the other hand, traditional supports undergo less modification in their structure, due to the absence of liquid water at high pressure and temperature. It has been reported, for example, that a Ni-Ag/γ-Al_2_O_3_ catalyst was stable at 200 °C and 0.1 MPa of H_2_, with conversion levels of 72–79% and selectivity at 1,2-PG of 58% after 420 h of reaction [238].

### 5.2. Oxidation and Loss of Active Phase by Leaching

With respect to the active phase, the oxidation of the metals and their leaching are two frequent deactivation phenomena, especially in the liquid phase condition.

The leaching of the active phase has been reported for several catalytic systems, with Cu catalysts being the most widely studied one. Huang et al. prepared a CuO/SiO_2_ catalyst that was stable in the liquid phase condition at 200 °C and 6.0 MPa of H_2_ for 200 h, deactivating afterwards mainly by the leaching and sintering of the active phase [151,169]. Cu leaching was also observed on Cu-Fe bulk catalysts. After three reaction cycles of 10 h each, at 190 °C and 4.1 MPa of H_2_, the conversion decreased from 47% to 40% with a maintenance of selectivity at 1,2-PG of 92% [145]. Cu/Al_2_O_3_ catalysts were employed in four 24 h reaction cycles at 200 °C and 4.0 MPa of H_2_. A drop in conversion from 75% to 70% was reported between the first and fourth reaction cycles, with a loss of selectivity at 1,2-PG from 95% to 88%, assigned to the loss of surface acidity due to a lower content of the CuAl_2_O_4_ and CuAl_4_O_7_ phases, as well as to the loss of the CuAlO_2_ phase [154]. Durán-Martin et al. studied the deactivation phenomenon in Cu-ZnO catalysts. After five reaction cycles of 8 h at 200 °C and 2.4 MPa of H_2_, the conversion decreased from 16% to 5% with an increase in selectivity at 1,2-PG from 44% to 73%. The yield at 1,2-PG decreased drastically due to the sharp drop in conversion. Characterization results revealed a significant leaching of Zn due to the acid attack of the reaction medium, formation of Zn complexes due to the presence of reaction intermediates, and oxidation of Cu [295]. Cu-Cr catalysts, prepared in the presence of propylene oxide as the reducing agent, showed slight deactivation after three reaction cycles of 4 h at 130 °C and 2.0 MPa of H_2_. Characterization results of the catalyst used showed an increase in crystallinity and an increase in particle size, as well as Cu leaching [80]. After three reaction cycles of 8 h at 200 °C and 3.5 MPa of H_2_, Cu-Ag/γ-Al_2_O_3_ catalysts suffered a drop in conversion from 66% to 27% along with a decrease in selectivity to 1,2-PG. The results indicated the loss of the active phase of Cu and Ag, in addition to a decrease in surface acidity [207].

Deactivation by leaching was also observed for noble metal-based catalysts, such as Pt/CeO_2_ and Pt/La_2_O_3_ [111]. In particular, Pd/CoO catalysts showed a sharp drop in conversion from 70% to 45% after five reaction cycles of 24 h at 180 °C and 4.0 MPa of H_2_ using 12 wt.% glycerol solutions in 2-POH. It was attributed to possible Pd leaching, although it was not demonstrated [131]. Recently, the deactivation of Pd-Zn/ZrO_2_ catalysts was reported, with a drop in reaction rate of 70% after seven reaction cycles of 4 h each at 220 °C and 6.0 MPa of H_2_, due to the oxidation and leaching of Zn. To maintain the activity, the authors added ZnO in excess to the reaction medium so that the Zn^+2^ ions are reduced in situ and generate the Pd-Zn alloy again on the catalytic surface [138].

### 5.3. Adsorption of Carbonaceous Species and Formation of Carbon Deposits

The formation of carbon deposits has been reported mainly in the vapor phase, due to the higher temperatures used. The most affected catalysts have been those based on noble metals (Pt, Ru, Pd) due to their ability to cleave C-C bonds and, to a lesser, extent on catalysts based on non-noble metals (Ni, Cu).

With respect to noble metals, Ru/TiO_2_ [105], Pt/CeO_2_ [111], Pt/La_2_O_3_ [111], and Pt-Re/CNT’s [123] catalysts have been reported to have suffered deactivation by formation of carbonaceous deposits. The presence of a bimetallic phase, however, has improved the stability of some of these catalytic systems. In Ru-Cu/TiO_2_ catalysts, for example, the addition of Cu to the Ru phase proved to be effective in inhibiting the deactivation that occurs in monometallic Ru/TiO_2_ catalysts, because the C-C bond cleavage reactions that generate the strong adsorption of intermediates on the catalytic surface are inhibited [105].

Ni/γ-Al_2_O_3_ catalysts showed vapor phase deactivation due to coke formation on Ni species and formation of nickel carbide (Ni_3_C) in addition to oxidation of the Ni metal phase, during reaction tests at 200 °C and 0.1 MPa of H_2_/Ar employing 3% *v*/*v* aqueous glycerol solutions after 6 h of reaction [296,297].

Durán-Martín et al. prepared Cu-ZrO_2_ catalysts and observed a reversible deactivation generated by the deposition of carbonaceous compounds, and by a slight oxidation and sintering of the Cu metal particles [141]. Cu catalysts supported on hexagonal mesoporous silica (HMS) have been reported as unstable. The formation of carbonaceous species was responsible for a 50% drop in the second cycle conversion [180]. On acidic supports, such as Y-type zeolites, the effect can be linked to the excessive acidity of the support, which generates C-C bond cleavage and the formation of carbon on the surface of the catalysts [212]. Sepulveda et al. did not detect the presence of carbonaceous species in CuCr_2_O_4_ catalysts in the range of 190–245 °C and 0.1–1.8 MPa; however, they did detect the presence of polyglycerol oligomers. The authors explained that the presence of these species was the cause of catalyst deactivation [298].

In most cases, deactivation by carbon formation and the adsorption of carbonaceous species results in a reversible phenomenon, and the catalysts can be fully or partially regenerated by an oxidation and subsequent reduction process. In the case of Ni/γ-Al_2_O_3_ catalysts, an oxidation–reduction process at 500 °C successfully regenerated the catalyst [296,297], while in the case of the Cu-ZrO_2_ catalyst, a treatment in H_2_/Ar at 300 °C for 1 h succeeded in regenerating the catalyst and partially reaching the activity level of the fresh catalyst [141].

### 5.4. Sintering

Sintering is the main deactivation mechanism reported for this reaction. Most of the work has indicated the effect of sintering on catalysts based on non-noble metals, mainly Cu.

It has been reported that Cu/SiO_2_ catalysts suffer deactivation in the liquid phase condition due to the loss of metal surface area [198]. Huang et al. prepared Cu/SiO_2_ catalysts employing colloidal SiO_2_ and commercial SiO_2_ and evaluated them in three successive reaction cycles of 12 h each. Although both catalysts were active and selective to 1,2-PG, the commercial Cu/SiO_2_ catalyst was more stable. The deactivation of the colloidal Cu/SiO_2_ catalyst was attributed to the agglomeration and sintering of the Cu particles [202]. Zhu et al. prepared the same Cu/SiO_2_ catalyst and found similar results with rapid deactivation due to the agglomeration and sintering of Cu particles from 17 nm to 34 nm [299]. Cu/SBA-15 also showed deactivation due to an increase in Cu particle size, with a drop in conversion from 90% to 80% with a decrease in selectivity to 1,2-PG from 84% to 60% after 10 h of reaction at 220 °C and 0.1 MPa of H_2_ [200]. The Cu/MgO catalytic system also showed an increase in Cu particle size with reaction time [181].

For Cu and ZnO, the most common deactivation phenomenon was sintering [157,182,300]. Bienholz et al. determined that the Cu/ZnO catalyst deactivates after the first use in reaction at 200 °C and 5 MPa of H_2_ after 7 h of reaction, with a drop in conversion from 55% to 38% occurring in the second use due to the sintering of the Cu particles [157]. Similar results were reported by Du et al. [300]. On Cu catalysts supported on ZnO modified with a USY-type zeolite, a drop in conversion from 95% to 87% was also observed with a maintenance in selectivity to 1,2-PG of 58% after three reaction cycles of 5 h each at 220 °C, and 3.5 MPa of H_2_ due to the aggregation of the Cu particles and subsequent size growth, thus decreasing metal dispersion [182].

More complex Cu-Zn-Mg-Al systems also showed a drop in conversion from 98% to 30% after thre reaction cycles of 12 h each at 210 °C and 4.5 MPa of H_2_ with a maintenance in selectivity to 1,2-PG, primarily due to the agglomeration and sintering of Cu particles [161].

CoCu nanocomposites showed a drop in conversion from 40% to less than 20% with a maintenance in selectivity to 1,2-PG of 60–65% after three reaction cycles of 7 h at 220 °C and 3 MPa of H_2_. Agglomeration and sintering of the particles were the main cause of deactivation [247].

Recently, Mitta et al. reported a drop in glycerol conversion values and selectivity to 1,2-PG of 50% and 20%, respectively, due to an increase in CuO particle size in Cu catalysts supported on Y zeolites [212].

With respect to noble metal-based catalysts, not many studies have been reported where the main deactivation mechanism is sintering, probably because the metal contents are low and there is a higher metal-–support interaction that inhibits or decreases this deactivation phenomenon.

Zhou et al. reported the phenomenon of sintering deactivation on Ag/Al_2_O_3_ catalysts after being used in a reaction cycle at 220 °C and 1.5 MPa of H_2_ for 10 h. The activity, which initially showed a 46% conversion, dropped to 25% with a maintenance of 93% selectivity to 1,2-PG, and the particle size of the active phase increased from 10 nm to 30 nm. The calcination of the catalyst in air at 400 °C for 3 h restored the activity levels, causing a re-dispersion of the active phase [301]. On the other hand, Pt/TiO_2_ and Pt/Al_2_O_3_-SiO_2_ catalysts showed an increase in Pt particle sizes due to a sintering process at 210 °C and 6 MPa of H_2_. This effect was lower for the TiO_2_ support, probably due to a higher metal–support interaction [110].

In the vapor phase, Ru/γ-Al_2_O_3_ catalysts suffered severe deactivation after 7 h of vapor phase reaction at 230 °C and 0.1 MPa of H2 due to the aggregation of Ru particles [108]. Yan et al. tested Pt-Ni/γ-Al_2_O_3_ catalysts in five reaction cycles of 3 h each at 240 °C and 1 MPa of N2, showing a slight drop in conversion and selectivity to 1,2-PG [121]. Other Pd-Ni bimetallic systems showed slight changes in conversion with a maintenance in selectivity at 1,2-PG after four reaction cycles of 12 h each at 220 °C and 6 MPa of H_2_ [140].

The addition of substances inhibiting the aggregation and subsequent particle growth was also implemented as a strategy to avoid the sintering phenomenon. Catalytic systems based on Pd-Zn supported on ZnO-Al_2_O_3_ showed high stability over time in the liquid phase condition. After five reaction cycles of 3 h at 230°C and 3 MPa of H_2_, the glycerol conversion (53%) and the selectivity to 1,2-PG (92%) remained unchanged. These results were attributed to the role of Al_2_O_3_ which suppresses the aggregation of Pd-Zn bimetallic particles and protects the ZnO surface from erosion by water [302].

Cu-Ru/CNT catalysts were modified by mesoporous SiO_2_ coating in order to improve their stability. The catalysts maintained their activity for four reaction cycles at 210 °C and 7.5 MPa of H_2_, with a glycerol conversion of 45% and a selectivity to 1,2-PG of 90%. The results were attributed to the ability of the mesoporous SiO_2_ to avoid a sintering of Cu and Ru particles (~1.8 nm) [303]. On the other hand, the addition of Ga_2_O_3_ in the preparation of Cu-ZnO catalysts was reported to prevent the sintering of Cu particles within the ZnO phase [157]. With respect to the Cu/SiO_2_ catalyst, the addition of H_3_BO_3_ decreased the sintering effect by improving its stability up to 72 h of reaction [299]. The effect of H_2_BO_3_ addition was also studied on Cu/Al_2_O_3_ catalysts which were found to be active and selective at 1,2-PG for 60 h of reaction at 250 °C and 6 MPa of H_2_ in a liquid-phase continuous flow reactor [191]. Furthermore, the incorporation of rare earth elements, such as Y and La, improved the stability of a Cu/SiO_2_ catalyst by inhibiting the leaching and sintering of the active phase, making it stable for 600 h of reaction at 200 °C and 6 MPa H_2_ [304]. The addition of La to Ru/ZrO_2_ catalysts improved the stability of the catalyst. For a Ru content of 2 wt.% and a La content of 1 wt.%, the catalyst could be employed in seven cycles of 8 h at 190 °C and 6 MPa of H_2_, showing no apparent deactivation or changes in selectivity [76].

### 5.5. Deactivation Due to the Presence of Impurities Using Crude Glycerol

Crude glycerol contains many impurities, including soaps, sodium, or potassium salts (mainly chlorides and sulfates), organic matter such as glycerides, sulfur compounds from transesterified oils and fats, and traces of alcohol, which do not react in the transesterification process [90,305,306,307]. It usually consists of a solution of glycerol and water with a concentration of glycerol in the range 50–88 wt.%. For this reason, its use as an alternative to the use of technical or refined grade glycerol has some effects on the stability of the catalytic systems.

With respect to Cu/Al_2_O_3_ catalysts modified with B_2_O_3_, they were evaluated using 10 wt.% aqueous solutions of different glycerol qualities: pharmaceutical grade (refined), technical and crude, obtaining the following order of activity and selectivity at 1,2-PG: pharmaceutical glycerol > technical glycerol > crude glycerol. The results suggested that although the presence of water could be a thermodynamic barrier to decrease the yield of 1,2-PG, impurities can adsorb on the catalytic surface and compete for the same active sites as glycerol [191]. Similarly, Cu-Al catalysts showed 50% conversions with a selectivity to 1,2-PG of 75% when employed in a liquid-phase continuous flow reactor using crude glycerol at 220 °C and 2 MPa of N_2_. In the presence of refined glycerol solutions, on the other hand, 90% conversions were obtained with selectivity values to 1,2-PG of 22–25% [177].

It has been reported that sodium or potassium salts, mainly chlorides and sulfates, are generated as a consequence of neutralization with HCl or H_2_SO_4_ after transesterification processes of fats and oils in biodiesel production [90,306,307]. The presence of these salts could produce pore blocking in the catalysts or even deactivate the active sites by incorporation into the active phase. Recently, Rajkhowa et al. studied the deactivation phenomena of a commercial Cu catalyst tested in a trickle-bed reactor. The presence of Cl^−^ in the reaction medium simulating the presence of sodium chloride from crude glycerol resulted in catalyst deactivation due to sintering by incorporation of Cl into the Cu particles [307]. Other salts such as Na_2_SO_4_, however, did not produce significant changes. In this respect, Balaraju et al. employed Ru/TiO_2_ catalysts and evaluated them in the liquid phase condition in the presence of crude glycerol and refined glycerol containing Na_2_SO_4_ as impurity. The results showed slight changes in conversion (46–42%) and selectivity to 1,2-PG (63–59%) indicating that the catalysts are resistant to the presence of this impurity in crude glycerol [90]. Cu-MgO catalysts were active and selective to 1,2-PG even with crude glycerol and a synthetic solution of glycerol and Na_2_SO_4_. In both cases, the catalyst showed a drop in conversion with respect to that obtained by using glycerol of analytical grade, maintaining a selectivity to 1,2-PG of 92% in all cases [167].

The presence of sulfur compounds from fatty acid methyl esters, which are part of oils and fats, are poisons for catalysts, mainly for Cu and Ru [272]. The results obtained by Rajkhowa et al. employing a commercial Cu catalyst showed that the conversion decreases by sulfur content and that the deactivation is more pronounced when the temperature is higher, due to the higher adsorption of sulfur on the active sites [307].

With respect to glycerides (mono-, di- or triglycerides), they can be found in crude glycerol because they come from biodiesel synthesis. Due to their nature, glycerides can foul the catalytic surface or act as coke precursors. It has been reported, for example, that the presence of glycerides caused deactivation of a commercial Cu catalyst due to the blocking of the catalyst active sites [307].

From the above, it can be concluded that supports and metal active phases suffer from deactivation in glycerol hydrogenolysis, both in the liquid and in the vapor phase. Traditional supports (Al_2_O_3_, MgO, SiO_2_) modify their structure and/or their textural properties, requiring other materials such as carbonaceous or metal oxides to be used as catalytic supports. As regards metal phases, the ones based on noble metals (Pt, Ru, Pd) are more likely to suffer deactivation associated with the adsorption of carbonaceous species and the formation of carbon, especially in the vapor phase, due to their ability to cleave C-C bonds. The non-noble metal phases (Cu, Ni, Co) are usually more susceptible to sintering and to oxidation and the loss of the active phase by leaching. Finally, the impurities present in crude glycerol affect the catalytic performance, modifying activity and selectivity to 1,2-PG depending on the catalytic system and the nature of impurity.

## 6. Kinetic Models

Most studies focus on kinetic studies in liquid phase conditions and batch reactors. Moreover, a few of them show the stability results of the catalyst in the selected reaction conditions [162,175,251,268] and ensure kinetic control in the absence of external and internal gradients to mass transfer [42,148,162,175,251,268,278,279,284].

Kinetic models based on the power law [98,137,152,251,268,278,281] and Langmuir–Hinshelwood–Hougen–Watson (LHHW) type have been developed, considering one [42,272,281,282] or two types of active sites [162,175,279,284,285] and in some cases the phenomenon of competition for active sites [42,175,272,279,282,284,285]. The values of activation energies have been calculated for the overall reaction rate [268,281,282], activation energies for the dehydration and hydrogenation steps [175], and in some cases, in the presence of poisons such as sulfur [272].

### 6.1. Kinetic Models Based on the Power Law

Kinetic models based on the power law use Equation (1) to express the rate of glycerol consumption (−r_gly_) in terms of a kinetic coefficient (k) and the concentrations of glycerol (C_gly_) and H_2_ in the reaction medium (C_H2_), with α and β being the partial orders of reaction with respect to glycerol and H_2_, respectively. Sometimes, the H_2_ concentration in the liquid phase is usually replaced by the H_2_ pressure in the vapor phase in contact with the glycerol solution (P_H2_).

(1) $$(-{\;r}_{\mathrm{gly}})\;=-\frac{{\mathrm{dC}}_{\mathrm{gly}}}{\mathrm{dt}}{=k\;C}_{\mathrm{gly}}^{\alpha }{\;C}_{H2}^{\beta }$$

Table 26 shows the different catalysts used to obtain the kinetic models based on the power law, the reaction conditions used, and the partial orders of reaction obtained in each case.

In some of the models, the experiments indicated that the rate of glycerol consumption strongly depends on its concentration rather than on the liquid phase H_2_ concentration, with α > β [137,251], while in others the reverse result was obtained [148,268].

### 6.2. Langmuir–Hinshelwood–Hougen–Watson (LHHW) Kinetic Models

Table 27 summarizes the kinetic expressions found for glycerol consumption using LHHW-type models.

The first study was reported by Lahr et al. who used a commercial Ru/C catalyst (5 wt.%) in the presence of bases (CaO and CaCO_3_) and performed kinetic tests at 7 Mpa of H_2_, considering pH values of 8 and 11.7, a glycerol concentration of 10 wt.%, 1,2-PG and EG concentrations of 10 wt.%, and reaction times up to 75 min. Due to the high pressures used, H_2_ was not considered as an adsorbed species on the catalytic surface because of its high availability in the reaction medium. The kinetic data were fitted with a LHHW model considering the competition for active sites by glycerol, 1,2-PG, and EG. The results showed that glycerol adsorbs more strongly than 1,2-PG and EG, which compete with each other, with EG being preferred for active sites. The modeling allowed a kinetic expression to be obtained for glycerol consumption according to Equation (2) [42]:

(2) $$(-r_{\mathrm{gly}})\;=\frac{k_{\mathrm{gly}}C_{\mathrm{gly}}{}^{1.5}}{{1+K}_{\mathrm{gly}}{\;C}_{\mathrm{gly}}{+K}_{\mathrm{EG}}{\;C}_{\mathrm{EG}}{+K}_{1,2\mathrm{-PG}}{\;C}_{1,2\mathrm{-PG}}}$$

In another work, the same authors studied the influence of sulfur as a catalyst poison by adding Na_2_S in the reaction medium. The kinetic expression of Equation (12) was maintained but incorporating the thermal effect. The results showed that the presence of sulfur has a strong effect on the activation energy of the overall glycerol hydrogenolysis reaction, whose range of variation was 44–96 kJ mol^−1^, tending to increase with sulfur content [272].

Using a Co-Pd-Re/C catalyst in the presence of NaOH, Xi et al. developed a kinetic model from the results obtained in a trickle-bed reactor. The tests were carried out in the range 180–202 °C, pressures between 3.3 and 13.3 Mpa of H_2_ using 40 wt.% glycerol solutions in the presence of NaOH with concentrations between 0.1 and 0.6 M. The kinetic expressions of the model were developed considering three steps of the reaction mechanism: (1) the dehydrogenation of glycerol to glyceraldehyde over the active sites of the catalyst, (2) the dehydrogenation of glyceraldehyde to pyruvaldehyde over the same catalytic site, (3) the hydrogenation of pyruvaldehyde to 1,2-PG. The kinetic expression (Equation (3)) is as a function of glycerol concentration, H_2_ concentration in the liquid phase, and OH^−^ present in the reaction medium (due to the presence of NaOH). The results allowed the overall activation energy to be estimated of the reaction of approximately 86 kJ mol^−1^ [282]:

(3) $$(-r_{\mathrm{gly}})\;=\frac{k_{f}{\;C}_{\mathrm{gly}}{\;C}_{\mathrm{OH}}{\;C}_{H2}^{2}}{C_{\mathrm{gly}}{\;C}_{\mathrm{OH}}{+K}_{H}{\;C}_{H2}{}^{3}}$$

LHHW-type models were implemented to describe the direct conversion of glycerol to 1,2-PG and then of 1,2-PG to 1-POH, using Ni-Cu/Al_2_O_3_ catalysts and formic acid as hydrogen-donor solvent. This model includes competitive adsorption for the active sites between glycerol and 1,2-PG. The equations for the rate of glycerol and 1,2-PG consumption are given by Equations (4) and (5), with K_gly_ and K_1,2-PG_ being the adsorption constants of glycerol and 1,2-PG, respectively [285]:

(4) $$(-r_{\mathrm{gly}})\;=\frac{k_{\mathrm{gly}}C_{\mathrm{gly}}}{{1+K}_{\mathrm{gly}}{\;C}_{\mathrm{gly}}{+K}_{1,2\mathrm{-PG}}{\;C}_{1,2\mathrm{-PG}}}$$

(5) $$r_{1,2\mathrm{-PG}}=\frac{k_{1,2\mathrm{-PG}}\;C_{1,2\mathrm{-PG}}}{{1+K}_{\mathrm{gly}}{\;C}_{\mathrm{gly}}{+K}_{1,2\mathrm{-PG}}{\;C}_{1,2\mathrm{-PG}}}\;$$

Tao et al. used a Ni-Raney catalyst to study the kinetics of hydrogenolysis and proposed LHHW type models to describe the formation of 1,2-PG, EG, and MeOH, considering one type of active site for the dissociative adsorption of H_2_ molecules and another type of site for the adsorption of glycerol and reaction products. The tests were carried out at 8 Mpa of H_2_ in the thermal range 180–220 °C with aqueous solutions containing 20 wt.% glycerol and 5 wt.% catalyst. The rate of glycerol consumption was expressed according to Equation (6) [284]:

(6) $$(-r_{\mathrm{gly}})\;=\frac{{(k}_{1,2\mathrm{-PG}}{+k}_{\mathrm{EG}}{)\;K}_{\mathrm{gly}}{\;C}_{\mathrm{gly}}}{{1+K}_{\mathrm{gly}}{\;C}_{\mathrm{gly}}{+K}_{1,2\mathrm{-PG}}{\;C}_{1,2\mathrm{-PG}}{+K}_{\mathrm{EG}}{\;C}_{\mathrm{EG}}{+K}_{\mathrm{MeOH}}{\;C}_{\mathrm{MeOH}}}\;$$

For a commercial Pt/C catalyst, a detailed model of the hydrogenolysis kinetics considering the formation of liquid and gas phase products was performed by Jin et al. Experimental tests employed a batch reactor in the thermal range of 130–160 °C, concentrations of glycerol between 2 and 40 wt.%, and a concentration of NaOH between 0.4 and 10 wt.%. The model implemented considered dehydrogenation, dehydration, hydrogenation, and C-C bond cleavage reactions that occurred in parallel and competed with each other for the same active sites. The results obtained show much lower activation energy (~53 kJ mol^−1^) than non-catalytic glycerol hydrothermal conversion (~128 kJ mol^−1^). For glycerol hydrogenolysis employing Pt/C, without external H_2_ addition (in N_2_ atmosphere), the results allowed an activation energy of ~64 kJ mol^−1^ to be estimated, which is low when compared to Pt/C catalysts in H_2_ atmospheres. It was also found that the hydrogenolysis of glycols and alcohols formed in the liquid phase to produce gases such as methane, ethane, and propane was restricted in the absence of a NaOH base in the reaction medium due to its high activation energy. The rate of glycerol consumption was expressed in terms of Equation (7) in combination with Equations (8)–(11) [279].

(7) $$(-r_{\mathrm{gly}}{)=w}_{\mathrm{cat}}{\;(r}_{1}{+r}_{2}{+r}_{4}{+r}_{5})\;$$

(8) $$r_{1}=\frac{k_{s1}K_{\mathrm{gly}}{\;C}_{\mathrm{gly}}{\;C}_{\mathrm{OH}}}{{{(1+K}_{\mathrm{gly}}{\;C}_{\mathrm{gly}}{+K}_{\mathrm{EG}}{\;C}_{\mathrm{EG}}{+K}_{\mathrm{OH}}{\;C}_{\mathrm{OH}})}^{2}}$$

(9) $$r_{2}=\frac{k_{s2}K_{\mathrm{gly}}{\;C}_{\mathrm{gly}}{\;C}_{\mathrm{OH}}}{{{(1+K}_{\mathrm{gly}}{\;C}_{\mathrm{gly}}{+K}_{\mathrm{EG}}{\;C}_{\mathrm{EG}}{+K}_{\mathrm{OH}}{\;C}_{\mathrm{OH}})}^{2}}$$

(10) $$r_{4}=\frac{k_{s4}K_{\mathrm{gly}}{\;C}_{\mathrm{gly}}{\;C}_{\mathrm{OH}}}{{{(1+K}_{\mathrm{gly}}{\;C}_{\mathrm{gly}}{+K}_{\mathrm{EG}}{\;C}_{\mathrm{EG}}{+K}_{\mathrm{OH}}{\;C}_{\mathrm{OH}})}^{2}}$$

(11) $$r_{5}=\frac{k_{s5}K_{\mathrm{gly}}{\;C}_{\mathrm{gly}}{\;C}_{\mathrm{OH}}}{{{(1+K}_{\mathrm{gly}}{\;C}_{\mathrm{gly}}{+K}_{\mathrm{EG}}{\;C}_{\mathrm{EG}}{+K}_{\mathrm{OH}}{\;C}_{\mathrm{OH}})}^{2}}$$

Employing a Cu-ZnO-Al_2_O_3_ catalyst prepared by the co-precipitation method with a Cu:Zn:Al = 1:1:0.5 ratio, Zhou et al. carried out kinetic experiments in an isothermal fixed-bed reactor in the range of 3–5 Mpa of H_2_ and temperatures between 220 and 240 °C. The kinetic data were fitted with a LHHW model considering two types of active sites, one for H_2_ adsorption in dissociative form and another for glycerol adsorption in competition with AcOH and 1,2-PG. With relative errors between 6% and 7%, the model allowed the activation energies to be estimated associated with the dehydration step (86.56 kJ mol^−1^) and hydrogenation (57.80 kJ mol^−1^), the former being the determining step in the reaction rate. The proposed expressions for glycerol dehydration and AcOH hydrogenation were presented in Equations (12) and (13) [175]:

(12) $$(-r_{\mathrm{gly}})\;=\frac{k_{1}K_{\mathrm{gly}}{\;C}_{\mathrm{gly}}}{{1+K}_{\mathrm{gly}}{\;C}_{\mathrm{gly}}{+K}_{\mathrm{AcOH}}{\;C}_{\mathrm{AcOH}}{+K}_{1,2\mathrm{-PG}}{\;C}_{1,2\mathrm{-PG}}}$$

(13) $$r_{\mathrm{AcOH}}=\frac{k_{2}K_{\mathrm{AcOH}}{\;C}_{\mathrm{AcOH}}{\;K}_{H2}{\;P}_{H2}}{{(1+K}_{\mathrm{gly}}C_{\mathrm{gly}}{+K}_{\mathrm{AcOH}}C_{\mathrm{AcOH}}{+K}_{1,2\mathrm{-PG}}C_{1,2\mathrm{-PG}})\;{\left(1+\sqrt{K_{H2}P_{H2}}\right)}^{2}}\;$$

Recently, Pandhare et al. [281] studied a Cu/MgO catalyst with 35 wt.% Cu in the range of 3–6 Mpa of H_2_, temperatures between 190 and 230 °C and glycerol concentrations between 20 and 60 wt.%. The kinetic data obtained were fitted using two models, one based on the power law and the other of the LHHW type. The LHHW model allowed the production rates to be obtained of 1,2-PG (Equation (14)) and EG (Equation (15)):

(14) $$r_{1,2\mathrm{-PG}}=\frac{k_{1,2\mathrm{-PG}}{\;C}_{\mathrm{gly}}{\;P}_{H2}}{{{(1+K}_{\mathrm{gly}}{\;C}_{\mathrm{gly}}{+K}_{H2}{\;P}_{H2}{+(C}_{1,2\mathrm{-PG}}{/K}_{1,2\mathrm{-PG}}{)+(C}_{\mathrm{EG}}{/K}_{\mathrm{EG}}))}^{2}\;}$$

(15) $$r_{\mathrm{EG}}=\frac{k_{\mathrm{EG}}{\;C}_{\mathrm{gly}}{\;P}_{H2}}{{{(1+K}_{\mathrm{gly}}{\;C}_{\mathrm{gly}}{+K}_{H2}{\;P}_{H2}{+(C}_{1,2\mathrm{-PG}}{/K}_{1,2\mathrm{-PG}}{)+(C}_{\mathrm{EG}}{/K}_{\mathrm{EG}}))}^{2}\;}\;$$

In another article [215], the authors employed a Cu-Ni/γ-Al_2_O_3_ catalyst for the production of 1,2-PG in vapor-phase and fitted the kinetic data with an Eley–Rideal model. The model allowed the calculation of the glycerol consumption rate (Equation (16)) and the 1,2-PG production rate (Equation (17)):

(16) $$(-r_{\mathrm{gly}})\;=\frac{k_{\mathrm{gly}}{\;K}_{\mathrm{gly}}{\;P}_{\mathrm{gly}}}{{1+K}_{\mathrm{gly}}{\;P}_{\mathrm{gly}}{+(P}_{\mathrm{AcOH}}{/K}_{\mathrm{AcOH}}{)+(P}_{1,2\mathrm{-PG}}{/K}_{1,2\mathrm{-PG}})\;}$$

(17) $$r_{1,2\mathrm{-PG}}=\frac{K_{1,2\mathrm{-PG}}{\;P}_{\mathrm{AcOH}}{\;P}_{H2}}{K_{\mathrm{AcOH}}{\;(1+K}_{\mathrm{gly}}{\;P}_{\mathrm{gly}}{+(P}_{\mathrm{AcOH}}{/K}_{\mathrm{AcOH}}{)+(P}_{1,2\mathrm{-PG}}{/K}_{1,2\mathrm{-PG}}))\;}\;$$

Furthermore, using the equation based on the Arrhenius law, the authors estimated the activation energies associated with the step of dehydration of glycerol to AcOH (55.14 kJ mol^−1^) and hydrogenation of AcOH to form 1,2-PG (50.87 kJ mol^−1^).

Regarding the Cu-Ni/γ-Al_2_O_3_ catalyst, Mondal et al. [280] studied its performance in a liquid phase batch reactor in the range of 180–220 °C, H_2_ partial pressures between 3 and 6 Mpa, and 20 wt.% glycerol solutions. The kinetic results were fitted considering a power-law based model and a LHHW type model. From the latter, they obtained the kinetic expression for glycerol consumption shown in Equation (18). In addition, they estimated the activation energies for the formation of 1,2-PG and 1-POH as 70.5 kJ mol^−1^ and 79.5 kJ mol^−1^, respectively.

(18) $$(-r_{\mathrm{gly}})\;=\frac{k_{\mathrm{gly}}{\;K}_{\mathrm{gly}}{\;P}_{H2}\;C_{\mathrm{gly}}}{{1+K}_{\mathrm{gly}}{\;P}_{\mathrm{gly}}{+(K}_{H2}{\;P}_{H2}{)}^{1/2}{+(C}_{1\text{-POH}}{/K}_{1\mathrm{-POH}}{)+(C}_{1,2\mathrm{-PG}}{/K}_{1,2\mathrm{-PG}})\;}\;$$

## 7. Conclusions, Perspectives and Future Work

The worldwide production of biodiesel generates a growing supply of crude glycerol that can be used as a raw material to obtain high value-added products that currently come from the petrochemical industry.

This work presents an exhaustive review of more than 300 scientific publications that have concentrated their efforts in studying the catalytic hydrogenolysis of glycerol for the production of 1,2-propylene glycol (1,2-PG). This compound is a glycol widely used in the chemical industry whose current synthesis is of petrochemical origin.

The hydrogenolysis of glycerol in the presence of catalysts to obtain 1,2-PG can be carried out in the liquid phase (2–10 MPa, 120–260 °C) using batch or continuous flow reactors (trickle bed) or in the vapor phase (0.1 MPa, 200–300 °C). Experimental results show that the highest yields for 1,2-PG are obtained in the liquid phase, and operating variables must be optimized to obtain high yields for 1,2-PG, such as temperature and pressure, the starting glycerol concentration, the glycerol/catalyst ratio (or space velocity) and eventually the use of solvents.

The analysis of the reaction mechanisms indicate that the formation of 1,2-PG depends on the acidity or basicity of the reaction medium and the catalyst employed. Acidic media favors the formation of 1,2-PG by the dehydration–hydrogenation mechanism, with AcOH as an intermediate product and the possible formation of EG, MeOH, 1-POH, 2-POH, EtOH, and gases. Basic media, on the other hand, promote the formation of 1,2-PG by the dehydrogenation–dehydration-hydrogenation mechanism, with the presence of GLA, 2-HA, PAL as intermediates and AL as a by-product, among others. In addition, the employment of certain group of catalysts, based on Ir, Rh, and Pt, in the presence of MOx (M = Re, V, Mo, W) leads to the 1,2-PG formation and its isomer through a direct hydrogenolysis mechanisms, including the formation of intermediates on the catalytic surface.

With respect to catalytic systems, in general the studies indicate that activity depends on the type of metal, its dispersion, and acid-base properties.

Regarding the metal, it has been found that catalysts based on noble metals (Ru, Pt, Pd), are active in the hydrogenolysis reaction, following the tendency Ru > Pt > Pd. However, the ability to cleave C-C bonds follows the same tendency, so the yield to 1,2-PG depends on the conversion and the selectivity values. In liquid phase, the following catalysts have been highlighted according to their performance at 1,2-PG:

PdO/Fe_2_O_3_ (94.0%) > Ru-Cu/Bentonite (86.4%) > Pt/HTL (85.6%)

The catalysts based on non-noble metals (Cu, Ni, Co) show a better ability to cleave C-O bonds so the intrinsic activity is higher for these catalytic systems, following the tendency Cu > Ni > Co. In the liquid phase, the following catalysts have been highlighted according to their performance at 1,2-PG:

Cu-Al_2_O_x_ (99.0%) > Ni/WO_3_-MSEP (85.4%) > Co-Al (70.1%)

Generally, favoring metal dispersion and increasing the number of the active sites of the support, lead to higher conversions, although those properties should be optimized so as not to generate side products. In this sense, it has been highlighted that the metal dispersion is a function of the precursor used for the preparation, the preparation method and, in the case of supported catalysts, the textural and acid-base properties of the supports.

Other properties, such as the acidity or basicity of the catalysts, favor the production of 1,2-PG by dehydration–hydrogenation or dehydrogenation–dehydrogenation–dehydrogenation–hydrogenation mechanisms, respectively. In this sense, a specific type of surface site and an adequate density of such sites are required to generate an optimal metal-surface site proximity that promotes the bifunctional effect.

A stability analysis indicates that traditional supports (Al_2_O_3_, MgO, SiO_2_) suffer alterations in the liquid phase due to the hydrothermal conditions of the reaction medium, and carbonaceous type supports (C, AC, CNT) or transition metal oxides (TiO_2_, Fe_3_O_4_) should be used. Catalysts based on noble metals (Pt, Ru, Pd) usually suffer deactivation associated with the adsorption of carbonaceous species and the formation of carbon, especially in the vapor phase, due to their ability to cleave C-C bonds. Catalysts based on non-noble metals (Cu, Ni, Co) are usually more susceptible to sintering and to oxidation and loss of the active phase by leaching. In addition, the use of crude glycerol as an alternative to technical or refined glycerol has adverse effects on the activity and selectivity of the catalysts regardless of their nature.

Kinetic models based on the power law and Langmuir–Hinshelwood–Hougen–Watson type have been developed, considering one or two types of active sites and, in some cases, the phenomenon of competition for these sites. The modeling results have allowed global activations energies for the hydrogenolysis reaction and for the dehydration and hydrogenation steps to be estimated, depending on the catalytic system employed.

From this review, it can be concluded that the hydrogenolysis of glycerol for the production of 1,2-PG remains a challenge for the scientific community. Although the use of crude glycerol solutions has been the subject of research in some publications, future work could contemplate the analysis of the different qualities of crude glycerol and the formulation of kinetic models that consider the effect of its impurities on the performance of these catalytic systems. The development of technical–economic and environmental impact studies could also be considered as fundamental tools for the analysis of the process in the framework of future glycerol biorefineries.

## Figures and Tables

**Table 1:** Hydrogenolysis to 1,2-PG in liquid phase employing batch reactors with Ru-supported catalysts.

Catalyst	T (°C)	P (MPa)	m_gly_/m_c_	t (h)	X (%)	S_1,2-PG_ (%)	Y_1,2-PG_ (%)	Ref.
Ru/KL	180	8.0 (H_2_)	22.8	24	7.5	32.0	2.4	[72]
Ru/HSAG	180	8.0 (H_2_)	22.8	24	26.0	16.0	4.1	[72]
Ru/WO_3_-ZrO_2_	180	8.0 (H_2_)	4.0	18	12.0	68.5	8.2	[73]
Ru/H-ZSM5	160	8.0 (H_2_)	29.4	8	20.5	42.2	8.6	[74]
Ru/AlF_3_-Al_2_O_3_	200	4.0 (H_2_)	25.3	4	48.3	23.7	11.4	[75]
Ru/La-ZrO_2_	190	6.0 (H_2_)	10.0	8	57.8	22.0	12.7	[76]
Ru/γ-Al_2_O_3_	200	1.4 (H_2_)	20.0	24	23.1	59.7	13.7	[45]
Ru/SiO_2_	240	8.0 (H_2_)	168.0	5	21.7	60.5	13.1	[77]
Ru/Hβ	160	8.0 (H_2_)	29.4	8	32.0	43.6	13.9	[74]
Ru/C	200	1.4 (H_2_)	20.0	24	43.7	40.0	17.4	[45]
Ru/ZrO_2_	180	8.0 (H_2_)	4.0	18	100.0	19.5	19.5	[73]
Ru/SiO_2_-C	200	2.0 (H_2_)	2.0	24	28.0	76.0	21.2	[78]
Ru/CsPW	150	0.5 (H_2_)	3.3	10	31.0	87.6	27.1	[79]
Ru/LaCO_3_OH	200	3.0 (H_2_)	4.0	3	62.0	46.0	28.5	[80]
Ru/TiO_2_	170	3.0 (H_2_)	10.3	12	66.3	47.7	31.6	[81]
Ru/CNT	200	4.0 (H_2_)	16.8	12	75.0	48.3	36.2	[82]
Ru/Bentonite	150	2.0 (H_2_)	20.0	7	72.5	66.9	48.5	[82]
Ru/HY	220	3.0 (H_2_)	6.0	10	81.3	60.1	48.8	[83]
Ru/CaMgZn-Al	180	2.5 (H_2_)	35.0	18	58.5	85.5	50.0	[84]
Ru/La_2_O_3_	190	6.0 (H_2_)	10.0	8	69.2	77.0	53.2	[76]
Ru/Bentonite-TiO_2_	150	2.0 (H_2_)	20.0	7	69.8	80.6	56.2	[85]
Ru/AC	240	5.2 (H_2_)	NR	5	92.5	70.0	64.8	[86]

NR: not reported; m_gly_/m_c_: glycerol to catalyst mass ratio; X: glycerol conversion; S_1,2-PG_: selectivity to 1,2-PG; Y_1,2-PG_: yield to 1,2-PG.

**Table 2:** Hydrogenolysis to 1,2-PG in liquid phase employing batch reactors with Ru bimetallic catalysts.

Catalyst	T (°C)	P (MPa)	m_gly_/m_c_	t (h)	X (%)	S_1,2-PG_ (%)	Y_1,2-PG_ (%)	Ref.
Ru-Au/C	200	4.0 (H_2_)	NR	5	25.0	30.0	7.5	[94]
Ru-Pt/C	200	4.0 (H_2_)	NR	5	42.0	24.0	10.0	[94]
Ru-Re/TiO_2_	160	8.0 (H_2_)	29.4	8	36.3	46.4	16.8	[74]
Ru-Re/HSZM-5	160	8.0 (H_2_)	29.4	8	54.2	41.5	22.4	[74]
Ru-Re/Hβ	160	8.0 (H_2_)	29.4	8	52.8	42.8	22.5	[74]
Ru-Re/SiO_2_	160	8.0 (H_2_)	29.4	8	51.7	44.8	23.1	[74]
Ru-Re/ZrO_2_	160	8.0 (H_2_)	29.4	8	56.9	47.2	26.8	[74]
Ru-Re/SiO_2_	125	7.5 (H_2_)	29.4	8	45.0	64.7	29.2	[98]
Ru-Co/TiO_2_	180	5.0 (H_2_)	63.1	12	72.8	41.7	30.3	[100]
Ru-Co/ZrO_2_	180	5.0 (H_2_)	12.6	10	56.2	70.3	39.5	[102]
Ru-Cu/Al_2_O_3_	200	2.5 (H_2_)	17.5	24	45.0	94.0	42.3	[104]
Ru-Fe/CNT	200	4.0 (H_2_)	16.8	24	100.0	50.0	50.0	[103]
Ru-Cu/TiO_2_	200	2.5 (H_2_)	17.5	12	53.9	93.2	50.2	[105]
Ru-Cu/ZrO_2_	180	8.0 (H_2_)	6.9	24	100.0	84.0	84.0	[106]
Ru-Cu/Bentonite	230	10.0 (H_2_)	5.4	18	100.0	86.4	86.4	[99]

NR: not reported; m_gly_/m_c_: glycerol to catalyst mass ratio; X: glycerol conversion; S_1,2-PG_: selectivity to 1,2-PG, Y_1,2-PG_: yield to 1,2-PG.

**Table 3:** Liquid phase hydrogenolysis to 1,2-PG employing batch reactors with Pt- supported catalysts.

Catalyst	T (°C)	P (MPa)	m_gly_/m_c_	t (h)	X (%)	S_1,2-PG_ (%)	Y_1,2-PG_ (%)	Ref.
Pt/Hβ	220	3.0 (H_2_)	8.0	20	7.0	9.5	0.6	[109]
Pt/H-ZSM5	220	3.0 (H_2_)	8.0	20	4.0	19.5	0.7	[109]
Pt/Al_2_O_3_-SiO_2_	210	6.0 (H_2_)	0.45	6	10.0	61.0	6.1	[110]
Pt/ZnO	180	0.6 (H_2_)	12.5	15	28.0	32.0	8.9	[111]
Pt/SiO_2_-Al_2_O_3_	220	4.5 (H_2_)	0.006	24	90.0	11.2	10.0	[112]
Pt/CaCO_3_	200	4.0 (H_2_)	20.0	18	45.0	22.5	10.1	[113]
Pt/TiO_2_	210	6.0 (H_2_)	0.45	6	23.0	49.0	11.2	[110]
Pt/CeO_2_	180	0.6 (H_2_)	12.5	15	33.0	35.0	11.5	[111]
Pt/La_2_O_3_	180	0.6 (H_2_)	12.5	15	33.0	38.0	12.5	[111]
Pt/Al_2_O_3_	220	8.0 (H_2_)	17.7	120	60.0	45.0	27.0	[114]
	220	3.0 (H_2_)	8.0	20	39.0	81.2	31.6	[109]
Pt/WCx	220	4.0 (H_2_)	28.0	8	79.2	39.3	31.1	[115]
Pt/MgO	220	3.0 (H_2_)	8.0	20	50.0	81.2	40.6	[109]
Pt/C	160	4.0 (H_2_)	15.6	4	57.0	73.0	41.6	[116]
Al/Pt/Al_2_O_3_	220	8.0 (H_2_)	17.7	120	90.0	64.0	57.6	[114]
Pt/Fe_3_O_4_	230	2.0 (H_2_)	30.0	16	81.0	79.3	64.2	[117]
Pt/Nb_2_O_5_-Al_2_O_3_	220	5.0 (H_2_)	28.0	10	78.0	95.0	74.1	[118]
Pt/HTL	220	3.0 (H_2_)	8.0	20	92.1	93.0	85.6	[109]

m_gly_/m_c_: glycerol to catalyst mass ratio; X: glycerol conversion; S_1,2-PG_: selectivity to 1,2-PG, Y_1,2-PG_: yield to 1,2-PG.

**Table 4:** Liquid-phase 1,2-PG hydrogenolysis employing batch reactors with bimetallic Pt catalysts.

Catalyst	T (°C)	P (MPa)	m_gly_/m_c_	t (h)	X (%)	S_1,2-PG_ (%)	Y_1,2-PG_ (%)	Ref.
Pt-Re/C	170	4.0 (H_2_)	NR	5	50.0	36.0	18.0	[122]
Pt-Fe/γ-Al_2_O_3_	220	2.6 (H_2_)	63.1	12	33.5	61.2	20.5	[104]
Pt-Re/CNT	170	4.0 (H_2_)	3.7	8	51.2	55.0	28.2	[123]
Pt-Sn/SiO_2_	200	0.4 (N_2_)	4.1	2	54.0	59.0	31.8	[125]
Pt-Ir-ReOx/SiO_2_	190	2.0 (Ar)	20.0	17	78.0	42.0	32.7	[126]
Pt-Ni/γ-Al_2_O_3_	240	1.0 (N_2_)	21.0	3	71.4	52.4	37.4	[121]
Pt-Ni/α-Al_2_O_3_-CeO_2_-ZrO_2_	250	1.2 (N_2_)	2.5	2	61.0	70.0	42.7	[120]
Pt-Au/TiO_2_	150	0.7 (H_2_)	4.7	16	68.0	90.0	61.2	[124]

NR: not reported; m_gly_/m_c_: glycerol to catalyst mass ratio; X: glycerol conversion; S_1,2-PG_: selectivity to 1,2-PG, Y_1,2-PG_: yield to 1,2-PG.

**Table 5:** Liquid phase 1,2-PG hydrogenolysis using batch reactors with Pd catalysts.

Catalyst	T (°C)	P (MPa)	m_gly_/m_c_	t (h)	X (%)	S_1,2-PG_ (%)	Y_1,2-PG_ (%)	Ref.
Pd/SiO_2_	200	5.0 (H_2_)	20	10	2.4	65.3	1.6	[127]
Pd/C	200	1.4 (H_2_)	20	24	11.2	57.1	6.4	[45]
Pd/ZrO_2_	200	6.0 (H_2_)	14.2	3	21.0	90.5	19.0	[87]
Pd/bentonite	150	2.0 (H_2_)	4.2	7	29.0	73.2	21.2	[128]
Pd/Fe_2_O_3_	200	5.0 (H_2_)	1.5	12	37.5	95.0	35.6	[129]
Pd/Fe_3_O_4_	180	0.5 (N_2_)	6.0	24	100.0	55.9	55.9	[130]
PdO/CoO	180	0.5 (H_2_)	5.0	24	100.0	60.0	60.0	[131]
Pd/Co_3_O_4_	180	0.5 (N_2_)	6.0	24	100.0	64.0	64.0	[130]
Pd/Fe_2_O_3_	180	0.5 (H_2_)	5.0	24	100.0	71.2	71.2	[131]
PdO/Fe_2_O_3_	180	0.5 (N_2_)	5.0	24	100.0	94.0	94.0	[132]

m_gly_/m_c_: glycerol to catalyst mass ratio; X: glycerol conversion; S_1,2-PG_: selectivity to 1,2-PG, Y_1,2-PG_: yield to 1,2-PG.

**Table 6:** Liquid phase 1,2-PG hydrogenolysis employing batch reactors with bimetallic Pd catalysts.

Catalyst	T (°C)	P (MPa)	m_gly_/m_c_	t (h)	X (%)	S_1,2-PG_ (%)	Y_1,2-PG_ (%)	Ref.
Pd-Re/SBA-15	200	8.0 (H_2_)	29.4	18	45.1	60.4	27.2	[133]
	200	8.0 (H_2_)	29.4	18	72.2	41.6	30.0	[135]
Pd-Zn/ZrO_2_	220	6.0 (H_2_)	50.0	4	40.0	91.5	36.6	[138]
Pd-Ni	220	6.0 (H_2_)	21.0	12	63.5	59.4	37.7	[140]
Pd-Zn/ZnO-Al	230	3.0 (H_2_)	52.6	6	70.1	92.6	64.9	[139]
Pd-Cu/Mg_5,6-x_Al_2_O_8,6-x_	200	2.0 (H_2_)	6.0	10	95.0	96.1	91.2	[136]
Pd-CuCr_2_O_4_	220	4.0 (H_2_)	50.0	18	100.0	93.9	93.9	[137]

m_gly_/m_c_ = glycerol to catalyst mass ratio; X: glycerol conversion; S_1,2-PG_: selectivity to 1,2-PG, Y_1,2-PG_: yield to 1,2-PG.

**Table 7:** Hydrogenolysis to 1,2-PG in liquid phase batch reactors using Cu bulk catalysts.

Catalyst	T (°C)	P (MPa)	m_gly_/m_c_	t (h)	X (%)	S_1,2-PG_ (%)	Y_1,2-PG_ (%)	Ref.
Cu-ZrO_2_	200	4.0 (H_2_)	16.7	8	10.0	90.0	9.0	[141]
CuCr_2_O_4_-Ba	220	5.2 (H_2_)	20.0	5	34.0	85.0	28.9	[142]
Cu-Ca-Al	200	3.5 (H_2_)	16.7	8	73.2	42.6	31.1	[143]
CuO/ZnO	200	5.0 (H_2_)	58.8	7	46.0	90.0	41.4	[144]
Cu-Fe	190	4.1 (H_2_)	20.0	10	47.0	92.0	43.2	[145]
CuCr_2_O_4_	200	1.4 (H_2_)	20.0	24	54.8	85.0	46.5	[45]
Cu-Cr	130	2.0 (H_2_)	20.0	4	52.4	99.6	52.2	[146]
Cu-Al	220	7.0 (H_2_)	19.3	24	74.2	78.8	58.4	[147]
Cu-ZrO_2_-MgO	180	4.0 (H_2_)	16.7	8	61.7	96.5	59.5	[148]
Cu-Raney-MgO	180	1.0 (H_2_)	41.7	24	75.0	85.0	63.7	[149]
Cu/Cr	220	8.0 (H_2_)	45.0	12	80.3	83.9	67.4	[150]
CuO/SiO_2_	200	9.0 (H_2_)	16.0	12	73.4	94.0	69.0	[151]
Cu-ZnO	200	6.0 (H_2_)	4.5	6	75.0	93.0	69.8	[152]
Cu/MgO	180	3.0 (H_2_)	7.1	20	72.0	97.6	70.2	[153]
Cu/Al_2_O_3_	200	4.0 (H_2_)	16.7	24	75.7	95.8	72.5	[154]
Cu_1_._5_-Ca_2_AlO_3_._5_	180	2.0 (H_2_)	8.0	21	75.3	98.1	73.8	[155]
Cu-Zn-Al	230	7.0 (H_2_)	20.0	4	96.1	81.4	78.2	[156]
Cu-Raney	240	3.0 (H_2_)	3.0	7	93.0	86.0	79.0	[41]
Cu/ZnO/Ga_2_O_3_	220	5.0 (H_2_)	25.3	5.5	99.0	80.0	79.2	[157]
CuAl_2_O_4_	220	5.0 (H_2_)	50.0	12	91.0	91.0	82.9	[158]
Cu_0_._4_/Zn_5_._6-x_Mg_x_Al_2_O_8_._6_	200	2.0 (H_2_)	15.0	10	85.5	98.6	84.3	[159]
Cu/ZnO/Al_2_O_3_	200	2.7 (H_2_)	20.0	24	92.3	94.5	87.2	[160]
Cu-Zn-Mg-Al-O	210	4.5 (H_2_)	13.3	12	98.3	91.5	89.9	[161]
Cu-Zn-Cr-Zr	240	4.0 (H_2_)	26.7	10	100.0	97.0	97.0	[162]
Cu-Al_2_O_x_	180	10 (H_2_)	1.3	6	100.0	99.0	99.0	[163]

m_gly_/m_c_ = glycerol to catalyst mass ratio; X: glycerol conversion; S_1,2-PG_: selectivity to 1,2-PG, Y_1,2-PG_: yield to 1,2-PG.

**Table 8:** Hydrogenolysis to 1,2-PG in batch reactors using Cu-supported catalysts.

Catalyst	T (°C)	P (MPa)	m_gly_/m_c_	t (h)	X (%)	S_1,2-PG_ (%)	Y_1,2-PG_ (%)	Ref.
Cu/TiO_2_	200	4.0 (H_2_)	9.4	24	49.0	20.0	9.8	[178]
Cu/Cr_2_O_3_	200	5.0 (H_2_)	20.0	10	15.1	73.8	11.1	[127]
Cu-MgO/USY	200	3.5 (H_2_)	16.7	10	57.0	29.1	16.5	[179]
Cu/HSM	240	8.0 (H_2_)	16.6	5	43.0	91.1	39.1	[180]
Cu/SBA-15	240	8.0 (H_2_)	16.6	5	52.0	96.2	50.0	[180]
Cu/γ-Al_2_O_3_	210	4.5 (H_2_)	10.5	12	59.3	86.6	51.3	[181]
Cu/ZnO-USY	220	3.5 (H_2_)	16.6	5	95.1	58.4	55.5	[182]
Cu/DUSY	200	3.5 (H_2_)	16.6	10	78.7	98.6	77.3	[183]
Cu_0_._4_/Mg_5_._6_Al_2_O_8_._6_	180	3.0 (H_2_)	7.1	20	80.0	98.2	78.5	[184]
Cu/MgO	210	4.5 (H_2_)	12.5	12	96.6	92.6	89.4	[185]
Cu/SiO_2_	200	3.4 (H_2_)	2.5	10	98.4	98.1	96.5	[186]

m_gly_/m_c_; glycerol to catalyst mass ratio; X: glycerol conversion; S_1,2-PG_: selectivity to 1,2-PG; Y_1,2-PG_: yield to 1,2-PG.

**Table 9:** Hydrogenolysis to 1,2-PG in liquid-phase continuous flow reactors using Cu-supported catalysts.

Catalyst	T (°C)	P (MPa)	SV (h^−1^)	X (%)	S_1,2-PG_ (%)	Y_1,2-PG_ (%)	Ref.
Cu/HZSM-5	250	4.0 (H_2_)	2.0 (WHSV)	40.0	75.0	30.0	[187]
Cu/TALCITE	190	2.5 (H_2_)	NR (WHSV)	65.0	98.3	63.8	[188]
Cu/γ-Al_2_O_3_	250	4.0 (H_2_)	2.0 (WHSV)	75.0	93.3	70.0	[187]
Cu-H_4_SiW_12_O_40_/γ-Al_2_O_3_	240	6.0 (H_2_)	0.9 (WHSV)	90.1	89.7	80.8	[189]
Cu/SiO_2_	250	4.0 (H_2_)	0.8 (WHSV)	99.6	86.4	86.0	[190]
Cu/SBA-15	250	4.0 (H_2_)	0.8 (WHSV)	96.0	92.4	88.7	[190]
Cu-B_2_O_3_/Al_2_O_3_	250	6.0 (H_2_)	0.1 (WHSV)	98.0	98.0	96.0	[191]
Cu/SiO_2_	200	5.0 (H_2_)	0.07 (WHSV)	100.0	98.3	98.3	[192]

SV: space velocity; NR: not reported; m_gly_/m_c_: glycerol to catalyst mass ratio; X: glycerol conversion; S_1,2-PG_: selectivity to 1,2-PG; Y_1,2-PG_: yield to 1,2-PG.

**Table 10:** Hydrogenolysis to 1,2-PG in batch reactors using Cu bimetallic catalysts.

Catalyst	T (°C)	P (MPa)	m_gly_/m_c_	t (h)	X (%)	S_1,2-PG_ (%)	Y_1,2-PG_ (%)	Ref.
Cu-Ag/γ-Al_2_O_3_	200	3.5 (H_2_)	30.0	8	66.4	68.2	45.2	[207]
Cu-Ni/γ-Al_2_O_3_	220	4.0 (H_2_)	40.0	24	92.5	62.5	57.8	[209]
Cu-Ru/CNT	200	4.0 (H_2_)	20.0	6	99.8	86.5	86.3	[206]
Cu-Zn/MgO	210	4.5 (H_2_)	12.5	12	98.7	93.4	92.2	[203]

m_gly_/m_c_: glycerol to catalyst mass ratio; X: glycerol conversion; S_1,2-PG_: selectivity to 1,2-PG, Y_1,2-PG_: yield to 1,2-PG.

**Table 11:** Hydrogenolysis to 1,2-PG in continuous flow reactors in vapor phase using Cu-supported catalysts.

Catalyst	T (°C)	P (MPa)	SV (h^−1^)	X (%)	S_1,2-PG_ (%)	Y_1,2-PG_ (%)	Ref.
Cu/ZnO/ZrO_2_	240	0.1 (H_2_)	0.84 (LHSV)	100.0	12.0	12.0	[211]
Cu/ZnO/TiO_2_	240	0.1 (H_2_)	0.84 (LHSV)	100.0	15.0	15.0	[211]
Cu/SBA-15	220	0.1 (H_2_)	1.03 (WHSV)	90.0	84.0	75.6	[200]
CuO/Y	210	0.2 (H_2_)	1.04 (WHSV)	92.0	83.0	76.4	[212]
Cu/Cr_2_O_3_	200	0.1 (H_2_)	0.75 (LHSV)	100.0	77.3	77.3	[213]
Cu/SiO_2_	255	1.5 (H_2_)	5.5 (WHSV)	100.0	87.0	87.0	[197]
Cu-Zn/MgO	220	0.72 (H_2_)	0.07 (WHSV)	98.5	89.0	87.6	[214]
Cu/ZnO/Al_2_O_3_	190	6.4 (H_2_)	0.08 (WHSV)	96.2	92.2	88.6	[127]
Cu-Ni/γ-Al_2_O_3_	220	0.75 (H_2_)	1.3 (WHSV)	100.0	89.5	89.5	[215]
Cu/MgO	220	0.75 (H_2_)	1.2 (WHSV)	100.0	95.5	95.5	[216]
Cu/γ-Al_2_O_3_	230	1.4 (H_2_)	1.9 (LHSV)	100.0	96.8	96.8	[217]
Cu-Ag/Al_2_O_3_	170–105	0.1 (H_2_)	0.16 (WHSV)	100.0	98.5	98.5	[218]

SV: space velocity; NR: not reported, m_gly_/m_c_: glycerol to catalyst mass ratio; X: glycerol conversion; S_1,2-PG_: selectivity to 1,2-PG; Y_1,2-PG_: yield to 1,2-PG.

**Table 12:** Hydrogenolysis to 1,2-PG in liquid phase batch reactors using Ni bulk catalysts.

Catalyst	T (°C)	P (MPa)	m_gly_/m_c_	t (h)	X (%)	S_1,2-PG_ (%)	Y_1,2-PG_ (%)	Ref.
Ni/Mg/Al	200	1.4 (H_2_)	20.0	24	8.7	24.4	2.1	[172]
Ni/Co/Mg/Al	200	1.4 (H_2_)	20.0	24	9.2	36.2	3.3	[172]
Ni_3_P	190	5.5 (H_2_)	100.0	4	5.0	86.4	4.3	[221]
Ni-Raney	230	4.0 (H_2_)	6.7	9	80.0	23.0	18.4	[222]
	200	1.4 (H_2_)	20.0	24	49.5	52.7	26.1	[45]
Ni-Raney-Ag	210	4.0 (H_2_)	8.0	6	78.0	65.9	51.4	[223]
ZnNiAl	230	3.0 (H_2_)	10.5	5	100.0	54.2	54.2	[224]
Ni-Raney	190	1.0 (H_2_)	4.0	44	97.0	71.0	68.8	[51]

m_gly_/m_c_: glycerol to catalyst mass ratio; X: glycerol conversion; S_1,2-PG_: selectivity to 1,2-PG; Y_1,2-PG_: yield to 1,2-PG.

**Table 13:** Hydrogenolysis to 1,2-PG in liquid phase batch reactors using Ni supported catalysts.

Catalyst	T (°C)	P (MPa)	m_gly_/m_c_	t (h)	X (%)	S_1,2-PG_ (%)	Y_1,2-PG_ (%)	Ref.
Ni/SiO_2_	200	6.0 (H_2_)	20.0	10	56.9	44.4	25.2	[225]
Ni/C	200	1.4 (H_2_)	20.0	24	39.8	68.6	27.3	[45]
Ni/CeO_2_	215	4.1 (H_2_)	12.0	24	69.9	39.5	27.6	[226]
Ni/SiO_2_-Al_2_O_3_	200	2.5 (H_2_)	20.0	8	30.0	98.0	29.4	[227]
Ni/Ce-Mg	230	6.9 (H_2_)	6.0	24	68.8	47.5	32.6	[226]
Ni/γ-Al_2_O_3_	200	6.0 (H_2_)	20.0	10	97.1	44.2	42.9	[225]
Ni/SiO_2_-C	260	2.0 (H_2_)	6.2	6	56.0	77.3	43.3	[228]
Ni/AC	200	5.0 (H_2_)	5.3	24	63.2	77.4	48.9	[229]
Ni/NaX	200	6.0 (H_2_)	20.0	10	86.6	80.4	69.6	[225]
Ni/γ-Al_2_O_3_	220	2.0 (H_2_)	6.2	5	100.0	83.0	83.0	[230]
Ni/WO_3_-MSEP	180	2.0 (H_2_)	13.3	6	88.3	96.8	85.4	[231]

m_gly_/m_c_: glycerol to catalyst mass ratio; X: glycerol conversion; S_1,2-PG_: selectivity to 1,2-PG, Y_1,2-PG_: yield to 1,2-PG.

**Table 14:** Hydrogenolysis to 1,2-PG in liquid phase batch reactors using Ni bimetallic catalysts.

Catalyst	T (°C)	P (Mpa)	m_gly_/m_c_	t (h)	X (%)	S_1,2-PG_ (%)	Y_1,2-PG_ (%)	Ref.
Ni-Ce/SBA-15	200	2.4 (H_2_)	33.3	8	51.0	29.0	14.7	[239]
Ni-Ir/γ-Al_2_O_3_	200	2.5 (H_2_)	6.6	12	24.3	83.1	20.2	[241]
Ni-Zn/SiO_2_-C	260	2.0 (H_2_)	4.2	2	55.7	86.7	48.3	[242]
Ni-Ce/AC	200	5.0 (H_2_)	0.04	6	90.4	65.7	59.3	[243]
Ni-Cu/γ-Al_2_O_3_	210	4.5 (H_2_)	10.5	12	71.6	93.0	66.6	[181]
	220	4.5 (N_2_)	10.9	24	90.0	82.0	73.8	[235]
Ni-P/γ-Al_2_O_3_	230	3.0 (H_2_)	13.9	15	95.7	84.7	81.0	[244]

m_gly_/m_c_: glycerol to catalyst mass ratio; X: glycerol conversion; S_1,2-PG_: selectivity to 1,2-PG; Y_1,2-PG_: yield to 1,2-PG.

**Table 15:** Hydrogenolysis to 1,2-PG in batch reactors using Co bulk catalysts.

Catalyst	T (°C)	P (MPa)	m_gly_/m_c_	t (h)	X (%)	S_1,2-PG_ (%)	Y_1,2-PG_ (%)	Ref.
Co nanocomposite	220	6.0 (H_2_)	40.0	8	33.5	47.6	15.9	[246]
Co-Cu nanocomposite	220	3.0 (H_2_)	80.0	7	40.3	60.8	24.5	[247]
Co nanostructures	220	5.2 (H_2_)	80.0	7	35.0	72.0	25.2	[248]
Co-Ni	220	3.0 (H_2_)	80.0	7	71.1	48.2	34.2	[249]
	220	3.0 (H_2_)	80.0	7	61.0	63.0	38.4	[250]
Co-ZnO	180	4.0 (H_2_)	6.7	8	70.0	80.0	56.0	[251]
Co-Al	200	4.0 (H_2_)	3.0	15	100.0	70.1	70.1	[252]

m_gly_/m_c_: glycerol to catalyst mass ratio; X: glycerol conversion; S_1,2-PG_: selectivity to 1,2-PG, Y_1,2-PG_: yield to 1,2-PG.

**Table 16:** Hydrogenolysis to 1,2-PG in liquid phase continuous flow reactors using Co bulk catalysts.

Catalyst	T (°C)	P (MPa)	SV (h^−1^)	X (%)	S_1,2-PG_ (%)	Y_1,2-PG_ (%)	Ref.
Co-Al	230	3.5 (H_2_)	2.6 (WHSV)	48.7	77.2	37.6	[253]
Co-Al-H_4_[Si(W_3_O_10_)_4_	230	3.5 (H_2_)	2.6 (WHSV)	76.3	60.2	45.9	[253]

SV: space velocity, X: glycerol conversion, S_1,2-PG_: selectivity to 1,2-PG, Y_1,2-PG_: yield to 1,2-PG.

**Table 17:** Hydrogenolysis to 1,2-PG in liquid phase batch reactors using Co-supported catalysts.

Catalyst	T (°C)	P (MPa)	m_gly_/m_c_	t (h)	X (%)	S_1,2-PG_ (%)	Y_1,2-PG_ (%)	Ref.
Co/SiO_2_	200	5.0 (H_2_)	20.0	10	8.3	61.7	5.1	[127]
Co/MgO	200	2.0 (H_2_)	20.0	9	44.8	42.2	18.9	[254]
Co/ZnAl	200	2.0 (H_2_)	13.3	12	70.6	57.8	40.8	[255]

m_gly_/m_c_: glycerol to catalyst mass ratio; X: glycerol conversion; S_1,2-PG_: selectivity to 1,2-PG, Y_1,2-PG_: yield to 1,2-PG.

**Table 18:** Effect of glycerol concentration in liquid phase using batch reactors.

Catalyst	Range (wt.%)	C_gly_ (wt.%)	T (°C)	P (MPa)	m_gly_/m_c_	t (h)	Y_1,2-PG_ (%)	Ref.
Ru/C	2–40	20	120	8.0 (H_2_)	2.7	10	0.9	[52]
Rh/SiO_2_	2–40	20	120	8.0 (H_2_)	2.7	10	2.7	[52]
Ru/C + Nb_2_O_5_	20–80	20	180	6.0 (H_2_)	55.5	8	28.9	[261]
Ru/C + Amberlyst 15	20–100	20	130	8.0 (H_2_)	20	24	36.4	[262]
Cu-MgO	10–80	20	200	4.0 (H_2_)	16.7	8	45.5	[167]
Co-ZnO	10–40	20	180	4.0 (H_2_)	6.7	8	56.0	[251]
Ni-Ce/AC	25–75	25	200	5.0 (H_2_)	5.4	6	58.9	[243]
Cu-ZrO_2_-MgO	10–40	20	180	4.0 (H_2_)	16.7	8	59.5	[152]
Cu/MgO	10–80	20	210	4.5 (H_2_)	12.5	12	89.4	[185]

m_gly_/m_c_: glycerol to catalyst mass ratio; X: glycerol conversion; S_1,2-PG_: selectivity to 1,2-PG; Y_1,2-PG_: yield to 1,2-PG.

**Table 19:** Effect of glycerol concentration in vapor phase using continuous flow reactors.

Catalyst	Range (wt.%)	C_gly_ (wt.%)	T (°C)	P (MPa)	SV (h^−1^)	Y_1,2-PG_ (%)	Ref.
Ru/SBA-15	40–60	40	260	0.1 (H_2_)	2.52 (WHSV)	15.0	[107]
Ni-Ag/γ-Al_2_O_3_	10–40	20	200	0.1 (H_2_)	2.01 (WHSV)	46.4	[238]
Cu/SBA-15	20–60	20	220	0.1 (H_2_)	1.03 (WHSV)	75.6	[200]

SV: space velocity; Y_1,2-PG_: yield to 1,2-PG; C_gly_: glycerol concentration that maximizes the yield to 1,2-PG.

**Table 20:** Thermal effect studied in batch reactors using different catalysts.

Catalyst	Range (°C)	T_optimum_ (°C)	P (MPa)	m_gly/_mc	t (h)	Y_1,2-PG_ (%)	Ref.
Rh/SiO_2_	120–160	160	8.0 (H_2_)	28.0	10	7.0	[52]
Ru/C	120–160	160	8.0 (H_2_)	28.0	10	11.0	[52]
Ru/ZrO_2_	180–240	210	6.0 (H_2_)	1.4	3	11.3	[87]
Co/MgO	180–250	200	2.0 (H_2_)	20.0	9	18.9	[254]
Pt/ZrO_2_	180–240	180	6.0 (H_2_)	1.4	3	18.9	[87]
Ru/Al_2_O_3_ + Pt/Al_2_O_3_	200–250	220	1.4 (N_2_)	12.0	6	23.6	[95]
CoCu nanocomposite	190–250	220	3.0 (H_2_)	80.0	7	24.5	[247]
Pd-Ni	160–260	220	2.0 (H_2_)	21.0	12	25.6	[140]
Ru/CsPW	120–200	150	0.5 (H_2_)	3.5	10	27.1	[79]
Ru/Al_2_O_3_	120–180	180	8.0 (H_2_)	29.4	8	28.0	[88]
Ru-Re/SiO_2_	110–160	125	7.5 (H_2_)	29.4	8	29.2	[98]
Ni/SiO_2_-Al_2_O_3_	170–250	200	2.5 (H_2_)	20.0	8	29.4	[227]
Cu-Ca-Al	160–230	200	3.5 (H_2_)	16.6	8	31.1	[143]
Ni/Ce-Mg	200–230	230	6.9 (H_2_)	6.0	24	32.7	[237]
Cu-ZnO	180–240	220	4.2 (H_2_)	13.8	12	33.0	[70]
	120–220	200	2.0 (H_2_)	16.6	16	34.0	[152]
Pt-Ni/γ-Al_2_O_3_	220–260	240	1.0 (N_2_)	21.0	3	37.4	[121]
Rh-ReOx/SiO_2_	100–180	160	8.0 (H_2_)	28.0	2	39.0	[59]
Ru-Co/ZrO_2_	140–200	180	5.0 (H_2_)	12.6	10	39.5	[102]
Ru-Cu/Al_2_O_3_	180–220	200	2.5 (H_2_)	17.5	24	42.3	[104]
Cu-Ag/γ-Al_2_O_3_	180–240	200	3.5 (H_2_)	30.0	8	45.2	[203]
Cu-MgO	160–220	200	4.0 (H_2_)	17.5	8	45.5	[167]
Cu/Al_2_O_3_	180–240	220	3.6 (H_2_)	8.5	10	47.5	[276]
Ru/HY	190–220	220	4.0 (H_2_)	6.0	10	48.8	[83]
Cu-Ni/γ-Al_2_O_3_	190–230	210	4.5 (H_2_)	10.5	12	51.3	[181]
Cu/CeO_2_/MgO	160–220	200	6.0 (H_2_)	10.0	10	54.9	[277]
Ru/Bentonite-TiO_2_	110–170	150	2.0 (H_2_)	20.0	7	56.2	[85]
Cu-ZrO2-MgO	160–220	180	4.0 (H_2_)	16.7	8	59.5	[251]
Pt/Fe_3_O_4_	190–240	230	2.0 (H_2_)	30.0	16	64.2	[117]
CuO/SiO_2_	160–220	200	9.0 (H_2_)	16.0	12	69.0	[151]
Cu/Al_2_O_3_	150–230	200	4.0 (H_2_)	20.0	24	72.5	[154]
Cu/DUSY	160–240	200	3.5 (H_2_)	16.6	10	77.6	[183]
Ni/WO3-MSEP	120–220	180	2.0 (H_2_)	10.5	6	83.9	[231]
Cu_0_._4_/Zn_5_._6-x_Mg_x_Al_2_O_8_._6_	160–200	200	2.0 (H_2_)	15.0	10	84.3	[159]
Cu/SiO_2_	190–220	200	6.0 (H_2_)	14.7	8	85.8	[204]
Ru-Cu/Bentonite	190–240	230	10 (H_2_)	5.4	18	86.4	[99]
Cu/MgO	190–230	210	4.5 (H_2_)	12.5	12	89.4	[185]
Pd-Cu/Mg_5,6-x_Al_2_O_8,6-x_	120–200	200	2.0 (H_2_)	6.0	10	91.2	[136]
Pd-Cu/KF-γ-Al_2_O_3_	150–220	200	2.5 (H_2_)	11.1	20	95.5	[211]
Cu/SiO_2_	180–220	200	3.4 (H_2_)	2.5	10	96.5	[186]

m_gly_/m_c_: glycerol to catalyst mass ratio; Y_1,2-PG_: yield to 1,2-PG.

**Table 21:** Thermal effect studied in continuous flow reactors using different catalysts.

Catalyst	Range (°C)	T_optimum_ (°C)	P (MPa)	SV (h^−1^)	Y_1,2-PG_ (%)	Ref.
Cu/ZnO/ZrO_2_	240–300	240	0.1 (H_2_)	0.84 (LHSV)	12.0	[211]
Cu/ZnO/TiO_2_	240–300	240	0.1 (H_2_)	0.84 (LHSV)	15.0	[211]
CuCr_2_O_4_	200–240	220	0.1 (H_2_)	0.10 (WHSV)	42.4	[260]
Ni-Ag/γ-Al_2_O_3_	180–260	200	0.1 (H_2_)	2.00 (WHSV)	46.4	[238]
CuO/Y	190–250	210	0.2 (H_2_)	1.00 (WHSV)	76.4	[212]
Cu/MgO	180–280	220	0.7 (H_2_)	1.20 (WHSV)	95.5	[216]

SV: space velocity; Y_1,2-PG_: yield to 1,2-PG.

**Table 22:** Activation energies of the hydrogenolysis reaction of glycerol in liquid phase using different catalysts.

Catalyst	Range (°C)	E_a_ (kJ mol^−1^)	Ref.
Cu-ZrO_2_-MgO	160–220	22.7	[148]
Cu/CeO_2_/MgO	160–220	26.6	[277]
Co-ZnO	160–220	31.0	[251]
CuO/SiO_2_	160–220	48.0	[151]
Ru-Re/C	220–240	54.2	[278]
Cu/MgO	160–220	58.3	[277]
Pt/Fe_3_O_4_	190–240	61.1	[117]
Pt/C	130–160	63.7	[279]
Cu-Ni/γ-Al_2_O_3_	180–220	67.7	[280]
Pd-Cu/Mg_5,6-x_Al_2_O_8,6-X_	150–180	77.1	[136]
Cu/MgO	190–230	78.5	[216]
Cu/MgO	190–230	84.9	[281]
Co-Pd-Re/C	180–202	86.0	[282]
Cu/SiO_2_	180–240	96.8	[268]
Rh/C	180–240	98.0	[58]
Cu/ZrO_2_	175–225	106.0	[283]
Ru-Re/SiO_2_	110–130	107.8	[98]
Cu-Zn-Cr-Zr	220–250	137.2	[162]

**Table 23:** Effect of pressure in batch reactors using different catalysts.

Catalyst	Range (MPa)	P_optimum_ (MPa)	T (°C)	m_gly_/m_c_	t (h)	Y_1,2-PG_ (%)	Ref.
Ru/TiO_2_	2.0–8.0 (H_2_)	6.0 (H_2_)	180	16.7	8	28.9	[90]
Cu-Ca-Al	2.0–4.0 (H_2_)	3.5 (H_2_)	200	16.7	8	31.1	[143]
Ni/Ce-Mg	6.9–8.6 (H_2_)	8.6 (H_2_)	215	4.0	24	33.0	[237]
Pd-Ni	2.0–10.0 (H_2_)	6.0 (H_2_)	220	21.0	12	37.7	[121]
Ru-Co/ZrO_2_	2.0–6.0 (H_2_)	5.0 (H_2_)	180	12.6	10	39.5	[102]
Cu-Ag/γ-Al_2_O_3_	2.5–4.5 (H_2_)	3.5 (H_2_)	200	30.0	8	45.2	[207]
Cu-MgO	2.0–8.0 (H_2_)	4.0 (H_2_)	200	16.7	8	45.5	[167]
CuCr_2_O_4_	0.3–2.0 (H_2_)	1.4 (H_2_)	200	20.0	24	46.5	[45]
Cu-ZrO2-MgO	2.0–5.0 (H_2_)	4.0 (H_2_)	180	16.7	8	59.5	[148]
Pt/Fe_3_O_4_	2.5–5.0 (H_2_)	2.0 (H_2_)	230	30.0	16	64.2	[117]
Ni/NaX	3.0–7.0 (H_2_)	6.0 (H_2_)	200	20.0	10	69.6	[225]
Cu/Al_2_O_3_	3.5–8.7 (H_2_)	4.0 (H_2_)	200	16.7	24	72.5	[154]
Ni/WO_3_-MSEP	1.0–4.0 (H_2_)	2.0 (H_2_)	180	13.3	6	85.4	[231]
Cu/SiO_2_	4.0–7.0 (H_2_)	6.0 (H_2_)	210	14.7	8	85.6	[204]
Cu/ZnO/Al_2_O_3_	1.3–2.7 (H_2_)	2.7 (H_2_)	200	20.0	24	87.2	[160]
Cu/MgO	1.5–6.0 (H_2_)	4.5 (H_2_)	210	12.5	12	89.4	[185]

m_gly_/m_c_: glycerol to catalyst mass ratio; Y_1,2-PG_: yield to 1,2-PG.

**Table 24:** Effect of pressure in continuous flow reactors using different catalysts.

Catalyst	Range (MPa)	P_optimum_ (MPa)	T (°C)	SV (h^−1^)	Y_1,2-PG_ (%)	Ref.
Cu-Zn/MgO	0.1–0.8 (H_2_)	0.72 (H_2_)	220	0.12 (WHSV)	82.9	[214]
Cu-Ni/γ-Al_2_O_3_	0.1–1.0 (H_2_)	0.75 (H_2_)	220	0.97 (WHSV)	88.0	[220]
Cu/MgO	0.1–1.0 (H_2_)	0.7 (H_2_)	220	1.20 (WHSV)	95.5	[216]

SV: space velocity; Y_1,2-PG_: yield to 1,2-PG.

**Table 25:** Effect of H_2_ flow in continuous flow reactors.

Catalyst	Range (cm^3^ min^−1^)	H_2_ Flow Optimum (cm^3^ min^−1^)	P (MPa)	T (°C)	SV (h^−1^)	Y_1,2-PG_ (%)	Ref.
Ru/SBA-15	180–240	240	0.1 (H_2_)	260	2.52 (WHSV)	15.0	[107]
Cu/SBA-15	30–90	90	0.1 (H_2_)	220	1.03 (WHSV)	84.6	[200]

SV: space velocity; Y_1,2-PG_: yield to 1,2-PG.

**Table 26:** Catalysts and reaction conditions used to obtain kinetic models based on the power law.

Catalyst	T (°C)	P_H2_ (MPa)	C_gly_ (wt.%)	Partial Orders of Reaction		Ref.
				α	β	
Pd-CuCr_2_O_4_	220	3–7	4.5–9.1	2.28	1.09	[137]
Co-ZnO	160–220	2–4	10–40	0.7355	0.5697	[251]
Cu-ZrO_2_-MgO	160–220	2–5	10–40	0.6069	0.6955	[148]
Ru-Re/SiO_2_	130	7.5	40	1	1 ^1^	[98]
Cu/SiO_2_	180–240	2–8	23.9–45.6	0.27	0.95	[268]
Cu-Zn-Cr-Zr	220–250	1–4	60–100	1	1^1^	[162]
Cu/ZrO_2_	175–225	2.5–3.5	2–8	0	1	[283]
Cu/MgO	190–230	3–6	20–60	1.20	n.d.	[281]
Cu-Ni/γ-Al_2_O_3_	180–220	3–6	20	1.02	n.d.	[283]

α: partial reaction order for glycerol; β: partial reaction order for hydrogen ^1^ referred to P_H2_.

**Table 27:** Kinetic expressions found for glycerol consumption based on LHHW-type models.

Catalyst	Kinetic Expressions of the Model for Glycerol Consumption	Ref.
Ru/C	$(-r_{\mathrm{gly}})=\frac{k_{\mathrm{gly}}C_{\mathrm{gly}}{}^{1.5}}{{1+K}_{\mathrm{gly}}{\;C}_{\mathrm{gly}}{+K}_{\mathrm{EG}}{\;C}_{\mathrm{EG}}{+K}_{1,2\mathrm{-PG}}{\;C}_{1,2\mathrm{-PG}}}$	[42]
Co-Pd-Re/C	$(-r_{\mathrm{gly}})=\frac{k_{f}{\;C}_{\mathrm{gly}}{\;C}_{\mathrm{OH}}{\;C}_{H2}^{2}}{C_{\mathrm{gly}}{\;C}_{\mathrm{OH}}{+K}_{H}{\;C}_{H2}{}^{3}}$	[282]
Ni-Raney	$(-r_{\mathrm{gly}})=\frac{{(k}_{1}{+k}_{2}{)\;K}_{\mathrm{gly}}{\;C}_{\mathrm{gly}}}{{1+K}_{\mathrm{gly}}{\;C}_{\mathrm{gly}}{+K}_{1,2\mathrm{-PG}}{\;C}_{1,2\mathrm{-PG}}{+K}_{\mathrm{EG}}{\;C}_{\mathrm{EG}}{+K}_{\mathrm{MeOH}}{\;C}_{\mathrm{MeOH}}}$	[284]
Ru-Re/C	$(-r_{\mathrm{gly}})=\frac{{\mathrm{wk}}_{1}C_{\mathrm{gly}}C_{H2}{}^{g}}{H_{H2}\;}—\;\frac{{\mathrm{wk}}_{2}C_{\mathrm{gly}}C_{H2}{}^{g}}{H_{H2}\;}—{\;\mathrm{wk}}_{5}C_{\mathrm{gly}}$	[278]
Ni-Cu/Al_2_O_3_	$(-r_{\mathrm{gly}})=\frac{k_{\mathrm{gly}}C_{\mathrm{gly}}}{{1+K}_{\mathrm{gly}}{\;C}_{\mathrm{gly}}{+K}_{1,2\mathrm{-PG}}{\;C}_{1,2\mathrm{-PG}}}$	[285]
Pt/C	$\left(-r_{\mathrm{gly}}{)=w}_{\mathrm{cat}}{\;(r}_{1}{+r}_{2}{+r}_{4}{+r}_{5}\right)$ $r_{1}=\frac{k_{s1}K_{\mathrm{gly}}{\;C}_{\mathrm{gly}}{\;C}_{\mathrm{OH}}}{{{(1+K}_{\mathrm{gly}}{\;C}_{\mathrm{gly}}{+K}_{\mathrm{EG}}{\;C}_{\mathrm{EG}}{+K}_{\mathrm{OH}}{\;C}_{\mathrm{OH}})}^{2}}$ $r_{2}=\frac{k_{s2}K_{\mathrm{gly}}{\;C}_{\mathrm{gly}}{\;C}_{\mathrm{OH}}}{{{(1+K}_{\mathrm{gly}}{\;C}_{\mathrm{gly}}{+K}_{\mathrm{EG}}{\;C}_{\mathrm{EG}}{+K}_{\mathrm{OH}}{\;C}_{\mathrm{OH}})}^{2}}$ $r_{4}=\frac{k_{s4}K_{\mathrm{gly}}{\;C}_{\mathrm{gly}}{\;C}_{\mathrm{OH}}}{{{(1+K}_{\mathrm{gly}}{\;C}_{\mathrm{gly}}{+K}_{\mathrm{EG}}{\;C}_{\mathrm{EG}}{+K}_{\mathrm{OH}}{\;C}_{\mathrm{OH}})}^{2}}$ $r_{5}=\frac{k_{s5}K_{\mathrm{gly}}{\;C}_{\mathrm{gly}}{\;C}_{\mathrm{OH}}}{{{(1+K}_{\mathrm{gly}}{\;C}_{\mathrm{gly}}{+K}_{\mathrm{EG}}{\;C}_{\mathrm{EG}}{+K}_{\mathrm{OH}}{\;C}_{\mathrm{OH}})}^{2}}$	[279]
Cu-ZnO-Al_2_O_3_	$(-r_{\mathrm{gly}})=\frac{k_{1}K_{\mathrm{gly}}{\;C}_{\mathrm{gly}}}{{1+K}_{\mathrm{gly}}{\;C}_{\mathrm{gly}}{+K}_{\mathrm{AcOH}}{\;C}_{\mathrm{AcOH}}{+K}_{1,2\mathrm{-PG}}{\;C}_{1,2\mathrm{-PG}}}$	[175]
Cu-Ni/γ-Al_2_O_3_	$(-r_{\mathrm{gly}})=\frac{k_{\mathrm{gly}}{\;K}_{\mathrm{gly}}{\;P}_{\mathrm{gly}}}{{1+K}_{\mathrm{gly}}{\;P}_{\mathrm{gly}}{+(P}_{\mathrm{AcOH}}{/K}_{\mathrm{AcOH}}{)+(P}_{1,2\mathrm{-PG}}{/K}_{1,2\mathrm{-PG}})\;}$	[215]
	$(-r_{\mathrm{gly}}{)=k}_{\mathrm{gly}}{\;C}_{\mathrm{gly}}\;C_{H2}+k_{1,2\mathrm{-PG}}{\;C}_{\mathrm{gly}}\;C_{H2}$ $(-r_{\mathrm{gly}})=\frac{k_{\mathrm{gly}}{\;K}_{\mathrm{gly}}{\;P}_{H2}\;C_{\mathrm{gly}}}{{1+K}_{\mathrm{gly}}{\;P}_{\mathrm{gly}}{+(K}_{H2}{\;P}_{H2}{)}^{1/2}{+(C}_{1\mathrm{-POH}}{/K}_{1\mathrm{-POH}}{)+(C}_{1,2\mathrm{-PG}}{/K}_{1,2\mathrm{-PG}})}$	[280]

## Data Availability

Data reported were taken from papers included in the references.
